# Modelling the active SARS-CoV-2 helicase complex as a basis for structure-based inhibitor design†

**DOI:** 10.1039/d1sc02775a

**Published:** 2021-09-06

**Authors:** Dénes Berta, Magd Badaoui, Sam Alexander Martino, Pedro J. Buigues, Andrei V. Pisliakov, Nadia Elghobashi-Meinhardt, Geoff Wells, Sarah A. Harris, Elisa Frezza, Edina Rosta

**Affiliations:** Department of Physics and Astronomy, University College London London WC1E 6BT UK e.rosta@ucl.ac.uk; Department of Chemistry, King's College London London SE1 1DB UK; Computational Biology, School of Science and Engineering, School of Life Sciences, University of Dundee Dow Street Dundee DD1 5EH UK a.pisliakov@dundee.ac.uk; Department of Chemistry, Technische Universität Berlin 10623 Berlin Germany n.elghobashi-meinhardt@campus.tu-berlin.de; UCL School of Pharmacy, University College London 29/39 Brunswick Square London WC1N 1AX UK g.wells@ucl.ac.uk; School of Physics & Astronomy, University of Leeds Leeds LS2 9JT UK s.a.harris@leeds.ac.uk; Université de Paris, CiTCoM, CNRS F-75006 Paris France elisa.frezza@parisdescartes.fr

## Abstract

The RNA helicase (non-structural protein 13, NSP13) of SARS-CoV-2 is essential for viral replication, and it is highly conserved among the *coronaviridae* family, thus a prominent drug target to treat COVID-19. We present here structural models and dynamics of the helicase in complex with its native substrates based on thorough analysis of homologous sequences and existing experimental structures. We performed and analysed microseconds of molecular dynamics (MD) simulations, and our model provides valuable insights to the binding of the ATP and ssRNA at the atomic level. We identify the principal motions characterising the enzyme and highlight the effect of the natural substrates on this dynamics. Furthermore, allosteric binding sites are suggested by our pocket analysis. Our obtained structural and dynamical insights are important for subsequent studies of the catalytic function and for the development of specific inhibitors at our characterised binding pockets for this promising COVID-19 drug target.

## Abstract 2

The RNA helicase (non-structural protein 13, NSP13) of SARS-CoV-2 is essential for viral replication, and it is highly conserved among the *coronaviridae* family, thus a prominent drug target to treat COVID-19.

## Graphical abstract

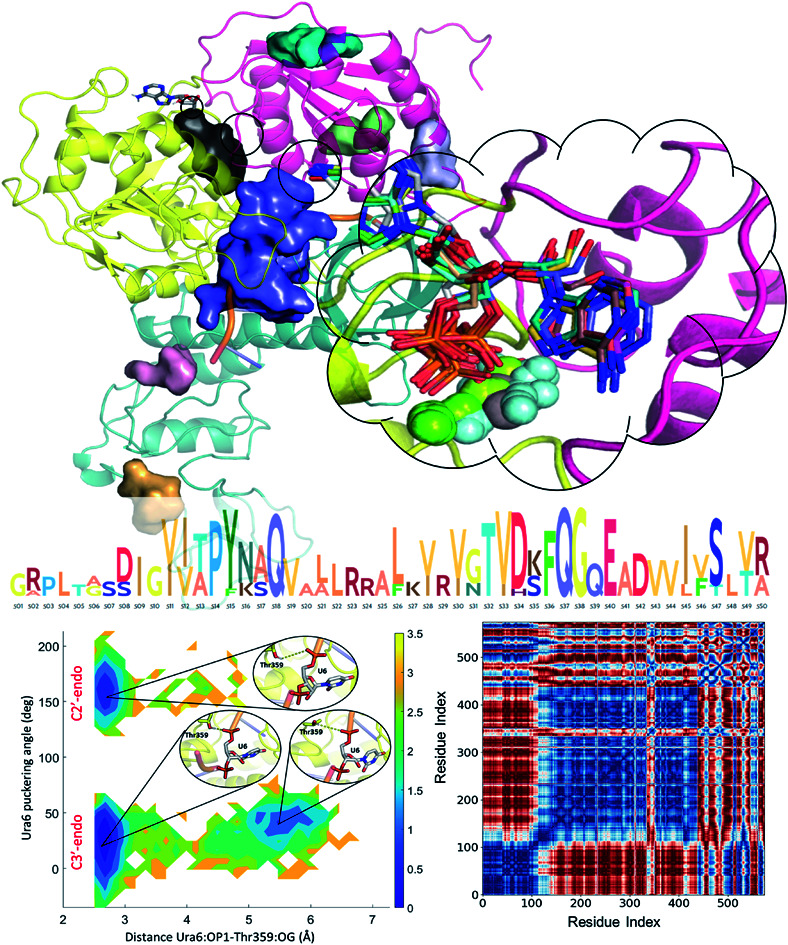

## Introduction

Severe Acute Respiratory Syndrome Coronavirus 2 (SARS-CoV-2) is the causative agent of COVID-19 disease and is responsible for the largest modern pandemic. The virus is closely related to SARS-CoV and MERS-CoV that caused smaller outbreaks of disease earlier this century.^1^ Currently, only a few approved drugs have been repurposed for the disease.^2^ The approved treatments can be categorized into three groups: aiding respiration in severe cases, repurposed antiviral drugs and monoclonal antibodies. Remdesivir has the longest history of use against COVID-19 infection and shown to bind to the RNA dependent RNA polymerase (RdRp),^3,4^ although recent trial data found no evidence for improvement in patient conditions.^5^ Further efforts include trials of antiviral combination therapies,^6^ or use of the anti-leprosy drug clofazimine, the latter has been found to inhibit helicase activity.^7^ However, there is a need for development of specific compounds that can be used to inhibit viral replication for the treatment of COVID-19, prophylaxis of vulnerable individuals and to add to the repertoire of treatment for future coronavirus outbreaks.

**Fig. 1:**
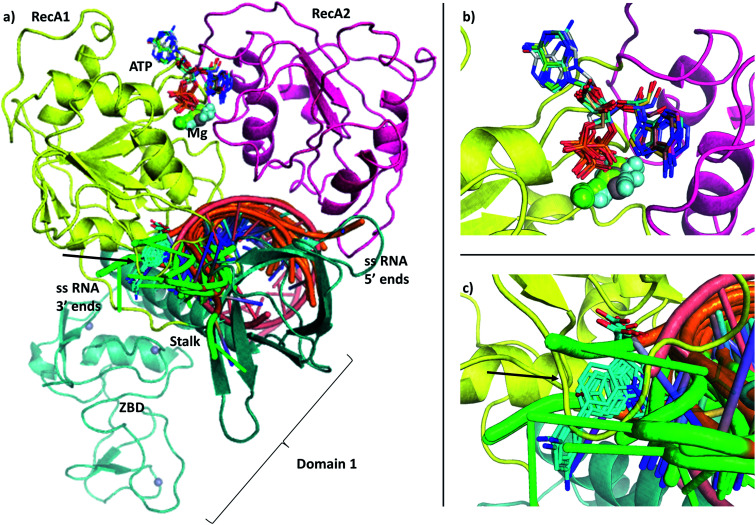
(a) Our model of the RNA helicase NSP13 of SARS-CoV-2 monomer (cartoon) coloured by three domains: RecA1 (yellow), RecA2 (magenta), and Domain 1 (aquamarine). ATP analogues (sticks) along with Mg (green sphere) and single stranded nucleic acids are depicted from aligned homologous structures (full list of PDB codes are available in Table S1†). 3′ ends of the nucleic acids present the same orientation in all chains (highlighted in green). (b) Position of the ATP analogues (nucleotides in stick and metal ions and compounds in spheres) in homologous structures. (c) Specific helicase inhibitor binding region with allosteric inhibitors displayed in cyan (black arrow).

Here we focus on determining the structure of catalytically active complexes of the SARS-CoV-2 RNA helicase, also known as non-structural protein 13 (NSP13) (Fig. 1). This protein is part of the Orf1ab polyprotein, that is spliced to produce the enzymes required for viral replication. The RNA helicase performs two essential functions for the viral replication making it an ideal drug target. It is thought to perform the first step in the 5′-capping of the viral RNA by its triphosphatase function hydrolysing the 5′-triphosphate group to form diphosphate-RNA.^8,9^ Furthermore, its main helicase function enables RNA translocation and unwinding in an ATP-dependent mechanism during viral replication.

Accordingly, numerous studies have already demonstrated that it is possible to develop potent inhibitors of viral helicases as antiviral agents.^10^ The 2003 SARS epidemic inspired a wave of drug development, often in conjunction with other positive RNA viral targets such as the hepatitis C virus (HCV).^11–14^ Consequently, HCV helicase inhibitory aryl diketoacids (ADKs) were found useful against SARS-CoV,^11,12,15^ in addition to porphyrin metal complexes,^16^ and natural^14^ or synthetic products.^13,17–19^ Typically, these compounds inhibit both the unwinding and the NTPase activity of the coronaviral helicase, but there are rare examples for selectively hindering the unwinding,^13^ or the NTP hydrolysis.^14^ Only a few of these efforts were based on or considered structural information. Notably, Hoffmann *et al.* build a homology model and proposed some lead compounds that may interact with the ATP site.^20^ Based on the effort on the SARS-CoV helicase, similar approaches can be applied to the highly homologous SARS-CoV-2 helicase.^7^

Coronaviral RNA helicases share a high similarity. 600 out of the 601 residues of the SARS-CoV-2 RNA helicase are identical to those of the SARS-CoV virus, and 70% match that of the MERS-CoV NSP13, demonstrating that these proteins are highly conserved within the *coronaviridae* family. Recently, a set of deposited structures from the PanDDA analysis group (to be published) deposited 51 high resolution crystal structure of the apo SARS-CoV-2 helicases in complex with a library of small molecule fragment analogues.

### Helicase structures and models

**Fig. 2:**
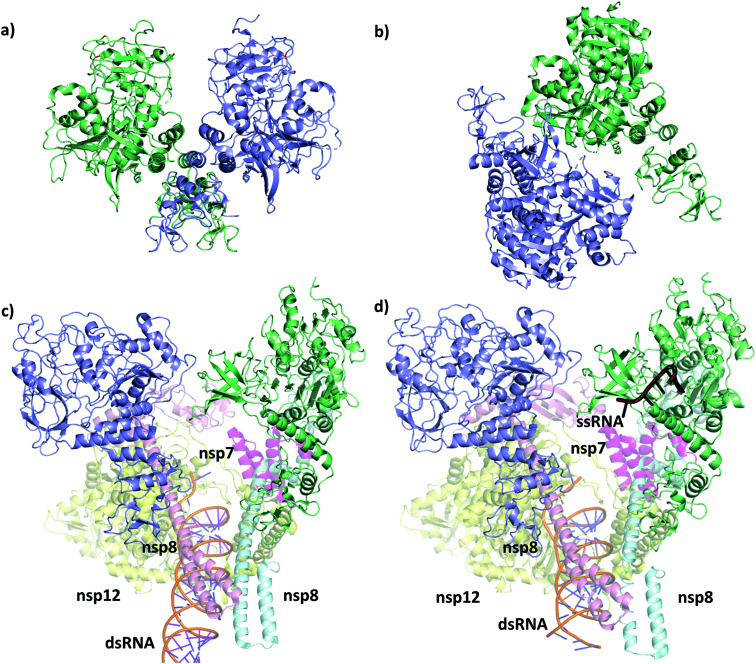
Structural comparison of the deposited PDB structures of the helicase dimer in SARS-CoV-1 (PDBID: 6jyt, a), SARS-CoV-2 (PDBID: 6zsl, b), SARS-CoV-2 in complex with NSP7 NSP8 and NSP12 (PDBID: 6xez, c) and SARS-CoV-2 with a small fragment of ssRNA bound in complex with NSP7 NSP8 and NSP12 (PDBID: 7cxm, d). The interaction between the two helicase monomers differs depending on the experimental method used to resolve the structures.

The first SARS-CoV-2 helicase structure (PDB ID 6zsl) was deposited in July 2020 and the almost identical SARS-CoV helicase structure in 2019 (PDB ID 6jyt),^21^ both resolved as crystallographic dimers (Fig. 2a and b). Interestingly, the dimerization interface is different in the two cases, leading to structurally dissimilar complexes. Recent works mainly focusing on the RdRp NSP12,^22,23^ which is expressed in the polyprotein sequence just before the helicase, also yielded structures of the replication machinery, including low resolution cryo-EM images of the helicase. In the cryo-EM structure of the RdRp complexed with the RNA helicase (and cofactors NSP7 and NSP8), the two helicase protomers mainly interact with NSP8 (Fig. 2c).^4^ The helicase chains were resolved using the apo helicase 6jyt as a template for the cryo-EM density maps and refined using software algorithms.^23^ Unfortunately, the 3.7 Å resolution is too low in this structure to resolve the ATP pocket in a catalytically competent conformation.

A more recent high resolution (2.90 Å) cryo-EM work of Lou *et al.*, presents a partial structure of the replication and transcription complex (RTC) (Fig. 2d).^24^ The complex includes the RdRp, NSP7, NSP8 and two helicase NSP13 protein copies, with one of the helicases in complex with a single-stranded RNA (ssRNA) fragment. Considering these structure, we conclude that catalytically active form of the helicase is a monomer within the larger RTC complex, and the crystallized homodimeric forms are not the biologically functional unit. Even more recently, a structure (PDB ID 7NNO) was released as a monomer, binding an ATP analogue ANP. The active site agrees with our model, however, this structure does not host ssRNA.

Although more attention is directed to targeting the RdRp^22,25–27^ or the main protease,^28–32^ as these are suggested to be more susceptible for binding an inhibitor,^33–35^ the helicase is also subject to modelling and docking studies. Crucially, however, the pharmacophore and docking studies of the helicase start from the homologous SARS-CoV crystal structure (Fig. 2a)^36–40^ or the counterpart in SARS-CoV-2 (Fig. 2b),^41^ which are both apo, lacking the ATP and the ssRNA from the complex. MD simulations are also available with ATP and ssRNA that used a docking approach to identify promising bound ligands at the ATP binding site.^42^

Here we present a computational model of the SARS-CoV-2 RNA helicase with ATP and ssRNA substrates bound. We performed sequence similarity searches to identify key domains and homologous sequences suggesting structurally important conserved motifs. We also performed structural alignments of available homologous helicase crystal structures to help position the bound RNA and ATP substrates (Fig. 1b). Using both Amber and CHARMM force fields, we carried out long timescale MD simulations of both the apo and the substrate bound states to address the flexibility and the stability of our catalytically competent structures. We analysed the differences in the dynamics between the apo and the holo structures using Principal Component Analysis (PCA). We identified the substrate and allosteric binding pockets and developed an implementation to follow their dynamical behaviour during the MD simulations. We demonstrate novel pockets, including ones that are coronaviridae-specific. Our results will help guide ongoing drug development.

## Methods

### Homology modelling

Proteins with crystal structures were aligned with MUSTANG for a combined structural-sequence alignment.^43^ The apo SARS-CoV-2 helicase structure was based on PBD ID 6jyt.^21^ Missing residues were added and the I570V replacement were carried out in Pymol 2.3.0.^44^ The positions of the Mg^2+^ and ATP were determined using the coordinates of PDB ID 2xzo,^45^ as a template. Crystallographic water residues were also taken from 2xzo as well as residues around the ATP pocket (loops 284–289 and 534–541, Gln404 and Arg443), except for Arg442 which was modelled based on PBD ID 6jim.^46^ The ssRNA was positioned based on 2xzl.^45^ The protonation state of titratable residues were estimated by PROPKA 3.0 (Tables S2 and S3†).^47,48^

### Molecular dynamics

We performed multiple unbiased MD simulations of the helicase in its apo and holo complex. For a detailed explanation of the methods used to parametrize and run the simulations, please refer to the ESI note 1 and Table S4† for a list of all simulations. We performed the MD using three independent force field setups: (1) CHARMM36 combined with TIP3P water potential (CHARMM), (2) Amber14SB for the protein, Amber ff99 + parmbsc0 + chioL3 for the ssRNA and TIP3P for water (Amber), and (3) Amber14SB protein force field combined with ff99OL3 for the RNA and TIP3P for water. We compared the simulations produced with (1) and (2) as part of the analysis and check the convergency by calculating the RMSD (Fig. S1†).

### Pocket analysis

MD trajectories were sampled at 1 ps intervals and stripped of all non-protein residues for pocket analysis. All pockets above the volume of 200 Å^3^ were obtained by using pyvol,^49^ with default parameters (sphere radius 1.4–3.4). Pocket equivalency across frames were based on Euclidean distance measured from every tenth α carbon of the protein backbone.

### Principal component analysis

We used PCA to assess the conformational changes observed in the monomer molecular dynamics simulations.^50^ The analysis was restricted to the α-carbon protein atoms to reduce the dimensionality of the dataset,^51^ and the protein chain was truncated to limit the contribution of end effects.

The data was grouped by the force-field used (CHARMM/Amber), and whether it was an apo or holo structure. The PCA was performed on these large groups using the scikit-learn library.^52,53^ Before the decomposition, each protomer in each frame from each simulation was aligned *via* RMSD minimization to a reference structure from the equilibrated holo model.

Weighted RMSD modes *N*_*i*_ were calculated to show the contribution of each of the *m* residues to the *i*th PCA mode using the following equation:^51^
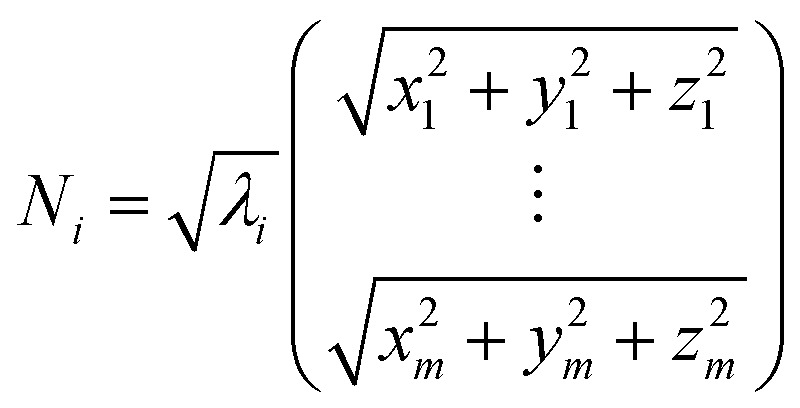


Where *λ*_*i*_ is the *i*^th^ eigenvalue, and the vector rows correspond to the coordinates describing the positions of each of the *m* α-carbon atoms. Component-wise decomposition of this vector gives a quantitative assessment of how much each residue influences the respective PCA mode.

We used dynamic cross-correlation (DCC) map analysis to determine inter-residue displacement correlations, calculated using:^54,55^
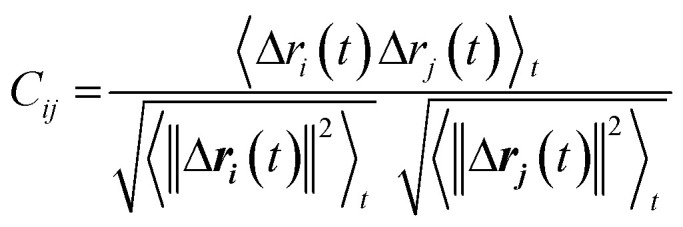
Where ***r***_***i***_(*t*) is the *i*th atoms coordinates at time *t*, 〈*x*〉_*t*_ denotes the time ensemble average of the quantity *x*, and **Δ*r***_***i***_(*t*) = ***r***_***i***_(*t*) − 〈***r***_***i***_(*t*)_*t*_〉.

This equation yields a scalar quantity *C*_*ij*_ for each pair of atoms, in the range 1 to −1. The closer the value to 1, the more the displacement of atom *i* is correlated to that of *j*. Similarly, a negative value indicates an inverse correlation between the two displacements, and a zero value indicates there is no correlation. The maps indicate which residues are displaced together, highlighting groups of residues that move as larger units.

### Dynamic weighted histogram analysis method (DHAM)

To calculate the free energy surface for the protein conformational landscape corresponding to key dynamical variables, we constructed a discretized two-dimensional grid to determine Markov State Models (MSMs).^56^ The collective variables were extracted along the trajectory, by calculating parameters, including inter-atomic distances, puckering angles, PCA components and pocket volumes. The 2D free energy surfaces are calculated from the first eigenvectors of the MSMs, and provide thermodynamic information on the collective variables used.

## Results

### Helicase domains and their sequence homology

The single-chain SARS-CoV-2 helicase can be divided into five domains.^21^ The sequence starts with Domain 1 (residues 1–260), which features: a Zinc-binding domain (ZBD, residues 1–100), known to facilitate nucleic acid recognition;^57^ a Stalk region shaped by 2 contiguous alpha helices (residues 100–150) which functions as an interface connecting the ZBD with Domain 1B (residues 150–260) that interacts with the ssRNA. For simplicity, we refer to domain one as these three combined (Fig. 1, aquamarine cartoon). The rest of the chain is divided into RecA1 and RecA2 domains,^58^ which are well characterized in the superfamily 1B-type helicases and interact with ATP at their interface (Fig. 1, yellow and magenta cartoon, respectively).

We have obtained the most homologous 957 sequences and their alignments from the UniProtKB library.^59^ The pairwise alignments showed flawed identification of RecA1 (see Fig. S2†), therefore we proceeded to optimize a multiple sequence alignment (MSA).^60^ Firstly, we clustered the obtained sequences to avoid overrepresenting highly similar entries.^61^ The full list of 796 clusters is available in the ESI note 2.

**Fig. 3:**
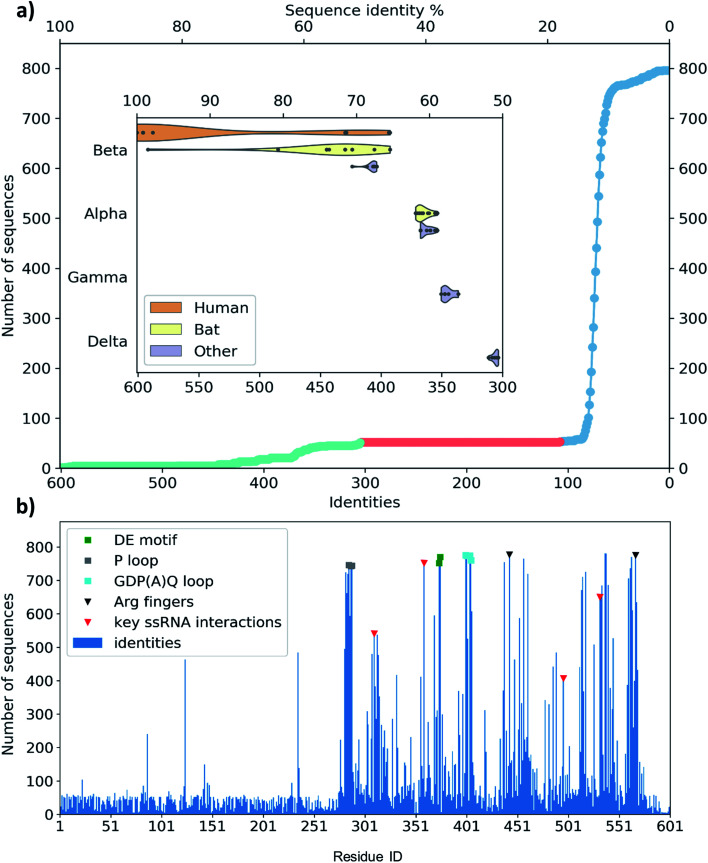
(a) Distribution of the identity of sequences in multiple sequence alignment compared to the SARS-CoV-2 helicase. There are only members of coronaviridae above 300 matching residues (50%, lime circles, 52 entries). There are no sequences with medium similarity (107–300 similar residues, red circles). The mass of the sequences matches only 107 residues or less (blue circles). The closest relatives (96 sequences represented by 52) are grouped in coronavirus subfamilies (grouped in the *y* axis) with principal hosts highlighted in the inset. (b) Sequence identity of the representative sequences of 796 clusters from UniProtKB aligned to the 601-residue long SARS-CoV-2 RNA helicase. Domain 1 shows similarity only to the close relatives (52 sequences, representing clusters of 96), while the RecA1 and RecA2 domains are more common across ATPase sequences. Key structural motifs are highlighted using symbols (P-loop: grey square, DE motif: green square, arginine fingers: black triangle, ssRNA interactions: red triangles).

A set of 52 of these clusters and their representative sequences show similarity across the whole helicase sequence and match at least half of the helicase in the MSA (Fig. 3a, lime region). These represent 96 sequences, all derived from coronaviruses, primarily originate from human and bat viromes (beta and alphacoronaviruses),^62^ and infect various hosts in the animal kingdom, including humans. Intriguingly, the next best sequence alignment only matches 107 amino acids; most of these and subsequent aligned regions are specific to the RecA domains and span all types of proteins from various organisms (Fig. 3a blue region).

To evaluate any similarities to Domain 1 only, we also performed a search using only the first 230 residues. This search for sequences that match at least 70 residues resulted in the exact same 96 sequences as before, exclusively belonging to *coronaviridae*. An additional 21 sequences match only shorter segments of the sequence between residues 1–230, corresponding to a 22% sequence identity or below.

The MSA also enables us to see which regions and motifs are conserved in the SARS-CoV-2 helicase sequence. Domain 1 only exhibits a few residues apparently with higher conservation (at positions 87, 124 and 235), however, these are likely to be only random matches, as the corresponding alignments are dominated by non-coronaviral sequences, and not aligned well to the neighbouring residues of the SARS-CoV-2 helicase sequence. The RecA domains, which are members of the *AAA 30* and *AAA 12* families, are more common in various ATP-binding structures and the MSA indeed sheds light upon important binding motifs such as the P-loop (Fig. 3b, grey squares) the DE motif (Fig. 3b, green squares) and the arginine fingers (Fig. 3b, black triangles). Furthermore, we identified several residues involved in RNA binding (Fig. 3b, red triangles) which are also conserved structurally in homologous PDB entries. Finally, the GDP(A)Q loop at position 400–404 with high consensus in the MSA features a glutamine potentially involved in the proton transfer during the ATP hydrolysis. This motif bridges the γ-phosphate end of the ATP pocket and the RNA binding site; therefore we suggest that it may be involved in coupling the hydrolysis of ATP to the changes induced by the hydrolysis inducing the RNA translocation.

Among crystal structures containing ATP analogues, most helicases have very low sequence similarity to NSP13. The closest homologues are 2xzo, 5mzn, and 6jim with 11.0%, 10.2%, and 8.4% sequence identity, respectively. Despite the low sequence identity, most residues in the ATP binding pocket are conserved. At the same time, the closest human sequence homologue based on our homology search, ZGRF1, a putative RNA helicase, shares only 22% sequence similarity, restricted to the RecA1 and RecA2 domains. This relatively narrow bandwidth of sequence similarity may be advantageous to the design of specific inhibitors against the coronavirus RNA helicases that do not inhibit human proteins.

### Structural model of the ATP binding site

**Fig. 4:**
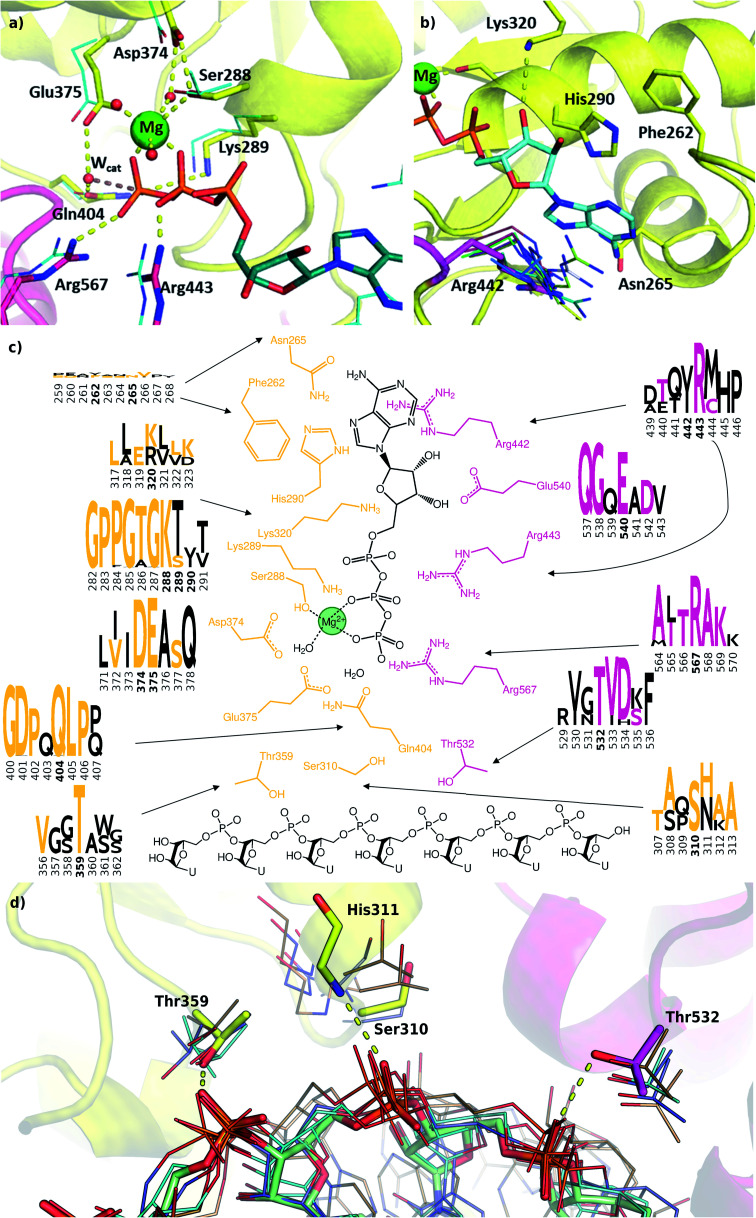
Modelling and conservation of the ATP and RNA sites. (a) Main protein-substrate interactions of the triphosphate and magnesium ions are compared with alignment for PDB template 2xzo (cyan lines). (b) Nucleotide-binding region focusing on Arg442 (magenta sticks) is aligned with homologous arginine residues (lines, PDB structures 5k8u, 5vhc, 5xdr, 5y4z, 5 y6m, 5y6n, 6adx, 6ady, 6c90 and 6jim). (c) Sequence conservation for RecA1 (orange) and RecA2 (magenta) domains are depicted in logos for each residue and its neighbours (data from Fig. 3b). Coloured letters represent the residues in the SARS-CoV-2 helicase sequence, depicted residue indices are bold in the logos. (d) Structures of the RNA binding region aligned with existing RNA-helicase crystal structures complexed with ssRNA (depicted in lines). RecA1 and RecA2 domains are shown in yellow and magenta, respectively. Key residues (sticks) are labelled, and H-bonds are depicted in yellow dashes.

We modelled the ATP-bound active site of the SARS-CoV-2 helicase using the 2xzo structure as a template.^45^ The essential Mg^2+^ ion cofactor coordinates both the β- and γ-phosphates and a conserved Ser288 (Fig. 4a and c). The active site contains a DE of the DEAD-motif of RNA helicases. The conserved Asp374 H-bonds with Ser288 and one of the Mg-coordinating water molecules, whereas the Glu375 is positioned as the proton acceptor.^20,63,64^ The γ-phosphate is stabilized *via* electrostatic interactions and H-bonds with Arg567 Lys289 and Gln404 through a water molecule, which are also found in 95, 56 and 57% of the homologous sequences analysed, respectively. The β-phosphate forms a H-bond with Arg443. Unlike the highly conserved residues recognizing the triphosphate pocket, the environment of the sugar and purine moieties (Fig. 4b and c) shows a greater diversity. The ATP ribose is likely to interact with Glu540 and Lys320 as seen in ten and four homologous PDB structures, respectively. The purine ring is stabilized through multiple π stacking interactions, from one side with Arg442 (a π-cation interaction), in some helicases this interaction is fulfilled with a tyrosine residue; from the other side with His290 and Phe261. Additionally, there is a H-bond between the exocyclic amino group of the purine with Asn265, a residue which is more typically served by a glutamine in similar sequences.

A lack of specificity towards the purine group is likely due to the dual function of the SARS-CoV-2 helicase to aid the 5′-capping of the RNA by the triphosphate hydrolysis of most NTP substrates.^9^ Due to these major differences, this area of the nucleotide-binding pocket may be useful in the design of SARS-CoV-2-specific antiviral drugs.

## Structural model of the RNA binding site

The most significant changes between the holo and apo structures are related to the binding of the large ssRNA substrate. This substrate binding is more challenging to model, partly due to potential force field inaccuracies, and partly also due to the less specific interactions between the protein and the RNA sidechain that has to accommodate a range of viral sequences for the unwinding and translocation function of the helicase. Despite the relatively large size of the ssRNA substrate, we did not observe large scale domain movements in the holo structure compared to the apo. We observed more localized conformational changes: only the loop of residues 482–487 and the C terminus of RecA2 domain changed considerably compared to the apo structure.

Filtering the related crystal structures those containing nucleic acids (NAs), we noticed that their directionality relative to the ATP pocket is well defined (Fig. 1a and c). Domain 1, being in contact with the sidechain of the ssRNA, does not feature specific motifs, thus allowing different RNA bases to translocate. A long loop transitions into the RecA1 and 2 domains sandwiching the ATP pocket on the side of the ssRNA backbone. This region, equipped with the necessary functionalities to perform the ATP hydrolysis, has a higher degree of conservation along the helicases. Both RecA domains have specific residues that contact ssRNA phosphates, depicted in Fig. 4c and d. Thr359 in RecA1 and Thr532 are identified as the main anchoring points of the two domains. The base between these two threonine residues is coordinated by the backbone NH of His311, an interaction which is conserved in NA containing crystal structures. Ser310 is also reasonably conserved, although not directly involved in ssRNA coordination in this state of the enzyme.

Interestingly, the most conserved motif across the sequences is a GDP(A)Q loop interfacing between the RecA1 and RecA2 domains. This motif features Gln404, a residue which we consider to be important in the coordination of the nucleophilic water in the ATP binding pocket; moreover, it bridges the ATP γ-phosphate and the SH motif discussed earlier. We speculate these moieties play a role in the translocation of the RecA2 unit upon ATP hydrolysis.

### MD simulations

#### ATP binding site

We extracted nine key distances (Fig. 4a and S3†) along the simulations from our unbiased trajectories of the ATP-ssRNA-helicase (holo) complex. There is an overall good agreement between the two force fields for the distribution of these contacts. The hexacoordinate Mg^2+^ shows stable coordination to 3 water molecules, the OH of Ser288 and oxygens from the β- and γ-phosphates of the ATP (Fig. S3a–c†), which is essential for the preorganization of the ATP hydrolysis. Further conserved contacts in the pocket are also maintained during the simulation including the arginine fingers (Fig. S3g and h†), Lys288 (Fig. S3i†) and the DE motif (Fig. S3d and e†) which both takes part in coordinating the Mg^2+^ and the nucleophilic water. The largest deviations between CHARMM and AMBER force fields are observed for the Gln404 and ATP distance (Fig. S3f†). This is a particularly important conserved residue that likely coordinates the attacking water. Using the CHARMM fore field, Gln404 shows greater flexibility deep in the ATP pocket, which might support a role in changing the protein conformation during translocation. Residues participating in the adenosine base coordination are less conserved and form fewer stable contacts.

#### RNA binding site

From the structural analysis and the homology modelling, we denote two important and well-conserved interactions between the ssRNA and the helicase. Both interactions involve a H-bond between a threonine (Thr359 from RecA1 and Thr532 from RecA2) and a phosphate oxygen on the backbone of the ssRNA. Additionally, another H-bond is made between the central RNA residue and the N of His311, this residue is moderately conserved and the interaction between its backbone and the phosphate oxygen of the RNA is present in several PDB structures. A key residue close to the RNA pocket is Ser310; this residue is conserved (often present as a threonine) and appears to be important for the communication between the ATP pocket and the RNA pocket (Fig. 4c and d).

**Fig. 5:**
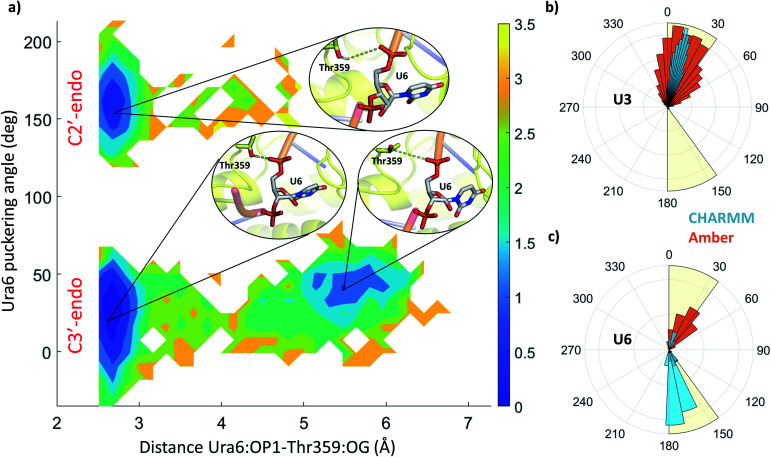
(a) 2D free energy profile along with the puckering angle and the distance between uracil 6 (OP1) and threonine 359 (OG), from all Amber holo simulations. The colour bar represents the hight of the free energy profile in kcal mol^−1^. Insets depict the structures in the three local minima, showing uracil 6 in grey sticks and threonine 359 as yellow sticks. Specific distance between the residues is highlighted by yellow dashes. (b and c) Distribution of the puckering angle along the MD simulations using CHARMM (blue) and Amber (orange) force field for uracil 3 (b) and 6 (c).

The stability and dynamics of the RNA have been further analysed by looking at the furanose ring conformation. The definition of the envelope conformers and the puckering angles as descriptors of the conformation are discussed in the ESI Note 3. RNA nucleotides, differently from DNA nucleotides, usually adopt a C3′-endo configuration (usually defined as N), and they become less stable/more reactive when switching to a C2′-endo configuration (defined as S). From a structural comparison with the PDB structure with NA bound to helicase, we can see that most nucleotides present a C2′-end configuration (Fig. S5†). We calculated the puckering value in our MD simulations, as expected most of the nucleotides, present a C3′-endo configuration, relatively stable along with the simulations (Fig. 5 and S4†). Uracil 5, 6 and 7 present a bimodal distribution, showing during multiple trajectories both N and S configuration. Using 2D-DHAM, we calculated the free energy surface by correlating the puckering angle of uracil 6 and the distance between the phosphate oxygen of uracil 6 and the γ-oxygen of Thr532 (Fig. 5a). The reconstruction of the corresponding free energy profile is not possible using the CHARMM trajectories, because the transition between the puckering states is not sampled well. This implies that the CHARMM force field describes the RNA residues more rigidly. We also observe a difference in the orientation of the 3′ terminal residues (Ura7 and Ura8) between force fields, while the rest of the ssRNA behaves similarly and agrees well with the experimental structure (Fig. S6†).

### Principal component analysis

To understand the key structural components corresponding to the longer time-scale thermal motions of the protein in the holo (with both ATP and ssRNA bound) and apo forms, we performed principal component analysis. For all simulations analysed, the first four PCA components always accounted for greater than 80% of the observed variance, we therefore focused our analysis on these.

**Fig. 6:**
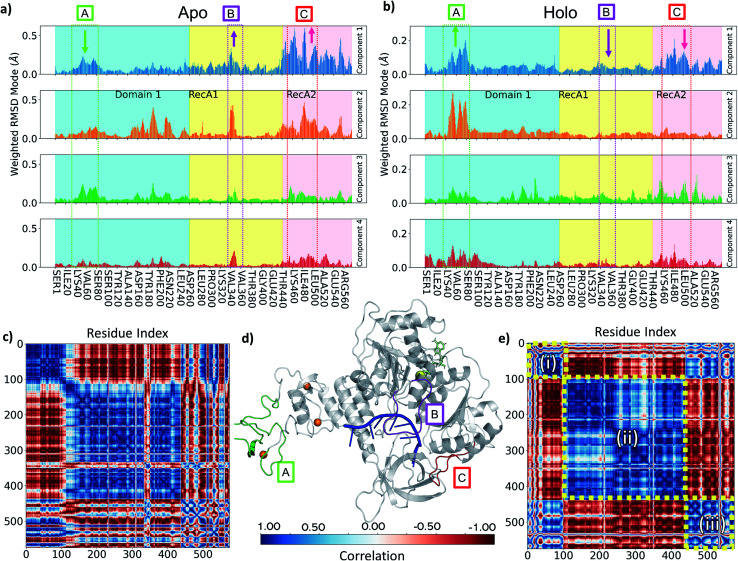
(a and b) Comparison of PCA Weighted-RMSD scores holo monomer (a) and apo monomer (b) simulations with Amber. (c) DCC map of holo monomer simulation with Amber. (d) Key areas highlighted in the helicase structure (d, grey cartoon) and on panels a and b (text box and arrows indicating relative change in magnitude): (A) Zinc Binding region (green), (B) Domain 1 loop from Ile334 to Gln354 (purple), (C) RecA1/2 interface loop from Thr450 to Ala510 (red). Bound objects are shown and coloured: RNA in blue (centre), ATP in lime (upper right), zinc in orange (left spheres) and magnesium as a yellow sphere. (e) DCC map of apo monomer simulation with Amber for motions described by the first principal component. Colour bar providing correlation scale is shown in the bottom centre.

Key observations are shown in Fig. 6, S7 and S8,† which provide a direct comparison between the apo and holo monomer simulations. The weighted RMSD modes for the first four PCA components show the residue displacements captured by each component (Fig. 6a and b and S7†) for the apo and holo simulations, respectively. To reveal the correlated motion within the protein, DCC maps were generated using the first PCA component, which accounts for the largest portion of the overall observed variance (Fig. 6c and e, and S8†).

Increased flexibility in the RecA2 domain is the most clear and significant difference present between the apo and holo data (Fig. 6a and b for Amber and, consistently, in Fig. S7† for CHARMM). Residues around the RecA1 interface and ATP binding site, most notably in the outermost loop from Thr450 to Ala510, are more flexible in the absence of ATP, as is clear from the magnitude of the associated weighted RMSD mode (Fig. 6a and d; label C, red). This observed flexibility is also consistent with the increased experimental beta-factors of this region for the 6jyt and 6zsl structures (ESI note 3 and Fig. S9†). On the other hand, the presence of ATP in the holo simulations stabilizes many of the key residues involved in binding along with the whole of the RecA2 domain (Fig. 6a and b), as it is indicated by the decreased weighted RMSD in the holo simulations and thus smaller contribution to all the PCA components.

The behaviour of the loop from Ile334 to Gln354 is another key difference between holo and apo simulations. This is more prevalent in the Amber simulation PCA components (Fig. 6a and d; label B, purple) and visible as the large cross in the DCC maps (Fig. 6c), which shows its motion differs from the rest of RecA1. Its position between both key RNA binding residues (Thr359 and Thr532) and ATP binding residues (such as Lys320) point towards the substrates providing some tension keeping this loop in place. The stable orientation and position of the sidechain Lys345 observed during holo simulations alludes to an interaction with either the nearby α-helix containing Lys320 or the ATP binding site itself.

In the holo simulations, PCA identifies more fluctuations from regions in the ZBD (Fig. 6b and d; label A, green). The holo DCC map (Fig. 6e) highlights the predominant conformational motion in the first PCA component, splitting the helicase into three correlated areas: (i) the ZBD domain, (ii) the rest of Domain 1 along with RecA1, and (iii) RecA2. The structure oscillates with the ZBD and RecA2 regions moving in correlation, opposing the larger central region. All bound simulations showed this global motion (Fig. S10†), displaying an increased flexibility in loops around the ZBD domain not directly involved in zinc-binding and a larger correlation across the motion of the RecA2 domain. However, while the scale of these movements does not indicate a domain level change, it is indicative of the protein flexibility otherwise not easily accessible from *e.g.*, crystallographic data, and disrupting this major motion may provide an aim for future inhibitor design.

### Pocket analysis

**Fig. 7:**
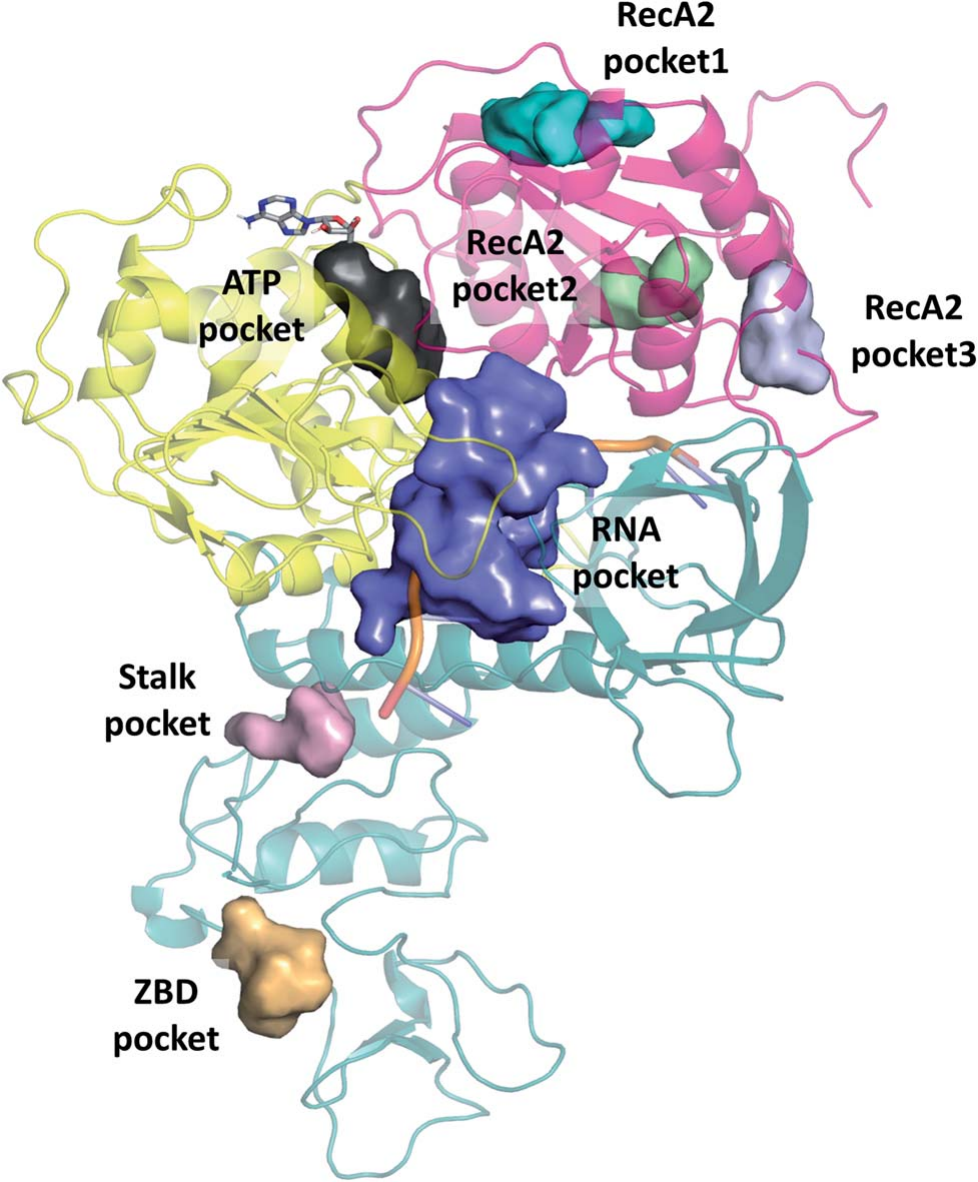
Overview of binding pockets (coloured surfaces) identified in the holo complex MD trajectories. ATP (grey sticks) and the ssRNA (cartoon) are only show for the sake of orientation.

We selected cavities that were consistently present in our MD simulations and tracked the changes in these pocket volumes during the trajectories (Fig. 7). The average volumes and corresponding standard deviations of the pocket sizes in different simulation types are also compared with available experimental structures in Table 1. The distributions of the pocket volumes are depicted in Fig. S9 and S10† for the holo and apo systems, respectively.

**Table 1:** Occurrence and volume statistics of pockets depicted in Fig. 7 in MD simulations and available PDB structures. The rows in the experimental structures represent the different chains. Note that the minimum volume for any pocket to be defined is 200 Å^3^

		Force field	ATP pocket	RNA pocket	RecA2 pocket1	RecA2 pocket2	RecA2 pocket3	Stalk pocket	ZBD pocket
(%)	Holo	CHARMM	96.28	98.60	18.45	22.25	54.57	26.52	16.91
		Amber	98.28	98.18	34.92	18.12	—	16.40	20.26
	Apo	Amber	81.40	99.74	35.06	—	37.00	17.77	7.89
Volume (stdev)/Å^3^	Holo	CHARMM	630 (263)	1472 (858)	276 (64)	409 (179)	405 (200)	449 (202)	268 (74)
		Amber	542 (223)	1911 (565)	330 (91)	353 (119)	—	300 (75)	246 (47)
	Apo	Amber	665 (338)	2415 (873)	303 (106)	—	286 (99)	282 (73)	247 (50)
	6jyt	X-ray	4058		334	204	—	—	—
			4427		1282^a^	—	1282^a^	—	—
	6zsl		474 + 267^b^	—	—	—	—	—	—
			613	—	—	—	—	—	—
	6xez	Cryo-EM	534	3698	662	—	—	279	—
			4891		540	—	—	—	—
	7cxm		708	2780	588	369	—	—	—
			782	1566	—	—	—	—	—

a Labelled pockets are connected, the combined volume is shown.

b Made of two separate pockets.

#### Substrate pockets

The ATP pocket is easily identified in the holo trajectories and its volume is ∼600 Å^3^. In the apo trajectories the standard deviation increases, indicating less constrained movement between RecA1 and RecA2. The RNA pocket is usually the largest cavity identified and ranges along the interface between the domains, largely overlapping with the RNA binding site. The volume of the RNA pocket increases from 1911 (1472 with CHARMM) to 2415 Å^3^ in the apo trajectories compared to the holo structures (Table 1), which can be attributed to the larger freedom in the movement of the domains as the RNA goes along the interface. Additional data on the apo dimer simulations are also available in Table S5.†

The 6jyt crystal structure features a connected ATP-RNA pocket in both of the monomer chains, as is often observed in our apo simulations (Fig. S13†). In general, the sizes of the ATP and RNA pockets agree between the crystal structures and our simulations, including a decrease in the RNA pocket size when ssRNA is bound to the structure (holo, 7cxm in Table 1).

#### Allosteric pockets

Generally, all non-substrate pockets are significantly smaller, and appear less frequently during the trajectories. Among the RecA2 pockets, pocket1 is the most consistent, while both pocket2 and pocket3 depends on the movement of the C-terminus of the chain and they are sensitive to the presence of the natural substrates or the force field (see Table 1 and S5†). Pocket1 is also found in most of the experimental structures by our pocket analysis.

Domain 1 hosts two interesting pockets, the ‘Stalk pocket’ and ‘ZBD pocket’. The Stalk pocket resides between the longer Stalk helix and the ZBD and can be consistently identified in all trajectories, although its average volume varies, probably because it is close to the N-terminus of the chain, which moves relatively freely. More importantly, several bound molecules were identified at this site experimentally in the helicase-small molecule crystal structures deposited in the PDB (5rli, 5rmd and 5rm1). The Stalk pocket is also identified in one of the cryo EM structures (PDB 6xez). The ZBD pocket is found in most trajectories but in the lowest consistency and average volume among all analysed pockets. The residues neighbouring these pockets are detailed Fig. S14†. Overall, both force fields identified the pockets with similar statistical parameters. One difference was in the additional pocket3 observed in RecA2 with CHARMM that is absent from Amber.

### Features influencing the pocket volume

We examined the correlation between the volume of the identified pockets and the residue–residue distances in the trajectories (ESI note 5 and Table S6†). This enables us to detect the mechanism and elucidate the precise structural interactions leading to these cavities opening up and closing during the MD simulations. Subsequently, we also calculated the quantitative free energy profiles related to these correlations using DHAM.

**Fig. 8:**
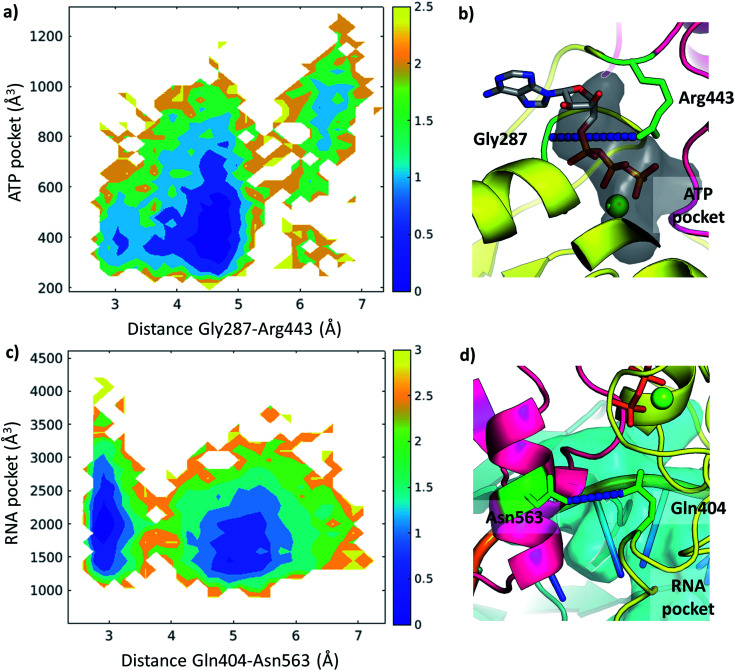
Free energy profiles depicted along selected coordinates and substrate pocket volumes (holo Amber trajectories). (a) 2D profile along Gly287-Arg443 distance and ATP pocket volume. The color bar represents the height of the free energy profile in kcal mol^−1^. (b) The ATP pocket (black surface) and the Gly287-Arg443 distance (residues in green, distance in blue) depicted in a representative structure. (c) 2D profile along Gln404-Asn563 distance and RNA pocket volume. The color bar represents the height of the free energy profile in kcal mol^−1^. (d) The RNA pocket (cyan surface) and the Gln404-Asn563 distance (residues in green, distance in blue) depicted in a representative structure.

The ATP pocket is located at the interface of RecA1 and RecA2, thus it primarily depends on the interactions of the contacting residues. We observed that the opening of the pocket can be described by the distance between Gly287 or the P-loop and the arginine finger 443, resulting in three connected local minima on the potential energy surface (Fig. 8a and b).

On the other hand, the RNA pocket is larger, it has more bordering residues and it can open up in multiple directions. Here, as one of the most prominent directions, we focus on the influence of the contact between Asn563 and Gln404, which is anti-correlated with the size of the RNA pocket. Gln404 is located in one of the most conserved motifs in the sequence (Fig. 3b) and it is connected to the ATP γ-phosphate (Fig. 4c), directly influencing the ATP hydrolysis. The increase of this Asn563-Gln404 distance decreases the optimal RNA pocket size by 500 Å^3^ down to 1500 Å^3^ (Fig. 8c and d).

## Conclusion

Here, we present structural and sequence comparison studies, as well as molecular dynamics simulations of both the apo and a catalytically relevant computational model of the SARS-CoV-2 NSP13 ATP dependent RNA helicase. The analysis of homologous sequences sheds light upon the specificity of the domain structure of the viral helicase yielding no match over 20% except close relatives from the coronaviridae family.

We performed extensive MD simulations of helicase monomers and a dimer. However, upon analysis of available experimental structures, including the RTC complex, we suggest that the dimer is not the functional unit, and we furthermore focused on the monomer.

To gain key insights into the structure and dynamics of the complete holoenzyme in addition to the experimentally available apo protein, we modelled a fully assembled complex with both the ATP and ssRNA substrates. The structure of the ATP pocket was reconstructed including signature motifs from phosphate binding proteins, such as the DE(AD) of helicases, the P-loop, and arginine fingers. We did not observe large scale domain level motions upon RNA binding. Nevertheless, some conformational changes are required to accommodate the RNA, which, compared to the ATP, does not have so well-defined interactions with the protein to enable multiple sequences to be processed. Moreover, more structural variations and uncertainties for the RNA are also observed in our models during the simulations. Importantly, we identified highly conserved anchoring points in the core of the helicase for polynucleotide binding, which are essential to understand the translocation driving the unwinding activity of NSP13. Our molecular dynamics simulations verified the stability of conserved interactions in our model, as well as improved our initial model to host the nucleic acid. Decomposing the trajectories into principal components highlighted the rigidifying effect of the substrates to the protein structure. The increased stability of our holoenzyme model should be exploited in subsequent docking studies, moving away from the apo structures.

We characterized the volume of the ATP and ssRNA pockets and, importantly, identified additional allosteric binding sites. We assessed the connection between the substrate pockets and key interactions therein, giving insights to the dynamic behaviour of the cavities. Importantly, we found pockets in the highly specific Domain 1 of the helicase which coincides with some experimentally bound substrates. This may provide a good opportunity for specific structure-based inhibitor design.

The comparison of different force fields resulted in small differences only. CHARMM presents a more rigid ssRNA model than Amber, leading to less structural diversity when considering the bound RNA conformation. The ATP binding on the other hand remains robust in all holo simulations with both force fields. The change in the dynamics of the protein upon substrate binding is similar, as well as the qualitative description of allosteric pockets.

Our work provides insight into a key participant of the SARS-CoV-2 viral replication machinery, one of the prominent drug targets. Our structures offer novel starting points for structure-based compound design and screening. The catalytically relevant holo structures are also ideal starting points for subsequent mechanistic studies of the ATPase and the unwinding activity of the helicase. Moreover, elaborating the RNA translocations driven by the identified interactions can reveal other targetable states of the helicase.

## Data availability

All simulation data is available at the MolSSI and SlimMD (https://www.ccpbiosim.ac.uk/slimmd) repositories. The pocket analysis code is available at https://github.com/bertadenes/pyvol.

## Author contributions

DB: conceptualization, formal analysis (structural and sequence data, MD, pockets), methodology, software; MB: formal analysis (MD), investigation, methodology; SAM: formal analysis (PCA), investigation, methodology; PJB: formal analysis (sequence data); AVP: supervision; NEM: resources, investigation, supervision; GW: investigation, supervision; SAH: resources, investigation, supervision; EF: resources, investigation, supervision; ER: conceptualization, formal analysis, resources, methodology, supervision. All authors contributed to the writing of the paper.

## Conflicts of interest

There are no conflicts to declare.

## Supplementary Material

SC-012-D1SC02775A-s001

## References

[cit1] Wang M., Yan M., Xu H., Liang W., Kan B., Zheng B., Chen H., Zheng H., Xu Y., Zhang E., Wang H., Ye J., Li G., Li M., Cui Z., Liu Y.-F., Guo R.-T., Liu X.-N., Zhan L.-H., Zhou D.-H., Zhao A., Hai R., Yu D., Guan Y., Xu J. (2005). Emerging Infect. Dis..

[cit2] Wu F., Zhao S., Yu B., Chen Y.-M., Wang W., Song Z.-G., Hu Y., Tao Z.-W., Tian J.-H., Pei Y.-Y., Yuan M.-L., Zhang Y.-L., Dai F.-H., Liu Y., Wang Q.-M., Zheng J.-J., Xu L., Holmes E. C., Zhang Y.-Z. (2020). Nature.

[cit3] Beigel J. H., Tomashek K. M., Dodd L. E., Mehta A. K., Zingman B. S., Kalil A. C., Hohmann E., Chu H. Y., Luetkemeyer A., Kline S., Lopez de Castilla D., Finberg R. W., Dierberg K., Tapson V., Hsieh L., Patterson T. F., Paredes R., Sweeney D. A., Short W. R., Touloumi G., Lye D. C., Ohmagari N., Oh M., Ruiz-Palacios G. M., Benfield T., Fätkenheuer G., Kortepeter M. G., Atmar R. L., Creech C. B., Lundgren J., Babiker A. G., Pett S., Neaton J. D., Burgess T. H., Bonnett T., Green M., Makowski M., Osinusi A., Nayak S., Lane H. C. (2020). N. Engl. J. Med..

[cit4] Chen J., Malone B., Llewellyn E., Grasso M., Shelton P. M. M., Olinares P. D. B., Maruthi K., Eng E., Vatandaslar H., Chait B. T., Kapoor T., Darst S. A., Campbell E. A. (2020). Cell.

[cit5] Wang Y., Zhang D., Du G., Du R., Zhao J., Jin Y., Fu S., Gao L., Cheng Z., Lu Q., Hu Y., Luo G., Wang K., Lu Y., Li H., Wang S., Ruan S., Yang C., Mei C., Wang Y., Ding D., Wu F., Tang X., Ye X., Ye Y., Liu B., Yang J., Yin W., Wang A., Fan G., Zhou F., Liu Z., Gu X., Xu J., Shang L., Zhang Y., Cao L., Guo T., Wan Y., Qin H., Jiang Y., Jaki T., Hayden F. G., Horby P. W., Cao B., Wang C. (2020). Lancet.

[cit6] Hung I. F.-N., Lung K.-C., Tso E. Y.-K., Liu R., Chung T. W.-H., Chu M.-Y., Ng Y.-Y., Lo J., Chan J., Tam A. R., Shum H.-P., Chan V., Wu A. K.-L., Sin K.-M., Leung W.-S., Law W.-L., Lung D. C., Sin S., Yeung P., Yip C. C.-Y., Zhang R. R., Fung A. Y.-F., Yan E. Y.-W., Leung K.-H., Ip J. D., Chu A. W.-H., Chan W.-M., Ng A. C.-K., Lee R., Fung K., Yeung A., Wu T.-C., Chan J. W.-M., Yan W.-W., Chan W.-M., Chan J. F.-W., Lie A. K.-W., Tsang O. T.-Y., Cheng V. C.-C., Que T.-L., Lau C.-S., Chan K.-H., To K. K.-W., Yuen K.-Y. (2020). Lancet.

[cit7] Yuan S., Yin X., Meng X., Chan J. F.-W., Ye Z.-W., Riva L., Pache L., Chan C. C.-Y., Lai P.-M., Chan C. C.-S., Poon V. K.-M., Lee A. C.-Y., Matsunaga N., Pu Y., Yuen C.-K., Cao J., Liang R., Tang K., Sheng L., Du Y., Xu W., Lau C.-Y., Sit K.-Y., Au W.-K., Wang R., Zhang Y.-Y., Tang Y.-D., Clausen T. M., Pihl J., Oh J., Sze K.-H., Zhang A. J., Chu H., Kok K.-H., Wang D., Cai X.-H., Esko J. D., Hung I. F.-N., Li R. A., Chen H., Sun H., Jin D.-Y., Sun R., Chanda S. K., Yuen K.-Y. (2021). Nature.

[cit8] Tanner J. A., Watt R. M., Chai Y.-B., Lu L.-Y., Lin M. C., Peiris J. S. M., Poon L. L. M., Kung H.-F., Huang J.-D. (2003). J. Biol. Chem..

[cit9] Ivanov K. A., Thiel V., Dobbe J. C., van der Meer Y., Snijder E. J., Ziebuhr J. (2004). J. Virol..

[cit10] Kwong A. D., Rao B. G., Jeang K. T. (2005). Nat. Rev. Drug Discovery.

[cit11] Lee C., Lee J. M., Lee N.-R., Jin B.-S., Jang K. J., Kim D.-E., Jeong Y.-J., Chong Y. (2009). Bioorg. Med. Chem. Lett..

[cit12] Kim M. K., Yu M.-S., Park H. R., Kim K. B., Lee C., Cho S. Y., Kang J., Yoon H., Kim D.-E., Choo H., Jeong Y.-J., Chong Y. (2011). Eur. J. Med. Chem..

[cit13] Adedeji A. O., Singh K., Calcaterra N. E., DeDiego M. L., Enjuanes L., Weiss S., Sarafianos S. G. (2012). Antimicrob. Agents Chemother..

[cit14] Yu M.-S., Lee J., Lee J. M., Kim Y., Chin Y.-W., Jee J.-G., Keum Y.-S., Jeong Y.-J. (2012). Bioorg. Med. Chem. Lett..

[cit15] Lee C., Lee J. M., Lee N.-R., Kim D.-E., Jeong Y.-J., Chong Y. (2009). Bioorg. Med. Chem. Lett..

[cit16] Yang N., Tanner J. A., Wang Z., Huang J.-D., Zheng B.-J., Zhu N., Sun H. (2007). Chem. Commun..

[cit17] Adedeji A. O., Singh K., Kassim A., Coleman C. M., Elliott R., Weiss S. R., Frieman M. B., Sarafianos S. G. (2014). Antimicrob. Agents Chemother..

[cit18] Tanner J. A., Zheng B.-J., Zhou J., Watt R. M., Jiang J.-Q., Wong K.-L., Lin Y.-P., Lu L.-Y., He M.-L., Kung H.-F., Kesel A. J., Huang J.-D. (2005). Chem. Biol..

[cit19] Cho J.-B., Lee J.-M., Ahn H.-C., Jeong Y.-J. (2015). J. Microbiol. Biotechnol..

[cit20] Hoffmann M., Eitner K., von Grotthuss M., Rychlewski L., Banachowicz E., Grabarkiewicz T., Szkoda T., Kolinski A. (2006). J. Comput.-Aided Mol. Des..

[cit21] Jia Z., Yan L., Ren Z., Wu L., Wang J., Guo J., Zheng L., Ming Z., Zhang L., Lou Z., Rao Z. (2019). Nucleic Acids Res..

[cit22] Yin W., Mao C., Luan X., Shen D.-D., Shen Q., Su H., Wang X., Zhou F., Zhao W., Gao M., Chang S., Xie Y.-C., Tian G., Jiang H.-W., Tao S.-C., Shen J., Jiang Y., Jiang H., Xu Y., Zhang S., Zhang Y., Xu H. E. (2020). Science.

[cit23] Peng Q., Peng R., Yuan B., Zhao J., Wang M., Wang X., Wang Q., Sun Y., Fan Z., Qi J., Gao G. F., Shi Y. (2020). Cell Rep..

[cit24] Yan L., Zhang Y., Ge J., Zheng L., Gao Y., Wang T., Jia Z., Wang H., Huang Y., Li M., Wang Q., Rao Z., Lou Z. (2020). Nat. Commun..

[cit25] Yin W., Luan X., Li Z., Zhou Z., Wang Q., Gao M., Wang X., Zhou F., Shi J., You E., Liu M., Wang Q., Jiang Y., Jiang H., Xiao G., Zhang L., Yu X., Zhang S., Eric Xu H. (2021). Nat. Struct. Mol. Biol..

[cit26] Kato K., Honma T., Fukuzawa K. (2020). J. Mol. Graphics Modell..

[cit27] Pérez-Moraga R., Forés-Martos J., Suay-García B., Duval J.-L., Falcó A., Climent J. (2021). Pharmaceutics.

[cit28] Jin Z., Du X., Xu Y., Deng Y., Liu M., Zhao Y., Zhang B., Li X., Zhang L., Peng C., Duan Y., Yu J., Wang L., Yang K., Liu F., Jiang R., Yang X., You T., Liu X., Yang X., Bai F., Liu H., Liu X., Guddat L. W., Xu W., Xiao G., Qin C., Shi Z., Jiang H., Rao Z., Yang H. (2020). Nature.

[cit29] Świderek K., Moliner V. (2020). Chem. Sci..

[cit30] Arafet K., Serrano-Aparicio N., Lodola A., Mulholland A. J., González F. V., Świderek K., Moliner V. (2021). Chem. Sci..

[cit31] Ramos-Guzmán C. A., Ruiz-Pernía J. J., Tuñón I. (2021). Chem. Sci..

[cit32] Jaffrelot Inizan T., Célerse F., Adjoua O., El Ahdab D., Jolly L.-H., Liu C., Ren P., Montes M., Lagarde N., Lagardère L., Monmarché P., Piquemal J.-P. (2021). Chem. Sci..

[cit33] Chan S.-W. (2020). Front. Microbiol..

[cit34] McKee D. L., Sternberg A., Stange U., Laufer S., Naujokat C. (2020). Pharmacol. Res..

[cit35] Freidel M. R., Armen R. S. (2021). PLoS One.

[cit36] Pokhrel R., Chapagain P., Siltberg-Liberles J. (2020). J. Med. Microbiol..

[cit37] Mirza M. U., Froeyen M. (2020). J. Pharm. Anal..

[cit38] Culletta G., Gulotta M. R., Perricone U., Zappalà M., Almerico A. M., Tutone M. (2020). Computation.

[cit39] Gurung A. B. (2020). Gene Reports.

[cit40] Kousar K., Majeed A., Yasmin F., Hussain W., Rasool N. (2020). BioMed Res. Int..

[cit41] Thurakkal L., Singh S., Roy R., Kar P., Sadhukhan S., Porel M. (2021). Chem. Phys. Lett..

[cit42] White M. A., Lin W., Cheng X. (2020). J. Phys. Chem. Lett..

[cit43] Konagurthu A. S., Whisstock J. C., Stuckey P. J., Lesk A. M. (2006). Proteins.

[cit44] Schrödinger L., The PyMOL Molecular Graphics System, Version 2.3, 2019

[cit45] Chakrabarti S., Jayachandran U., Bonneau F., Fiorini F., Basquin C., Domcke S., Le Hir H., Conti E. (2011). Mol. Cell.

[cit46] Law Y.-S., Utt A., Tan Y. B., Zheng J., Wang S., Chen M. W., Griffin P. R., Merits A., Luo D. (2019). Proc. Natl. Acad. Sci. U. S. A..

[cit47] Olsson M. H. M., Søndergaard C. R., Rostkowski M., Jensen J. H. (2011). J. Chem. Theory Comput..

[cit48] Søndergaard C. R., Olsson M. H. M., Rostkowski M., Jensen J. H. (2011). J. Chem. Theory Comput..

[cit49] Smith R. H. B., Dar A. C., Schlessinger A. (2019). bioRxiv.

[cit50] Stein S. A. M., Loccisano A. E., Firestine S. M. and Evanseck J. D., Principal Components Analysis: A Review of its Application on Molecular Dynamics Data, ed. D. C. Spellmeyer, Elsevier, 2006, vol. 2, pp. 233–261

[cit51] David C. C. and Jacobs D. J., in Protein Dynamics: Methods and Protocols, Springer, 2014, pp. 193–226

[cit52] Pedregosa F., Varoquaux G., Gramfort A., Michel V., Thirion B., Grisel O., Blondel M., Prettenhofer P., Weiss R., Dubourg V., Vanderplas J., Passos A., Cournapeau D., Brucher M., Perrot M., Duchesnay E. (2011). J. Mach. Learn. Res..

[cit53] Halko N., Martinsson P. G., Tropp J. A. (2011). SIAM Rev..

[cit54] McCammon J. A. (1984). Rep. Prog. Phys..

[cit55] Ichiye T., Karplus M. (1991). Proteins: Struct., Funct., Genet..

[cit56] Rosta E., Hummer G. (2015). J. Chem. Theory Comput..

[cit57] Hall T. M. T. (2005). Curr. Opin. Struct. Biol..

[cit58] Story R. M., Weber I. T., Steitz T. A. (1992). Nature.

[cit59] Bateman A., Martin M., Orchard S., Magrane M., Agivetova R., Ahmad S., Alpi E., Bowler-Barnett E., Britto R., Bursteinas B., Bye-A-Jee H., Coetzee R., Cukura A., Silva A., Denny P., Dogan T., Ebenezer T., Fan J., Castro L., Garmiri P., Georghiou G., Gonzales L., Hatton-Ellis E., Hussein A., Ignatchenko A., Insana G., Ishtiaq R., Jokinen P., Joshi V., Jyothi D., Lock A., Lopez R., Luciani A., Luo J., Lussi Y., MacDougall A., Madeira F., Mahmoudy M., Menchi M., Mishra A., Moulang K., Nightingale A., Oliveira C., Pundir S., Qi G., Raj S., Rice D., Lopez M., Saidi R., Sampson J., Sawford T., Speretta E., Turner E., Tyagi N., Vasudev P., Volynkin V., Warner K., Watkins X., Zaru R., Zellner H., Bridge A., Poux S., Redaschi N., Aimo L., Argoud-Puy G., Auchincloss A., Axelsen K., Bansal P., Baratin D., Blatter M., Bolleman J., Boutet E., Breuza L., Casals-Casas C., de Castro E., Echioukh K., Coudert E., Cuche B., Doche M., Dornevil D., Estreicher A., Famiglietti M., Feuermann M., Gasteiger E., Gehant S., Gerritsen V., Gos A., Gruaz-Gumowski N., Hinz U., Hulo C., Hyka-Nouspikel N., Jungo F., Keller G., Kerhornou A., Lara V., Le Mercier P., Lieberherr D., Lombardot T., Martin X., Masson P., Morgat A., Neto T., Paesano S., Pedruzzi I., Pilbout S., Pourcel L., Pozzato M., Pruess M., Rivoire C., Sigrist C., Sonesson K., Stutz A., Sundaram S., Tognolli M., Verbregue L., Wu C., Arighi C., Arminski L., Chen C., Chen Y., Garavelli J., Huang H., Laiho K., McGarvey P., Natale D., Ross K., Vinayaka C., Wang Q., Wang Y., Yeh L., Zhang J. (2021). Nucleic Acids Res..

[cit60] Katoh K., Standley D. M. (2013). Mol. Biol. Evol..

[cit61] Fu L., Niu B., Zhu Z., Wu S., Li W. (2012). Bioinformatics.

[cit62] Lu R., Zhao X., Li J., Niu P., Yang B., Wu H., Wang W., Song H., Huang B., Zhu N., Bi Y., Ma X., Zhan F., Wang L., Hu T., Zhou H., Hu Z., Zhou W., Zhao L., Chen J., Meng Y., Wang J., Lin Y., Yuan J., Xie Z., Ma J., Liu W. J., Wang D., Xu W., Holmes E. C., Gao G. F., Wu G., Chen W., Shi W., Tan W. (2020). Lancet.

[cit63] Briguglio I., Piras S., Corona P., Carta A. (2011). Int. J. Med. Chem..

[cit64] Yang X., Chen C., Tian H., Chi H., Mu Z., Zhang T., Yang K., Zhao Q., Liu X., Wang Z., Ji X., Yang H. (2018). FASEB J..

