# Supplementary material for: Modelling the active SARS-CoV-2 helicase complex as a basis for structure-based inhibitor design
Source: Chem Sci. 2021 Sep 6;12(40):13492–505. doi: 10.1039/d1sc02775a (PMC8528070; doi:10.1039/d1sc02775a)
Supplement: SC-012-D1SC02775A-s001 [file SC-012-D1SC02775A-s001.pdf]

Electronic Supplementary Material (ESI) for Chemical Science.
This journal is © The Royal Society of Chemistry 2021

Supporting Information for

Modelling the Active SARS-CoV-2 Helicase Complex as a Basis for Structure-based Inhibitor Design

Dénes Berta,[†, a, b] Magd Badaoui,[†,a, b] Sam Alexander Martino,[a, b] Pedro J. Buigues,[a, b] Andrei V. Pisliakov,*[c] Nadia Elghobashi-Meinhardt,*[d] Geoff Wells,*[e] Sarah A. Harris,*[f] Elisa Frezza,*[g] Edina Rosta*[a, b]

† Equal contributions

[a] Department of Physics and Astronomy, University College London; London WC1E 6BT, UK E-mail: e.rosta@ucl.ac.uk

[b] Department of Chemistry, King's College London; London SE1 1DB, UK

[c] Computational Biology, School of Life Sciences, University of Dundee, Dow Street, Dundee, DD1 5EH, UK E-mail: a.pisliakov@dundee.ac.uk

[d] Department of Chemistry, Technische Universität Berlin, 10623 Berlin, Germany E-mail: n.elghobashi-meinhardt@campus.tu-berlin.de

[e] UCL School of Pharmacy, University College London, 29/39 Brunswick Square, London WC1N 1AX, UK E-mail: g.wells@ucl.ac.uk

[f] School of Physics & Astronomy, University of Leeds, Leeds LS2 9JT, UK E-mail: s.a.harris@leeds.ac.uk

[g] Université de Paris, CiTCoM, CNRS, F-75006 Paris, France E-mail: elisa.frezza@parisdescartes.fr

Table of Contents

List of Homologous PDB Structures....3
Protonation State of Protein Residues....4
Supplementary Note 1: MD Simulations Details....5
List of MD Runs, Force Fields and Software. 7
RMSD....8
UniProt Sequence Similarity....9
Supplementary Note 2: Redundancy in the Similar Sequences from UniProtKB....10
ATP Binding Distances....17
Supplementary Note 3: Puckering Analysis....18
Puckering Results....19
Principal Component Analysis (PCA)....21
DCC Maps....22
Supplementary Note 4: Apo Structures....22
Principal Protein Motion....24
Pocket Analysis....24
Supplementary Note 5: Correlation of Trajectory Features and Pockets....30
References....35

List of Homologous PDB Structures

Table S1: PDB list of PDB structured used in structural comparison. Sequence similarity and RMSD is based on the mustang alignment (see Homology Modelling in the main text).

PDB ID	Sequence Length	Number of Identical Residues	RMSD	Nucleotide	NA
2xzo	770	85	7.2	ADP+AlF_4^-	ssRNA
5mzn	825	84	12.9	ADP	-
6jim	668	56	10.5	ADP+AlF_3	ssRNA
4f93	1778	49	23.0	ATP	-
3o8d	823	44	20.3	ADP	-
3o8r	822	41	20.6	ADP+BeF_3	ssRNA
3rrm	687	35	16.2	ADP	-
3i61	748	32	17.6	ADP+BeF_3	ssRNA
4tyw	762	32	19.8	ADP+BeF_3	ssRNA
6c90	968	32	18.8	ADP	-
3kqu	709	31	18.6	ADP+BeF_3	ssDNA
1xtj	689	30	13.3	ADP	-
3i62	750	29	17.4	ADP+AlF_4^-	ssRNA
4tz0	753	29	18.0	GDP+BeF_3	ssRNA
2whx	797	29	26.2	ADP	-
5e4f	722	28	19.5	ADP+AlF_4^-	-
6uv3	711	28	17.0	ADP+BeF_3	ssRNA
3kx2	985	28	21.9	ADP	-
6uv2	715	27	16.1	ADP+BeF_3	ssRNA
6uv4	705	27	17.3	ADP+BeF_3	ssRNA
5xdr	952	27	21.3	ADP	-
5y6m	691	26	21.1	ADP+AlF_3	-
3kql	704	26	20.2	ADP+AlF_3	ssDNA
2jlr	723	26	18.5	ANP	-
6adx	695	25	21.3	ADP	-
6uv1	706	25	17.8	ADP+BeF_3	ssRNA
3kqn	711	25	18.3	ADP+BeF_3	ssDNA
5k8u	712	25	19.5	ADP	-
5vhc	1059	25	23.2	ADP+BeF_3	-
2jls	723	24	20.2	ADP	-
5e3h	889	24	26.0	ADP+BeF_3	dsRNA
2jlv	706	23	20.2	ANP	ssRNA
6ady	695	23	21.6	ADP	-
2jlx	712	23	19.9	ADP+VO_4^{3-}	ssRNA
2pl3	653	22	18.0	ADP	-
2jlz	721	22	18.2	ADP	ssRNA
4ljy	738	22	27.8	ADP	-
5y6n	704	21	19.3	ADP	-
5y4z	714	21	20.0	ANP	-
3dkp	650	20	18.9	ADP	-
3wrx	711	18	23.3	AGS	-
3ex7	619	15	17.7	ADP+AlF_3	ssRNA
5sup	685	14	21.1	ADP+BeF_3	ssRNA

Protonation State of Protein Residues

The protonation state of aspartate and glutamate residues are all deprotonated, lysine and arginine residues are protonated. Corresponding PROPKA estimates are shown in Table S2.

Table S2. pKa estimates (in order of decreasing acidity) of aspartate, glutamate, lysine and arginine residues.

Asp	pKa	Glu	pKa	Lys	pKa	Arg	pKa
458	2.27	341	1.9	202	9.16	567	9.14
328	2.46	540	2.16	320	9.36	15	11.27
113	2.63	143	2.72	40	10.24	332	11.49
435	2.75	201	3.29	465	10.26	560	11.57
580	2.76	244	3.41	131	10.28	303	11.67
578	2.84	197	3.55	94	10.28	339	11.7
59	2.93	261	3.55	171	10.29	129	11.91
223	3.3	142	3.75	508	10.35	409	11.93
466	3.51	551	3.76	477	10.36	507	12.06
32	3.64	498	4.18	347	10.36	442	12.12
450	3.82	128	4.3	271	10.36	427	12.13
119	3.83	375	4.34	473	10.37	390	12.14
160	3.85	365	4.4	218	10.38	22	12.29
483	3.85	156	4.52	189	10.43	490	12.29
101	3.89	168	4.53	288	10.44	248	12.34
344	3.91	353	4.56	524	10.45	155	12.38
56	3.96	418	4.58	345	10.45	497	12.38
260	3.96	591	4.58	76	10.46	173	12.42
534	3.98	420	4.71	467	10.47	594	12.47
204	3.98	447	4.73	414	10.47	392	12.47
583	3.98	136	5.07	462	10.53	502	12.65
207	4.01	162	5.22	73	10.54	178	12.69
542	4.06	319	5.96	430	10.55	443	12.86
369	4.19			569	10.59	21	12.9
105	4.2			394	10.81	595	13.17
315	4.45			192	10.82	579	13.19
383	4.61			460	11.43	161	13.47
401	5.11			323	11.44	337	13.89
374	6.2			146	11.47	186	14.06
				276	11.49	212	14.15
				329	11.49		
				28	11.67		
				139	12.54		
				584	12.63		

The structure does not contain any disulphide bond thus cysteines are protonated, except the ones involved in zinc coordination (residues 5, 8, 16, 19, 26, 29, 50, 55 and 72). All histidines

are single protonated, their protonation state was assigned according to their hydrogen-binding network as detailed in Table S3.

Table S3. Histidine residues and their protonation state (HSD: delta protonated, HSE epsilon protonated), with reasoning based on the environment of the sidechains.

Residue	Protonation state	coordination
33	HSD	N_ε coordinates Zn^{2+}
39	HSE	N_δ coordinates Zn^{2+}
75	HSE	N_δ coordinates Zn^{2+}
164	HSD	H_δ donates hydrogen-bond to Asp207 backbone
230	HSD	H_δ donates hydrogen-bond to Ura7 backbone
245	HSD	
290	HSE	N_δ accepts hydrogen-bond from ATP ribose
311	HSE	N_δ accepts hydrogen-bond from Arg332<br>H_ε donates hydrogen-bond to Ura6 backbone
395	HSD	H_δ donates hydrogen-bond to Gln275 backbone
464	HSD	
482	HSD	N_ε accepts hydrogen-bond from Thr552
554	HSD	

Supplementary Note 1: MD Simulations Details

CHARMM/NAMD

The helicase model was used as starting point for MD simulations. The system consists of the helicase, three Zn^{2+} ions, ATP, Mg^{2+} and ssRNA with eight uracil bases. The MD simulations were performed using NAMD 2.13,[1] using CHARMM36 force field.[2] The system was solvated by 50,000 – 70,000 TIP3P water molecules resulting in a box of 120 Å per side. To neutralize the system and account for a 0.15 M KCl solution we added 171 K^+ and 189 Cl^- ions.[3] Periodic boundary conditions (PBC) were used in all the simulations and the particle mesh Ewald (PME) method was used for long-range electrostatic interactions. SHAKE algorithm was deployed to constraint the covalent bonds involving hydrogen atoms. A cutoff 12 Å was used to treat non-bonding interactions. The energy of the system was minimized using a standard protocol via steepest descent algorithm for a total number of 10,000 steps, followed by 50 ns equilibration with restrained heavy atoms (heavy atom of the backbone of the protein and the nucleic acid with an isotropic force of 1000 $kJmol^{-1}nm^{-1}$) in constant pressure and temperature (NPT) and constant volume and temperature (NVT; up to 1ns) at 303.15 K via standard MD procedure with a time step of 2 fs. To maintain the tetrahedral coordination of the three zinc ions in the ZBD domain, we applied a combination of angle and distance restrain during all the MD simulations. To help equilibrate the complexes, we used a harmonic constraint on selected contacts with a force constant of 10 kcal/mol for 15 ns in our preliminary MD simulations to maintain relevant contacts. These constraints were subsequently progressively reduced and removed during the next 20 ns, using the colvar function implemented in NAMD.

Amber/Gromacs

To compare simulation results obtained with MD, we also carried out MD simulations using GROMACS 2018[4–7] with the Amber ff99+ parmbsc0+chioL3 force field[8,9] for ssRNA and Amber14SB[10] for the helicase. To maintain the coordination of the Zn^{2+} ions, the ZAFF model was used[11]. The molecular systems were placed in a cubic box and solvated with TIP3P water molecules.[3] The distance between the solute and the box was set to at least 14 Å. The solute was neutralized with potassium cations and then $K^{+}Cl^{-}$ ion pairs were added to reach the salt concentration of 0.15 M. We used the ion corrections of Joung et al.[12] as this force field has been shown to produce stable RNA structures.[13] The parameters for Mg^{2+} are taken from Ref. [14]. Long-range electrostatic interactions were treated using the particle mesh Ewald method[15,16] with a real-space cut-off of 10 Å. The hydrogen bond lengths were restrained using P-LINCS,[5,17] allowing a time step of 2 fs.[18] Translational movement of the solute was removed every 1000 steps to avoid any kinetic energy build-up.[19] After energy minimization of the solvent and equilibration of the solvated system for 10 ns using a Berendsen thermostat (τ_T = 1 ps) and Berendsen pressure coupling (τ_P = 1 ps),[18] simulations were carried out in an NTP ensemble at a temperature of 300 K and a pressure of 1 bar using a Bussi velocity-rescaling thermostat[20] (τ_P = 1 ps) and a Parrinello-Rahman barostat (τ_P = 1 ps).[21] During minimization and heating, all the heavy atoms of the solute were kept fixed using positional restraints. The restraints on the RNA and the protein backbone were relaxed slowly during the equilibration from 1000 $kJmol^{-1}$ nm^2 to 10 $kJmol^{-1}\cdot nm^2$.

Amber/Amber-GPU

Additional MD simulations, constructed using the AmberTools20 building package, were performed with the GPU version of Amber18 using the ff14SB force field to represent the protein,[10] the ff99OL3 force field for the RNA,[8,9] ATP parameters from Meagher et al[22] and parameters for Mg^{2+} are taken from Ref. [23]. The tetrahedral coordination state of the zinc was maintained using the ZAFF bonded force field.[23] Note that additional parameters were required for the HIS-33 that interacted with the zinc via its epsilon nitrogen by reference to comparable parameters in the ZAFF using a hybrid of the center ID 4 and 6 models.[24] For structures where ATP is bound, the octahedral coordination of the Mg^{2+} (which involves bonds to the ATP β and γ phosphate oxygen atoms, one with oxygen of the Ser289 hydroxyl group and three structural water molecules) was constructed using the Chimera metal center builder.[25] The solute was neutralized with potassium cations, then the protein was immersed in a box of TIP3P water molecules extending a minimum of 10 Å from the protein surface, and $K^{+}Cl^{-}$ ion pairs were added to achieve a salt concentration of 0.14 M. MD simulations were performed in the NTP ensemble, with Berendsen temperature and pressure coupling. SHAKE was applied to all bonds involving hydrogen, allowing an MD integration timestep of 2 fs. Long-range electrostatic interactions were treated using the particle mesh Ewald method[15,16] with a real-space cut-off of 12 Å. To equilibrate the protein and nucleo-protein complexes, the systems was initially energy minimized with positional restraints placed upon the solute, followed by minimization of both solvent and solute. The system was then heated to 300 K in

the presence of positional restraints upon the solute, which were gradually reduced from 50 kcal/mol $Å^2$ to 1.0 kcal/mol $Å^2$ over a timescale of 100 ps. For the apo-helicase structure, all restraints were then removed. For the ATP-ssRNA helicase complex which included the coordinated Mg^{2+} ion, an additional 50 ns of equilibration was performed with harmonic distance restraints (set at 2.1 Å with a spring constant of 20 kcal/mol $Å^2$) to maintain the positions of coordinated atoms, and angle restraints imposing the octahedral geometry around the Mg^{2+} ion. An additional restraint was imposed to maintain the orientation of Asp374 and Glu375 to the adjacent coordinated water molecule, as observed in the MutS-ATP complex (PDB ID 1w7a[26]). Three 1 µs simulations of the apo-structure at a salt concentration of 140 mM, and one 1.5 µs simulation in neutralizing salt were performed. We have also obtained 1 µs simulations of the ATP-helicase (two replicas), the RNA-helicase and the ATP-ssRNA-helicase complex. For all coordinated ATP Mg^{2+} metal centers, these equilibration protocols provide stable octahedral geometries, including the complexed water molecules, during unrestrained MD over 1 µs timescales.

List of MD Runs, Force Fields and Software.

Table S4. Details of the molecular dynamic simulations ran.

	Name	Time	Force field	MD Software	Monomer/ Dimer
**apo**	Gromacs - Replica 1	1 µs	Amber[1]	Gromacs	Dimer
	Gromacs - Replica 2	1 µs	Amber[1]	Gromacs	Dimer
	Namd - Replica 1	1 µs	CHARMM36	Namd 2.13	Dimer
	Namd - Replica 2	1 µs	CHARMM36	Namd 2.13	Dimer
	Namd - Replica 3	1 µs	CHARMM36	Namd 2.13	Monomer
	Namd - Replica 4	1 µs	CHARMM36	Namd 2.13	Monomer
	Namd - Replica 5	1 µs	CHARMM36	Namd 2.13	Monomer
	Amber -Replica 1	1 µs	Amber[2]	Amber	Monomer
	Amber -Replica 2	1 µs	Amber[2]	Amber	Monomer
	Amber -Replica 3	1 µs	Amber[2]	Amber	Monomer
**holo**	Gromacs - Replica 1	1.5 µs	Amber[1]	Gromacs	Monomer
	Gromacs - Replica 2	1.5 µs	Amber[1]	Gromacs	Monomer
	Gromacs - Replica 3	500 ns	Amber[1]	Gromacs	Monomer
	Namd - Replica 1	1 µs	CHARMM36	Namd 2.13	Monomer
	Namd - Replica 2	1 µs	CHARMM36	Namd 2.13	Monomer
	Namd - Replica 3	1 µs	CHARMM36	Namd 2.13	Monomer
	Namd - Replica 4	1 µs	CHARMM36	Namd 2.13	Monomer
	Namd - Replica 5	1 µs	CHARMM36	Namd 2.13	Monomer
	Amber -Replica 1	1 µs	Amber[2]	Amber	Monomer

[1] Amber14SB for the protein - Amber ff99+ parmbsc0+chioL3 for the RNA

[2] Amber14SB for the protein – Amber ff99OL3 for the RNA

RMSD

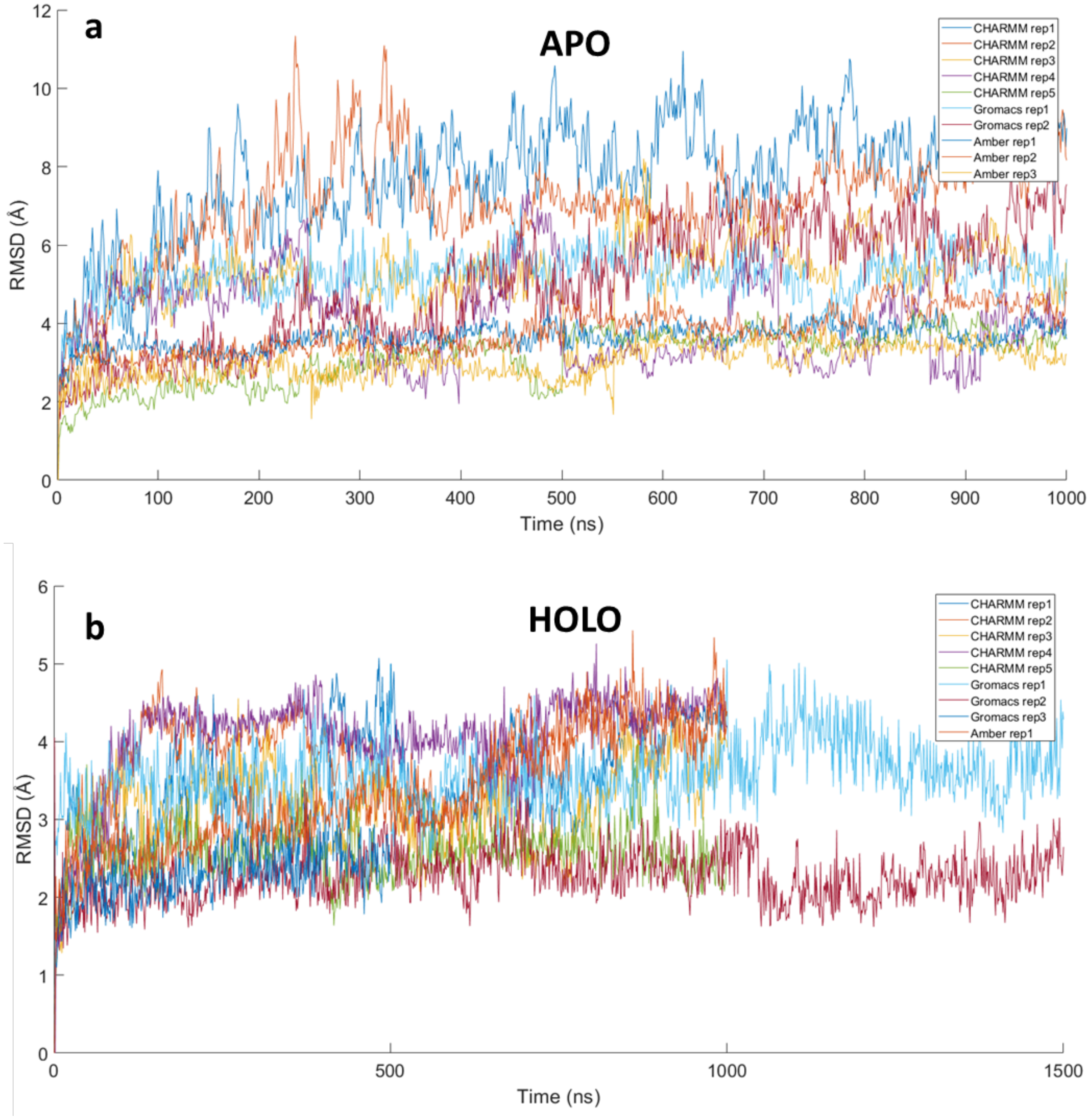

Figure S1. RMSD of the protein backbone along unbiased MD trajectories for the apo (**a**) and holo (**b**) models.

UniProt Sequence Similarity

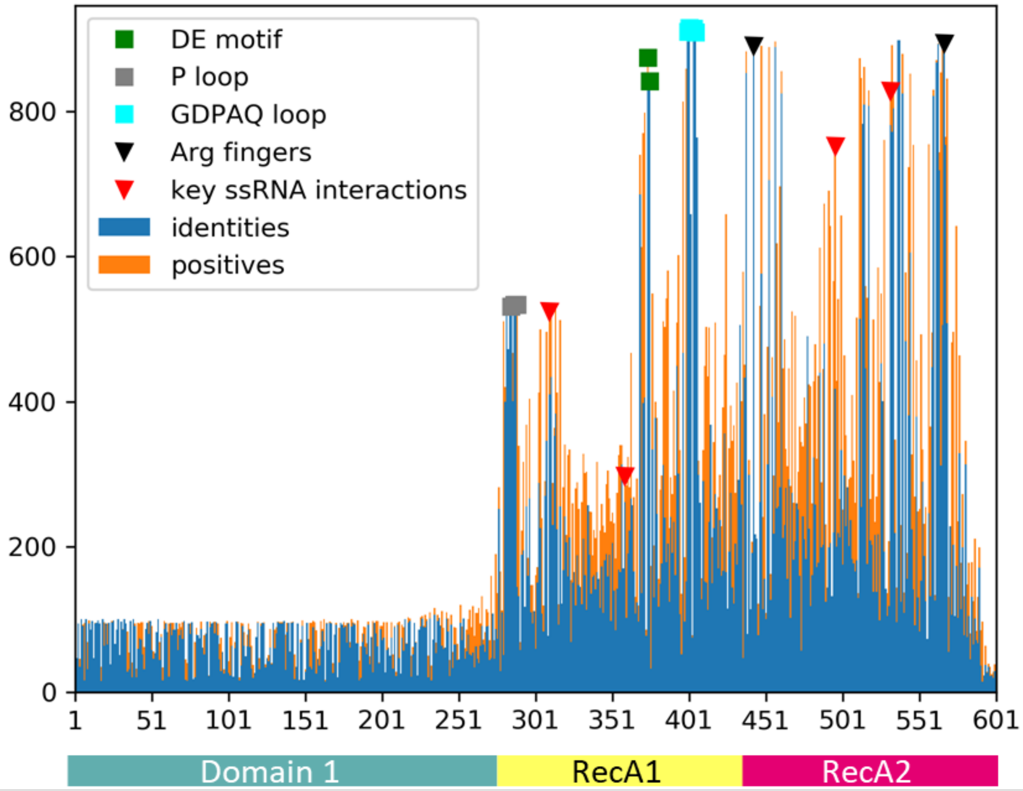

Figure S2. Sequence similarity of the sequences fetched from UniProtKB search aligned to the 601-residue long SARS-CoV-2 RNA helicase. Domain 1 shows similarity only to the close relatives (96 coronaviruses), while the RecA1 and RecA2 domains are more common across ATPase sequences. The pairwise alignments by UniProt do not recognize the first half of RecA1 (residues 270-350), by default.Key structural motifs are highlighted using symbols (P-loop: grey square, DE motif: green square, arginine fingers: black triangle, ssRNA interactions: red triangles).

Supplementary Note 2: Redundancy in the Similar Sequences from UniProtKB

The multipe sequence alignment produced for the representative 796 sequences is available here.

List of clusters determined by cd-hit, using 90% sequence identity as a cluster criterium. Sequences are identified by their UniProt ID

>Cluster 0
A0A1B6QLF6
A0A1D6GSE4
A0A1D6GSF6
A0A1D6GSF7
A0A1D6GSG0
A0A1D6GSG6
A0A1D6GSH0
A0A1D6GSH7
A0A1D6GSH9
A0A1D6KB56
A0A1D6KB59
A0A1D6KB66
A0A1D6KB68
A0A1D6KB81
>Cluster 1
A0A096XNP3
A0A0K1YZY7
A0A0U1WHG0
A0A0U1WHG8
B8Q8Q1
P0C6V9
P0C6W2
P0C6W6
P0C6X7
Q692E6
Q6UZF1
Q6UZF5
R9QTA1
>Cluster 2
A0A287NTV4
A0A287NTV8
A0A287NU34
A0A287NUL5
A0A3B6HX52
A0A3B6IRK6
A0A3B6ISC7
A0A446QZD2
A0A446QZI0
A0A446S183
A0A446S188
A0A446S1A0
>Cluster 3
A0A0B0MLF3
A0A0D2NIC2
A0A1U8JRD3
A0A2P5YLH7
A0A5B6WJY7
A0A5D2W139
A0A5D2W1E1
A0A5D3A3F8
>Cluster 4
A4ZTX2
A4ZU49
P0C6W7
P0C6W8
P0C6W9
P0C6X0
P0C6X6
>Cluster 5
A0A087R8F9
A0A091PRY0
A0A091SQJ3
A0A093BG16
A0A093F1K1
A0A093Q6I6
>Cluster 6
A0A125R5A3
C6GHC4
C6GHJ8
Q98VG9
V9PIT1
>Cluster 7
A0A3B6LP85
A0A3B6LPI6
A0A446UEE6
A0A446UEH8
A0A446UEJ4
>Cluster 8
A0A0L9UQ79
A0A1S3U570
A0A1S3U583
A0A3Q0EYP4
A0A3Q0F2N5
>Cluster 9
A0A095EZF8
A0A0T5P2F0
A0A2T7G1E9
A0A369TG04
A0A399J4N2
>Cluster 10
A0A0S2ZXR0
P0C6Y1
P0C6Y2
P0C6Y3
>Cluster 11
A0A0R0FTR0
A0A0R0FTS4
A0A0R0FU35
A0A0R0G4W7
>Cluster 12
A0A0D2V3N8
A0A0D2VWW4
A0A1U8NI48
A0A5D2VBC6
>Cluster 13
A0A446QZ97
A0A446QZ99
A0A446S195
M7ZRY7
>Cluster 14
A0A091H140
A0A091L8Q7
A0A091M2W0
A0A091P171
>Cluster 15
A0A1P8B6E0
B6SFA4
R0F313
V4LZ12
>Cluster 16
A0A453N5V2
A0A453N5W1
A0A453N730
A0A453N739
>Cluster 17
A3EXD8
A3EXE7
P0C6W4
>Cluster 18
I1TMH0
P0C6X9
P0C6Y0
>Cluster 19
A0A0U2LWJ9
H9TEX0
P0C6Y5
>Cluster 20
A0A3P9B9E6
A0A3Q3BZA6
I3J4V8
>Cluster 21
A0A446UEE1
A0A446UEG0
A0A446UEG3
>Cluster 22
A0A4Q4VLQ6
A0A4Q4YDI2
A0A4Q4YZM4
>Cluster 23
A0A1H3Q4V8
A0A1I5GPT8
A4FJR6
>Cluster 24
A0A1X7PJG2
A0A2V2LJ54
A8U4B0
>Cluster 25
A0A1H8TJ13
A0A1I3F1P9
A0A365TPT7
>Cluster 26
A0A3F2U1C2
A0A4R2GIQ6
A0A5C6TQ54
>Cluster 27
A0A1B1S8W4
A0A3N2N7G0
A0A4Q0IZW9
>Cluster 28
A0A0R0IIL5
A0A0R0IIN5
V7B556
>Cluster 29
A0A078IRV0
A0A0D3A7W4
M4EXG0
>Cluster 30
A0A0L9TB91
A0A1S3T8N6
A0A1S3T8P0
>Cluster 31
A0A2G5SB99
A0A2G5SBB2
A0A2G5SBC8
>Cluster 32
A0A222AID8
A0A222AIG8
>Cluster 33
A0A140H1G9
P0C6X2
>Cluster 34
P0C6W1
P0C6W3
>Cluster 35
C0KYW8
P0C6X8
>Cluster 36
P0DTD1
>Cluster 37
K9N7C7
T2B9U0
>Cluster 38
A0A0E3GHV1
A0A0E3J9G4
>Cluster 39
A3EXI1
A3EXI9
>Cluster 40
A0A0U1UZ20
B1PHK0
>Cluster 41
A0A096XNJ3
A0A1X9JPE2
>Cluster 42
A0A0U1UZ30
A0A0U1WHG4
>Cluster 43
H9EJ42
P0C6X5
>Cluster 44
A0A0U1WHF6
A8JNZ0
>Cluster 45
B2BW31

V5TFR4
>Cluster 46
A0A140ESF0
H9BQZ9
>Cluster 47
A0A1R1XUS5
A0A1R1Y4B2
>Cluster 48
V7BXW1
V7C1R1
>Cluster 49
A0A139HN52
A0A139I5B4
>Cluster 50
A0A1P8AT95
R0ILM7
>Cluster 51
V4KYN5
V4L5V5
>Cluster 52
R0LNH8
U3J0G9
>Cluster 53
D0MSE8
W2QDY2
>Cluster 54
A0A1D6KB54
A0A1D6KB69
>Cluster 55
A0A1D6GSJ5
A0A1D6KB88
>Cluster 56
A0A0R0JUN4
K7KYE7
>Cluster 57
E3MXP1
E3N3B6
>Cluster 58
A0A0N7F5X7
A0A1W2ECM8
>Cluster 59
E3NHZ1
E3NMA6
>Cluster 60
A0A182H3D0
A0A182H3D2
>Cluster 61
A0A222E4Z7
A0A2V4NJH9
>Cluster 62
A0A1Q4XVP1
A0A1V2QMD6
>Cluster 63
A7UR07
Q7QL33
>Cluster 64
A0A0X3TQ91
A0A1H5YI87
>Cluster 65
A0A3E0HQ86
W7SCC7
>Cluster 66
A0A2G5SCH3
A0A2G5UX19
>Cluster 67
M4S6T9
T0KAQ0
>Cluster 68
A0A1S3HNJ9
A0A1S3IUM2
>Cluster 69
A0A255SDT7
A0A255TAJ9
>Cluster 70
A0A444ZTB1
A0A445DMQ4
>Cluster 71
A0A059EJP1
A0A059F572
>Cluster 72
A0A0D3EHN6
M4CXZ8
>Cluster 73
A0A386JUR1
>Cluster 74
A0A345GNZ4
>Cluster 75
A0A076PY83
>Cluster 76
A0A1L3KIY4
>Cluster 77
A0A088DIE1
>Cluster 78
A0A023YA54
>Cluster 79
I6LMN0
>Cluster 80
P0C6X3
>Cluster 81
U5KNA9
>Cluster 82
A0A0A7UXR0
>Cluster 83
P0C6X4
>Cluster 84
H9AA60
>Cluster 85
E0XIZ2
>Cluster 86
B1PHI6
>Cluster 87
A0A1B3Q5W8
>Cluster 88
A0A0U1WHB2
>Cluster 89
P0C6W5
>Cluster 90
A0A166ZL34
>Cluster 91
Q008X6
>Cluster 92
S5YAF0
>Cluster 93
P0C6W0
>Cluster 94
K4K1U5
>Cluster 95
P0C6Y4
>Cluster 96
A0A1L2KGB4
>Cluster 97
P0C6X1
>Cluster 98
P0C6V8
>Cluster 99
U5IJ65
>Cluster 100
B3U1H4
>Cluster 101
Q9WPZ7
>Cluster 102
A0A0F6WGL5
>Cluster 103
D9J202
>Cluster 104
A0A0U1UZC3
>Cluster 105
A0A172B201
>Cluster 106
C7S857
>Cluster 107
A0A0U1WHG1
>Cluster 108
A0A240FW17
>Cluster 109
B6VDY6
>Cluster 110
B6VDX7
>Cluster 111
H9BR16
>Cluster 112
H9BR07
>Cluster 113
H9BR34
>Cluster 114
H9BR24
>Cluster 115
A0A1L3KIX8
>Cluster 116
A0A0F6PMZ2
>Cluster 117
H3EV11
>Cluster 118
Q55EG2
>Cluster 119
A0A1L3KJ46
>Cluster 120
A0A091DNS4
>Cluster 121
A0A1R0GY31
>Cluster 122
A0A3Q1FPH2
>Cluster 123
A0A1S3LB00
>Cluster 124
A0A2T9ZDP7
>Cluster 125
W1PPC2
>Cluster 126
G5AT46
>Cluster 127
S7Q2S4
>Cluster 128
A0A2K1K0G6
>Cluster 129
A0A0K6GGY7
>Cluster 130
A0A093INC5
>Cluster 131
A0A060WYY7
>Cluster 132
Q55F22
>Cluster 133
A0A3S3N3D6
>Cluster 134
W4XLQ4
>Cluster 135
E7FBJ2
>Cluster 136
A0A1U7EIW8
>Cluster 137
Q55F23
>Cluster 138
A0A5P1EQP0
>Cluster 139
A0A3P9AKK6
>Cluster 140
A0A553QYL0
>Cluster 141
A0A2R8Q0K5
>Cluster 142
A0A2P5EQ39
>Cluster 143
A0A1Y1VMB4
>Cluster 144
A0A0D3DWY3
>Cluster 145
J3MYT5
>Cluster 146
A0A4W5MRY2
>Cluster 147
A0A2I0AM03
>Cluster 148
A0A087HP32
>Cluster 149
A0A1P8AT74
>Cluster 150
C4R155
>Cluster 151
D7KEJ0
>Cluster 152
F4I5Z7
>Cluster 153
C5M7X3
>Cluster 154
A0A367YHY0
>Cluster 155
A0A397JD47
>Cluster 156
A0A4T0X5S3
>Cluster 157
A0A2D3UR11
>Cluster 158
D8SG52
>Cluster 159
A0A1Z5TCG9
>Cluster 160
A0A3N4I7P7
>Cluster 161
Q55F26
>Cluster 162
R7V241
>Cluster 163
A0A4V5N379
>Cluster 164
A0A2I0I2I1
>Cluster 165
A0A2I0RLA5
>Cluster 166
A0A2S6C3P4
>Cluster 167
A0A1V1T2X6
>Cluster 168
A0A4Z0YVE7
>Cluster 169

B4IWR8
>Cluster 170
A0A139H5D4
>Cluster 171
A0A4Y9Z278
>Cluster 172
A0A0P7VQT4
>Cluster 173
A0A439D1P7
>Cluster 174
Q54HF4
>Cluster 175
W7HXZ1
>Cluster 176
A0A0R0LZE5
>Cluster 177
A0A166RJB9
>Cluster 178
A0A2N1J8Y3
>Cluster 179
A0A3M6VIN3
>Cluster 180
W4XGP8
>Cluster 181
A0A0P1ATZ6
>Cluster 182
L2GUR3
>Cluster 183
A0A484E402
>Cluster 184
A0A078INI5
>Cluster 185
A0A4S4N2K7
>Cluster 186
A0A1W0WN61
>Cluster 187
A0A5D5AIX2
>Cluster 188
A5E4W0
>Cluster 189
A0A4Y9Y3D4
>Cluster 190
P38859
>Cluster 191
A0CR93
>Cluster 192
H3GXS9
>Cluster 193
A0A4Q2DT58
>Cluster 194
F0YDR4
>Cluster 195
N1Q8H0
>Cluster 196
A0C4S9
>Cluster 197
A0A4Y9Z0A5
>Cluster 198
A0A2A4JDD4
>Cluster 199
A0A3S3PBQ1
>Cluster 200
F6GVH0
>Cluster 201
A0A084G8N3
>Cluster 202
A0A074S5Z8
>Cluster 203
A0A1D8PPB3
>Cluster 204
A0A2D3VDH7
>Cluster 205
B4HIZ9
>Cluster 206
A0A2I0A5D1
>Cluster 207
A0A2H3ZQ23
>Cluster 208
A0A369S3F6
>Cluster 209
H8ZB33
>Cluster 210
H9JTJ3
>Cluster 211
A0A061FKT9
>Cluster 212
A0A067HBZ9
>Cluster 213
A0A0N1IHE3
>Cluster 214
A0A1Q8QU14
>Cluster 215
A0A314UX95
>Cluster 216
A0A4S4BFG4
>Cluster 217
D1BTZ4
>Cluster 218
A0A2G5SA58
>Cluster 219
A0A2A9D2V0
>Cluster 220
A0A068VHU1
>Cluster 221
R6B7X5
>Cluster 222
A0A4S5EUI9
>Cluster 223
A0A2H5Q001
>Cluster 224
A0A4S4DFR6
>Cluster 225
M0SXY4
>Cluster 226
A0A1R1PVC8
>Cluster 227
A0A200QWG4
>Cluster 228
A0A2P6TLG2
>Cluster 229
A0A0P7BVE6
>Cluster 230
A0A2Z4G9P8
>Cluster 231
A0A199W4N5
>Cluster 232
A0A2I2KQY1
>Cluster 233
A0A3P1VT07
>Cluster 234
A0A4Z0A839
>Cluster 235
Q2JCT1
>Cluster 236
T1IXE8
>Cluster 237
F8B5F2
>Cluster 238
A0A397I4G0
>Cluster 239
A0A151MIR9
>Cluster 240
A0A2A4JCZ8
>Cluster 241
A0A543NLB7
>Cluster 242
A0A1G6GWT3
>Cluster 243
A0A223S481
>Cluster 244
A0A318KP36
>Cluster 245
Q54HF3
>Cluster 246
A0A3N2DAW2
>Cluster 247
H0QQT2
>Cluster 248
A0A1X7DG02
>Cluster 249
D1BJG4
>Cluster 250
A0A0R0JNS6
>Cluster 251
A0A4P6EUF5
>Cluster 252
A0A4R6GB16
>Cluster 253
A0A1I2YZX8
>Cluster 254
A0A4C1XPL4
>Cluster 255
A0A1R3UKP3
>Cluster 256
A0A2S9QK70
>Cluster 257
A0A3R7SQB9
>Cluster 258
D9VEV4
>Cluster 259
A0A147EQP2
>Cluster 260
I3EIJ7
>Cluster 261
Q0RG49
>Cluster 262
A0A373DJS8
>Cluster 263
A0A2T0Y898
>Cluster 264
A0A409YFN5
>Cluster 265
A0A1S9MEF5
>Cluster 266
A0A1R4HN52
>Cluster 267
W4GCB2
>Cluster 268
A0A177EBR1
>Cluster 269
A0A2V5IV28
>Cluster 270
A0A4Y3ULD1
>Cluster 271
A0A1V2TFD5
>Cluster 272
U2XLE7
>Cluster 273
A0A0U5B9V5
>Cluster 274
A0A024U7B3
>Cluster 275
H6RC09
>Cluster 276
A0A0Q6R6R4
>Cluster 277
A0A3S9WD16
>Cluster 278
D6WDE8
>Cluster 279
A0A0M2HK28
>Cluster 280
A0A2W5Y4M1
>Cluster 281
A0A329YS09
>Cluster 282
A0A4P6KJ62
>Cluster 283
A0A4Q7M461
>Cluster 284
A0A0Q4GSZ1
>Cluster 285
A0A1R4F7V1
>Cluster 286
A0A367XSY0
>Cluster 287
A0A4P6Q5M3
>Cluster 288
A0A4R8WWP7
>Cluster 289
A0A0M2H1U4
>Cluster 290
A0A4Q7MCI8
>Cluster 291
W4GB70
>Cluster 292
A0A1M6AT99
>Cluster 293
D7AUM4
>Cluster 294
A0A1G8X9W1
>Cluster 295
A0A4R9AYS9
>Cluster 296
A0A1E8FNW3
>Cluster 297
T1L4Q6
>Cluster 298
A0A212FMP9
>Cluster 299
U6KZW2
>Cluster 300
A0A5B9G4W1
>Cluster 301
A0A2S6II10
>Cluster 302
A0A3N2FP80
>Cluster 303
A0A4Q8AAH8
>Cluster 304
A0A150HF54
>Cluster 305
A0A1H1B4X1
>Cluster 306
A0A260ZVV3

>Cluster 307
A0A2U1T0C2
>Cluster 308
A0A542FU32
>Cluster 309
A0A109QX71
>Cluster 310
I7MI99
>Cluster 311
A0A0T2L214
>Cluster 312
A0A1P8U5X7
>Cluster 313
A0A3G6ZPZ5
>Cluster 314
A0A4P8KQW2
>Cluster 315
A0A2T0SMC4
>Cluster 316
A0A2U1TCN8
>Cluster 317
A0A4P6FBG0
>Cluster 318
A0A5C8HTJ2
>Cluster 319
A0A165IGG6
>Cluster 320
A0A1G7WV85
>Cluster 321
A0A2A9DUS8
>Cluster 322
A0A4R8XRE0
>Cluster 323
A0A0B1ZWN7
>Cluster 324
A0A161SED6
>Cluster 325
A0A031FX04
>Cluster 326
A0A0Q8LU58
>Cluster 327
A0A172X9M2
>Cluster 328
A0A1R3VWG1
>Cluster 329
A0A2U0H5R5
>Cluster 330
E8NG09
>Cluster 331
A0A1D2N1G7
>Cluster 332
A0A4U3LPZ3
>Cluster 333
G7XFU5
>Cluster 334
A0A0F0LTQ8
>Cluster 335
A0A0Q5E5G5
>Cluster 336
A0A1D9DXS9
>Cluster 337
A0A2T0PTN8
>Cluster 338
A0A399G5L2
>Cluster 339
A0A2V1HS48
>Cluster 340
A0A2W1YI34
>Cluster 341
A0A498CLY6
>Cluster 342
E9T5P7
>Cluster 343
A0A0Q4FFM5
>Cluster 344
A0A3G8ZIR2
>Cluster 345
A0A077MF25
>Cluster 346
A0A1R4GXF1
>Cluster 347
A0A2Z2NHY5
>Cluster 348
A0A5B8M2P9
>Cluster 349
A0A371NS13
>Cluster 350
A0A5C8I0N3
>Cluster 351
A0A0Q8CT86
>Cluster 352
A0A0N7I497
>Cluster 353
A0A143Q6B7
>Cluster 354
A0A448ZGB5
>Cluster 355
A0A4Q2M921
>Cluster 356
A0A4V3WU53
>Cluster 357
A0A4Q2S8S8
>Cluster 358
A0A3L7A0E2
>Cluster 359
A0A132TBD7
>Cluster 360
I1IKN2
>Cluster 361
A0A099JJL3
>Cluster 362
A0A2M9BYI0
>Cluster 363
J7L810
>Cluster 364
A0A0Q4VC92
>Cluster 365
A0A173LXK1
>Cluster 366
A0A3S3MIS2
>Cluster 367
A0A4R2IZB1
>Cluster 368
A0A0T1W3U5
>Cluster 369
A0A1G8AIR4
>Cluster 370
A0A4Y8KR85
>Cluster 371
A0A0Q8UWZ0
>Cluster 372
A0A2S8WP82
>Cluster 373
A0A0Q5MAY0
>Cluster 374
A0A1G9RQK7
>Cluster 375
A0A418KV92
>Cluster 376
C6W953
>Cluster 377
A0A2A8HMX8
>Cluster 378
Q9CC95
>Cluster 379
A0A3A5MGT6
>Cluster 380
A1TDJ7
>Cluster 381
E1VT82
>Cluster 382
A0A0T0MM17
>Cluster 383
A0A3B6IPX0
>Cluster 384
A0A1G6Q1Q3
>Cluster 385
A0A495W5X8
>Cluster 386
U1L8E4
>Cluster 387
A0A495XMQ7
>Cluster 388
A0A098GC09
>Cluster 389
K0KAL8
>Cluster 390
A0A081NAR8
>Cluster 391
A0A561EKT9
>Cluster 392
A0A0Q7ZA01
>Cluster 393
A0A0S2JEU3
>Cluster 394
A0A151PFE5
>Cluster 395
A0A1E8Q5Z7
>Cluster 396
A0A1G8RIM6
>Cluster 397
A0A1Y4T5P0
>Cluster 398
A0A5C5ZBM5
>Cluster 399
I4BMU2
>Cluster 400
A0A0D0JTM0
>Cluster 401
A0A2S5W5U2
>Cluster 402
A0A3Q8WSQ0
>Cluster 403
A0A562IRS8
>Cluster 404
A0A5C8UTI1
>Cluster 405
Q73WX7
>Cluster 406
A0A084WF25
>Cluster 407
A0A1A0TRY3
>Cluster 408
A0A518ETA3
>Cluster 409
O50466
>Cluster 410
A0A0J6Z6I0
>Cluster 411
A0A1A1Y4U0
>Cluster 412
A0A1A3Q6C0
>Cluster 413
A0A257K5Z6
>Cluster 414
A0A2H2ILA1
>Cluster 415
B2HRG8
>Cluster 416
G5GE16
>Cluster 417
A0A1A2ZJV3
>Cluster 418
A0A1G6MLP5
>Cluster 419
G0FMD8
>Cluster 420
A0A1W1WHT0
>Cluster 421
A0A3S0X3I5
>Cluster 422
A0A4R5TYE8
>Cluster 423
A0A542LQR6
>Cluster 424
N2IWC2
>Cluster 425
A0A0W1ACQ1
>Cluster 426
A0A1A3N2S0
>Cluster 427
A0A380UJU6
>Cluster 428
A0A433JIH5
>Cluster 429
A0A4Q9GWL8
>Cluster 430
A0A3S0Y492
>Cluster 431
A0A0W0TM10
>Cluster 432
A0A1M5WPA9
>Cluster 433
W9ALK9
>Cluster 434
A0A0C1IND8
>Cluster 435
A0A0D6JJE4
>Cluster 436
A0A1H1KMJ9
>Cluster 437
A0A1W6ZUA3
>Cluster 438
R4SW20
>Cluster 439
A0A2P8EG31
>Cluster 440
A0A563UJD7
>Cluster 441
A0A0G3IQA0
>Cluster 442
A0A0K8R1V1
>Cluster 443
A0A1H8PWE6
>Cluster 444

A0A2A7N7H1
>Cluster 445
A0A344L589
>Cluster 446
A0A011MR13
>Cluster 447
A0A0W0XKZ6
>Cluster 448
E3NDG6
>Cluster 449
W5WE36
>Cluster 450
A0A1G6J395
>Cluster 451
A0A2U2J5A8
>Cluster 452
A0A558A1Y3
>Cluster 453
A0A1H9MW18
>Cluster 454
A0A4R5D9A0
>Cluster 455
A0A2A9FEP6
>Cluster 456
A0A328YKA8
>Cluster 457
A1T444
>Cluster 458
A0A1I1CFP7
>Cluster 459
A0A385BUB9
>Cluster 460
A4CHQ3
>Cluster 461
A0A0C1ERW9
>Cluster 462
A0A316EIT0
>Cluster 463
A0A4P7PV66
>Cluster 464
A0A4R6QFV4
>Cluster 465
A0A1H2VHH6
>Cluster 466
E3LI14
>Cluster 467
I3Z397
>Cluster 468
J9K2Q0
>Cluster 469
A0A1Q9LKG0
>Cluster 470
A0A1I3R123
>Cluster 471
A0A521DRM1
>Cluster 472
A0A3S4VH67
>Cluster 473
A0A0Q8XJT1
>Cluster 474
A0A2Z6AC58
>Cluster 475
A0A4Y8ZV66
>Cluster 476
W5JWN0
>Cluster 477
A0A182YDW8
>Cluster 478
T0PX45
>Cluster 479
A0A366ZPL7
>Cluster 480
A0A1R2C609
>Cluster 481
A0A3S0A4Q8
>Cluster 482
A0A5C8PDN6
>Cluster 483
A0A0Q7RRQ6
>Cluster 484
A0A2P8F2V5
>Cluster 485
W8S687
>Cluster 486
A0A081MBW1
>Cluster 487
A0A1J4KAY3
>Cluster 488
A0A1Y5TZZ9
>Cluster 489
A0A1A3JDV5
>Cluster 490
A0A367AH55
>Cluster 491
C7LS07
>Cluster 492
A0A0K1JKL8
>Cluster 493
A0A366E4I1
>Cluster 494
Q17Q00
>Cluster 495
V4RT94
>Cluster 496
A0A4Y9P7P8
>Cluster 497
A0A2P7S562
>Cluster 498
A0A4S3M4I8
>Cluster 499
A0A2S8RWB4
>Cluster 500
E3NBV2
>Cluster 501
A0A239Q172
>Cluster 502
A0A563ETS6
>Cluster 503
B0W0R8
>Cluster 504
A0A067CPM2
>Cluster 505
A0A1H8WLT9
>Cluster 506
A0A1I4H9I0
>Cluster 507
A0A285EF53
>Cluster 508
A0A291M0H5
>Cluster 509
A0A4Q1BUX3
>Cluster 510
D2SCF3
>Cluster 511
A0A4R7HWS3
>Cluster 512
A0A4U7MSG9
>Cluster 513
A0A1R2B4E2
>Cluster 514
A8NZS6
>Cluster 515
A0A0B7FUG6
>Cluster 516
A0A194Q0H0
>Cluster 517
W0BAQ7
>Cluster 518
A0A1Y2EQQ4
>Cluster 519
F5Z2K4
>Cluster 520
A0CYY6
>Cluster 521
A0A0K6GHC8
>Cluster 522
K8E8V0
>Cluster 523
U2EHH2
>Cluster 524
A2FI53
>Cluster 525
A0A558AVP5
>Cluster 526
A0A0B7NN86
>Cluster 527
D8R5Y4
>Cluster 528
A0A0M0JK10
>Cluster 529
A0A0G4GKS4
>Cluster 530
A8LA49
>Cluster 531
A0A416EQP8
>Cluster 532
V2Y199
>Cluster 533
A0A2K2TSG0
>Cluster 534
A0A1T4XIQ6
>Cluster 535
A0A1Y4T9D8
>Cluster 536
A0A4C1XUP2
>Cluster 537
A0A417U940
>Cluster 538
C4Z3E6
>Cluster 539
A0A0F5Q6E8
>Cluster 540
A0A0Q7IAI2
>Cluster 541
A0A1I0NRU6
>Cluster 542
K3W9Z7
>Cluster 543
A0A085F3L1
>Cluster 544
A0A1I7E5I2
>Cluster 545
A0A5B8MBU6
>Cluster 546
A0A0C9WZU6
>Cluster 547
A0A023B1A7
>Cluster 548
A0A090N4Y9
>Cluster 549
A0A0M8XR93
>Cluster 550
A0A4V3XBL7
>Cluster 551
A0A2K1IXN6
>Cluster 552
A0A401IB64
>Cluster 553
R6NWJ1
>Cluster 554
A0A2H2I231
>Cluster 555
A0A2H3IZZ8
>Cluster 556
A0A0F0GQG9
>Cluster 557
A0A286U7M0
>Cluster 558
A0A1R3UH54
>Cluster 559
F0ZZK1
>Cluster 560
A0A4R0RM51
>Cluster 561
A0A2H2I1X1
>Cluster 562
U2E7S0
>Cluster 563
A0A174GAK1
>Cluster 564
A0A2G8S6V8
>Cluster 565
A0A2M8TE44
>Cluster 566
R6D656
>Cluster 567
A0A108TC32
>Cluster 568
G1KKD2
>Cluster 569
G1NEQ7
>Cluster 570
A0A0L6W8V8
>Cluster 571
C4LUR8
>Cluster 572
A0A374AF26
>Cluster 573
Q5V3H7
>Cluster 574
R6CLH9
>Cluster 575
R6HVS4
>Cluster 576
A0A1J0GGK3
>Cluster 577
A0A3N2N5G8
>Cluster 578
A0A4R3MLH9
>Cluster 579
C4M1M9
>Cluster 580
A0A1M6KCJ0
>Cluster 581
A0A133XRY6

>Cluster 582
A0A1I0A7R4
>Cluster 583
A0A2X4SEG2
>Cluster 584
A0A1E5G0U7
>Cluster 585
A0A410DWU9
>Cluster 586
A0A0U5H4S5
>Cluster 587
C0GD25
>Cluster 588
K6T4R8
>Cluster 589
Q5LG91
>Cluster 590
A0A1Q3DWK5
>Cluster 591
A8EU08
>Cluster 592
A0A1M2VVG9
>Cluster 593
A0A1M4V387
>Cluster 594
A0A4P8YP60
>Cluster 595
A0A4Q0XN96
>Cluster 596
C4M7F8
>Cluster 597
J9IRA6
>Cluster 598
A0A4S4LMD8
>Cluster 599
A0A2U0ZKA5
>Cluster 600
A0A0K9PQC9
>Cluster 601
L7JYK9
>Cluster 602
A0A196SAE2
>Cluster 603
A0A5J9UXN9
>Cluster 604
K2GAH7
>Cluster 605
I3EFM4
>Cluster 606
A0A078ACK2
>Cluster 607
A0A087SRM2
>Cluster 608
S5ZUB1
>Cluster 609
A0A562KW96
>Cluster 610
G4TPV9
>Cluster 611
A0A4S4LNI9
>Cluster 612
U2KIS3
>Cluster 613
A0A443SAN4
>Cluster 614
A0A151RZE2
>Cluster 615
A0A2G8SGS5
>Cluster 616
A0A409XWL7
>Cluster 617
A0A250XPW9
>Cluster 618
A0A0L9UP77
>Cluster 619
A0A4Q2DNF4
>Cluster 620
A0A2I0J213
>Cluster 621
R7K8F6
>Cluster 622
A0A383WIC9
>Cluster 623
A0A090N009
>Cluster 624
W4JZ55
>Cluster 625
A0A2P6VA64
>Cluster 626
A0A2U1QFN3
>Cluster 627
K0TEV7
>Cluster 628
A0A151TJW9
>Cluster 629
A0A4Q2DR03
>Cluster 630
A0A0C9T6S8
>Cluster 631
A0A087VAM7
>Cluster 632
A0A091JTR7
>Cluster 633
A0A094KKX9
>Cluster 634
A0A0A0AS33
>Cluster 635
F6I552
>Cluster 636
A0A091NNA0
>Cluster 637
C1MS04
>Cluster 638
A0A1Q3DG92
>Cluster 639
A0A059B7M1
>Cluster 640
A0A087FZJ3
>Cluster 641
G7JY95
>Cluster 642
A0A059LRX6
>Cluster 643
A0A061DZ93
>Cluster 644
A0A1S2Z266
>Cluster 645
A0A284RLR5
>Cluster 646
A8NTE0
>Cluster 647
Q7M992
>Cluster 648
A0A0D7B9N5
>Cluster 649
A0A328DGN7
>Cluster 650
L2GPK1
>Cluster 651
A0A369K2T1
>Cluster 652
A0A0K9QI17
>Cluster 653
A0A409WY60
>Cluster 654
A0A3D8J6V4
>Cluster 655
A0A409Y5N2
>Cluster 656
A0A090MDN7
>Cluster 657
D3B8R1
>Cluster 658
L8H8R0
>Cluster 659
A0A2R6R404
>Cluster 660
J4C931
>Cluster 661
A0A0L6X2Z2
>Cluster 662
A0A4R0RQZ0
>Cluster 663
U5CZ54
>Cluster 664
A0A4R0RQB6
>Cluster 665
A0A2V0P7C4
>Cluster 666
A0A284RLQ5
>Cluster 667
A0A4Y9YD41
>Cluster 668
H8ZBI0
>Cluster 669
A0A226NLR4
>Cluster 670
J4I9L9
>Cluster 671
A0A1U7LVS6
>Cluster 672
A0A2G8SHD2
>Cluster 673
A0A388TJR2
>Cluster 674
A0A4Y9YDN6
>Cluster 675
A0A0K8R036
>Cluster 676
A0A4Y9YFI3
>Cluster 677
A0A401H1V8
>Cluster 678
A0A137QMV7
>Cluster 679
A0A369K313
>Cluster 680
A0A4Q0J9H8
>Cluster 681
A0A284RLQ2
>Cluster 682
A0A409VMW6
>Cluster 683
A0A060SGT7
>Cluster 684
A0A2W4E805
>Cluster 685
A0A1J4JZK3
>Cluster 686
A0A1J7IE79
>Cluster 687
A0A2T7NT63
>Cluster 688
A0A444TRV6
>Cluster 689
A0A4X3PBD6
>Cluster 690
A0A1R1LFB6
>Cluster 691
A0A2G8SGU0
>Cluster 692
W4KDQ5
>Cluster 693
W0K5W0
>Cluster 694
A0CKK8
>Cluster 695
A0A0C3PK16
>Cluster 696
A0A1J8PYV1
>Cluster 697
A0A4S4LQK3
>Cluster 698
A0A137QMW3
>Cluster 699
A0A368FWS7
>Cluster 700
M5EKB6
>Cluster 701
A0A0H3XK56
>Cluster 702
W7IDP9
>Cluster 703
A0A3N4KMQ6
>Cluster 704
A0A553NBD1
>Cluster 705
I3EI44
>Cluster 706
H1HIN5
>Cluster 707
A0A409X7W1
>Cluster 708
H3F8B2
>Cluster 709
B0WR31
>Cluster 710
A0A076FFX0
>Cluster 711
A0A177ECQ9
>Cluster 712
A0A067MYT9
>Cluster 713
Q16VB7
>Cluster 714
A0A195DME3
>Cluster 715
A0A345UIY9
>Cluster 716
A0A2A2GBD7
>Cluster 717
C5DDG3
>Cluster 718
A0A5C5Z2U9
>Cluster 719

A0A165HY28
>Cluster 720
A0A2U2XCJ6
>Cluster 721
A0A5B9R602
>Cluster 722
A0A0C3F267
>Cluster 723
Q6LWX3
>Cluster 724
X7Y5T7
>Cluster 725
A0A1M5JD62
>Cluster 726
A0A518G5E4
>Cluster 727
F0SEP5
>Cluster 728
A0A444ZTC7
>Cluster 729
V6LSU3
>Cluster 730
A0A199VK37
>Cluster 731
C4V6S2
>Cluster 732
K7JWU9
>Cluster 733
A0A1I7BKI6
>Cluster 734
A0A1Y2RBK3
>Cluster 735
A0A2I0R4A0
>Cluster 736
A0A4Y9ZWX4
>Cluster 737
A5UPE6
>Cluster 738
A0A563U2Z6
>Cluster 739
A0A1H4GKN2
>Cluster 740
D3S311
>Cluster 741
A0A0Q5H458
>Cluster 742
A0A257K799
>Cluster 743
F2IEP2
>Cluster 744
A0A2P7T9L4
>Cluster 745
A0A519GZC1
>Cluster 746
A0A0N8GLX8
>Cluster 747
A0A0U1PWH0
>Cluster 748
A0A2U2HLZ1
>Cluster 749
A0A4Y7SQX7
>Cluster 750
A0A4S5BLU5
>Cluster 751
E8N3T7
>Cluster 752
A0A233RGQ2
>Cluster 753
A0A2M8H255
>Cluster 754
L2GL01
>Cluster 755
A0A150AGX0
>Cluster 756
A0A0C2GW16
>Cluster 757
Q8SVI3
>Cluster 758
A0A059C832
>Cluster 759
R7ZUK0
>Cluster 760
A0A0D7BAT7
>Cluster 761
F2KNT4
>Cluster 762
N0BDX2
>Cluster 763
L7JU07
>Cluster 764
S8DTU5
>Cluster 765
A0A075AUX8
>Cluster 766
A0A1Y4WES2
>Cluster 767
A0A3M7RTY0
>Cluster 768
A0A2G5BAP2
>Cluster 769
A0A166G1M4
>Cluster 770
X6M8C8
>Cluster 771
A0A482SHI2
>Cluster 772
A0A2H2IFQ8
>Cluster 773
O28319
>Cluster 774
S8CAX4
>Cluster 775
A0A371QRX3
>Cluster 776
A0A3P5VKV2
>Cluster 777
A0A0M0JR31
>Cluster 778
U2RPE4
>Cluster 779
Q09594
>Cluster 780
J9DT93
>Cluster 781
A0A5C3LMP8
>Cluster 782
D8M686
>Cluster 783
A0A437AN42
>Cluster 784
A0A260YMM2
>Cluster 785
A0A0N4VWY0
>Cluster 786
A0A1X6MRM5
>Cluster 787
A0A4S8MLD2
>Cluster 788
A0A4R0XRT5
>Cluster 789
A0A067M9A8
>Cluster 790
A0A067MMS4
>Cluster 791
A0A0C9U5Z5
>Cluster 792
A0A2U1KB04
>Cluster 793
F8PYC3
>Cluster 794
A0A2T4CG31
>Cluster 795
J9DLZ7

ATP Binding Distances

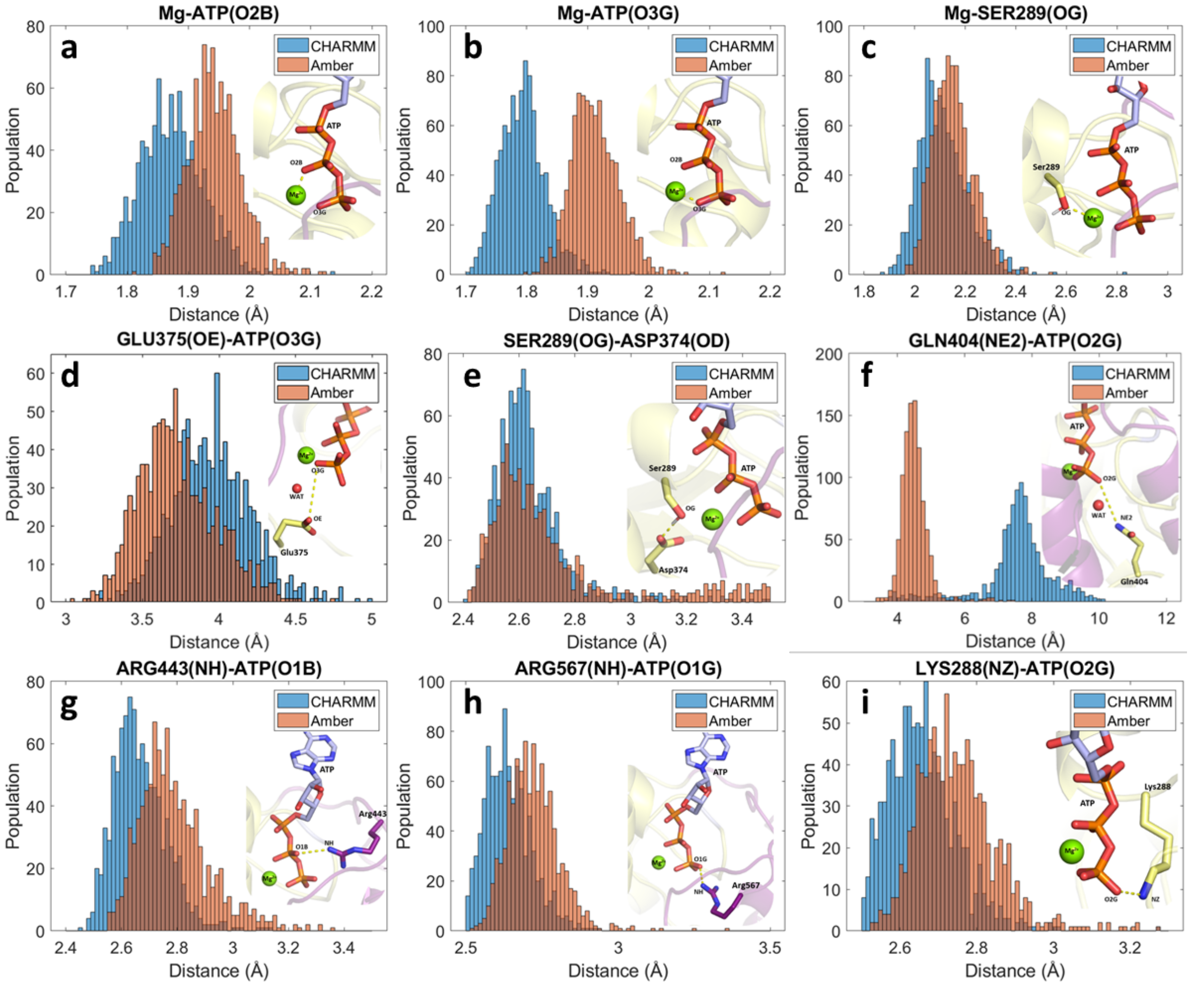

Figure S3. Histograms of nine key interactions in the ATP pocket has been monitored during the multiple MD simulations. For each distance (see title above each plot), we represent the distribution along multiple replicas for the CHARMM (blue) and Amber (orange) force field. The structural representation of each distance is shown in the insets.

Supplementary Note 3: Puckering Analysis

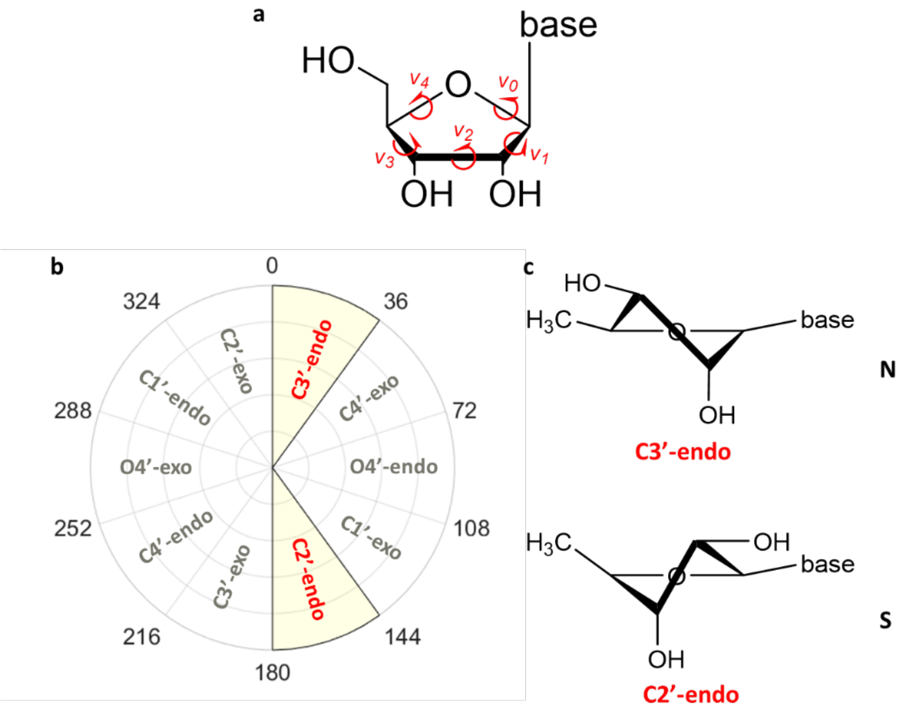

Figure S4. a: Dihedral angles involved in the conformational description. b: Envelope conformers of the ribose ring associated to puckering angle values. c: Schematic example conformers of C3'-endo and C2'-endo highlighting the out-of-plane atoms.

To take the ribose conformation into account, we described the sugar ring using pseudorotation parameters (Figure S4). Although there are four possible pseudorotation parameters for a five- membered ring,[27] two in particular are useful to characterize the sugar conformation: the phase (*Pha*) and the amplitude (*Amp*). While the amplitude describes the degree of ring puckering, the phase describes which atoms are most out of the mean ring plane. We calculated these parameters using the expression:[28]

$$\mathrm{Amp} = \sqrt{(\mathrm{a}^2 + \mathrm{b}^2)}\ ;\ \mathrm{Pha} = \cos^{-1}\left(\frac{\mathrm{a}}{\mathrm{Amp}}\right)$$

where $\mathrm{a} = 0.4\sum_{\mathrm{i}=1}^{5} \mathrm{v_i} \cos[0.8\pi(\mathrm{i}-1)]$ and $\mathrm{b} = -0.4\sum_{\mathrm{i}=1}^{5} \mathrm{v_i} \sin[0.8\pi(\mathrm{i}-1)]$, with v_i the ring dihedral angle *i*. This approach has the advantage of processing the five ring dihedrals from v_1 (C1-C2-C3-C4) to v_5 (O4-C1-C2-C3) in an equivalent manner. Conventionally, sugar ring puckers are divided into 10 families described by the atom which is most displaced from the mean ring plane (C1 , C2 , C3 , C4 or O4) and the direction of such displacement (endo for displacements on the side of the C5 atom and exo for displacements on the other side). Using the Curves+ program[29] for each simulation trajectory, for each nucleotide we computed the percentage of appearance for each family. To understand the interplay between the sugar conformation and the chemical reactivity, we grouped the sugar puckers into two large families. The sugar puckers C1'-exo, C2'-endo, C3'-exo, C4'-endo belong to the B-like family, while C1'- endo, C2'-exo, C3'-endo, C4'-exo belong to the A-like family.

Puckering Results

PDBID	2jlx	2jlz	2xzl	2xzo	3ex7	3i61	3i62	3o8r	4tyw	4tz0	5sup	6jim	6uv1	6uv2	6uv3	6uv4
nt 1			C3' endo	C3' endo		C2' endo	C2' endo		C3' endo	C3' endo		C2' endo				
nt 2	C3' endo	C3' endo	C2' endo	C2' endo	C1' endo	C3' endo	C3' endo	C2' endo	C3' endo	C3' endo	C3' endo	C2' endo	C3' endo	C3' endo	C3' endo	C3' endo
nt 3	C3' endo	C3' endo	C3' endo	C3' endo	C3' endo	C2' endo	C3' endo	C4' exo	C3' endo	C3' endo	C3' endo	C3' endo	C3' endo	C3' endo	C3' endo	C3' endo
nt 4	C3' endo	C3' endo	C3' endo	C3' endo	C2' exo	C3' endo	C3' endo	C3' endo	C3' endo	C3' endo	C3' endo	C4' exo	C3' endo	C3' endo	C3' endo	C3' endo
nt 5	C3' endo	C3' endo	C3' endo	C3' endo	C3' endo	C3' endo	C2' exo	C3' endo	C3' endo	C3' endo	C3' endo	C3' endo	C3' endo	C3' endo	C3' endo	C3' endo
nt 6	C1' exo	C3' endo	C2' endo	C4' exo	C3' endo	C3' endo	C3' endo	C3' endo	C3' endo	C3' endo	C3' endo	C3' endo	C3' endo	C3' endo	C3' endo	C3' endo
nt 7	C4' exo	C3' endo	C2' endo		O4' endo	C3' endo	C3' endo	C3' endo	C3' endo	C2' endo	C3' endo	C3' endo	C3' endo	C3' endo	C3' endo	C2' endo
nt 8	C3' endo	C3' endo	C2' endo			C3' endo	C3' endo						C3' exo	C3' endo		C2' endo
nt 9						C3' exo	C3' exo									
nt 10						C3' endo	C3' endo									

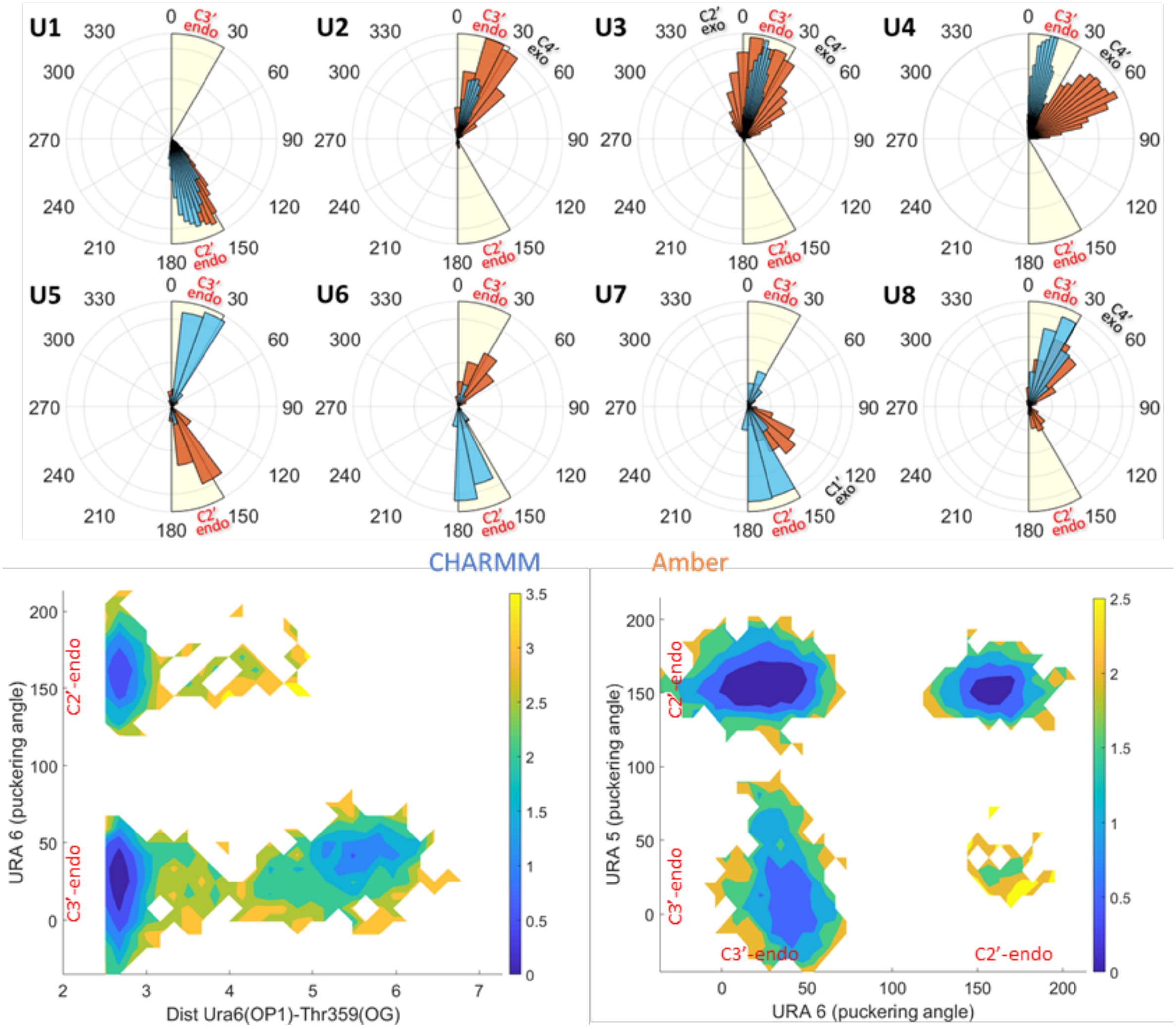

Figure S5: top PDB table of different RNA-helicase structures with the relative puckering values for each nucleotide in the RNA. center Circular histogram representing the puckering angle distribution for the eight nucleotides present in our MD simulations (CHARMM: blue and Amber: orange). bottom 2D energy surfaces of the puckering angle of uracil in position 6 of the RNA and the Ura6(OP1) – Thr359(OG) distance (left plot) and the puckering angles of Ura5 (y axis) and Ura6 (x axis).

RNA stability

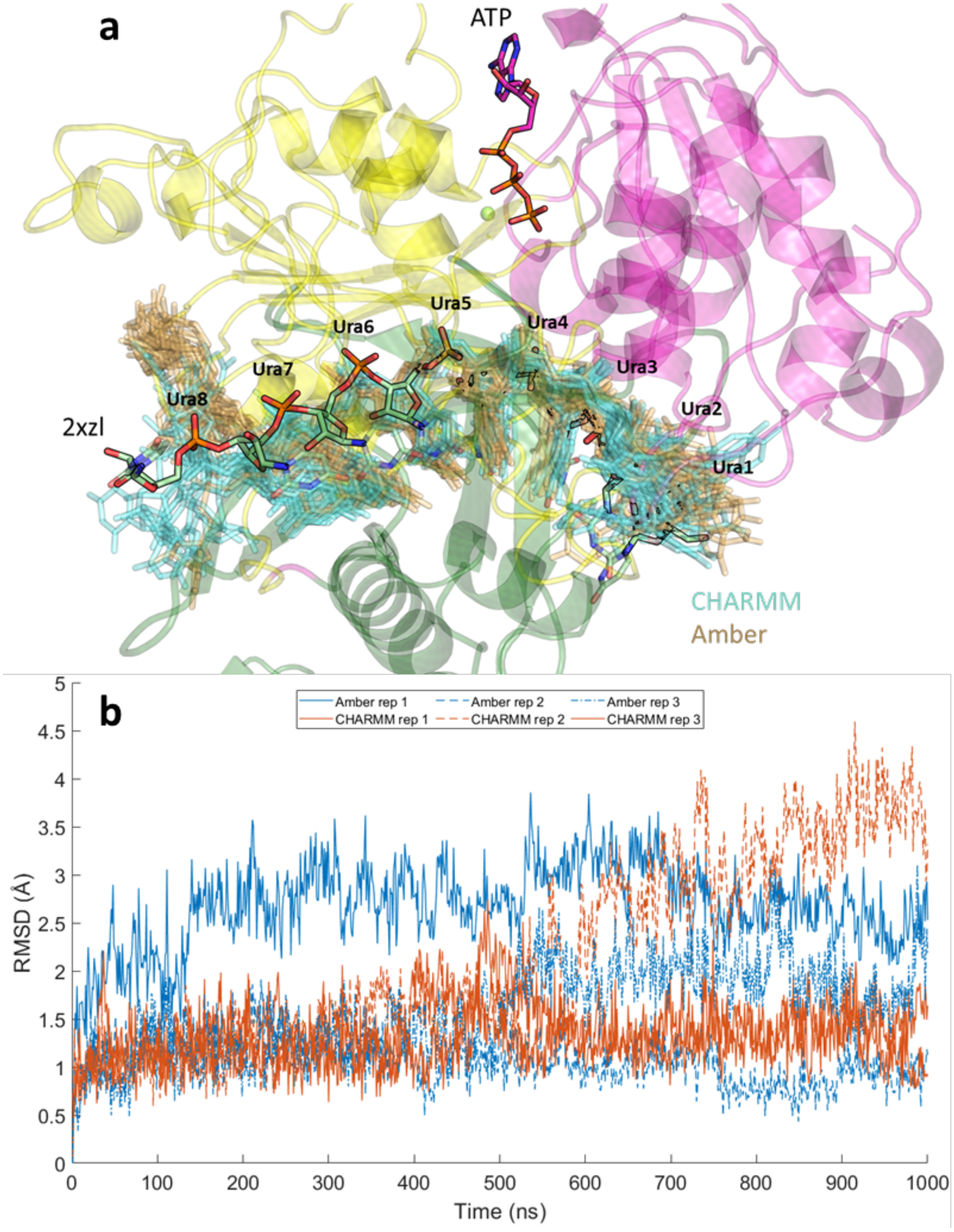

Figure S6. **a**: Structural comparison of the RNA chains using two different force fields (CHARMM: blue and Amber: orange), The ssRNA from 2xzl is shown in sticks with outline, as a reference. Excluding the terminal nucleotides (Ura1 and Ura8), the RNA is stable in the pocket and aligns well with 2xzl. **b** RMSD of the backbone of the ssRNA residues, excluding terminal uracils 1, 7 and 8.

Principal Component Analysis (PCA)

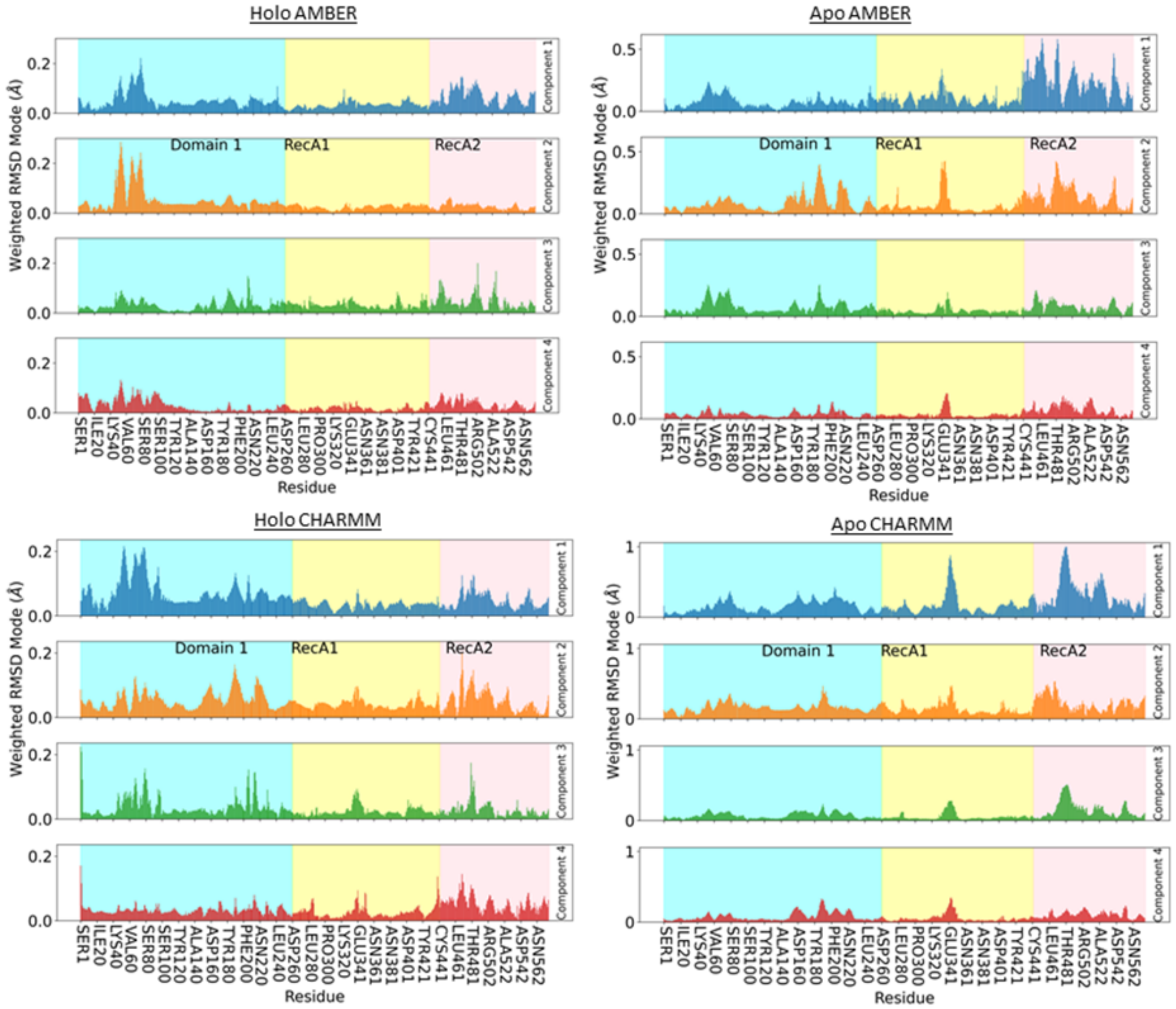

Figure S7. Weighted RMSD modes for the first four PCA components of each simulation type. A larger value indicates that residue contributes more to the motion described by the respective PCA component.

DCC Maps

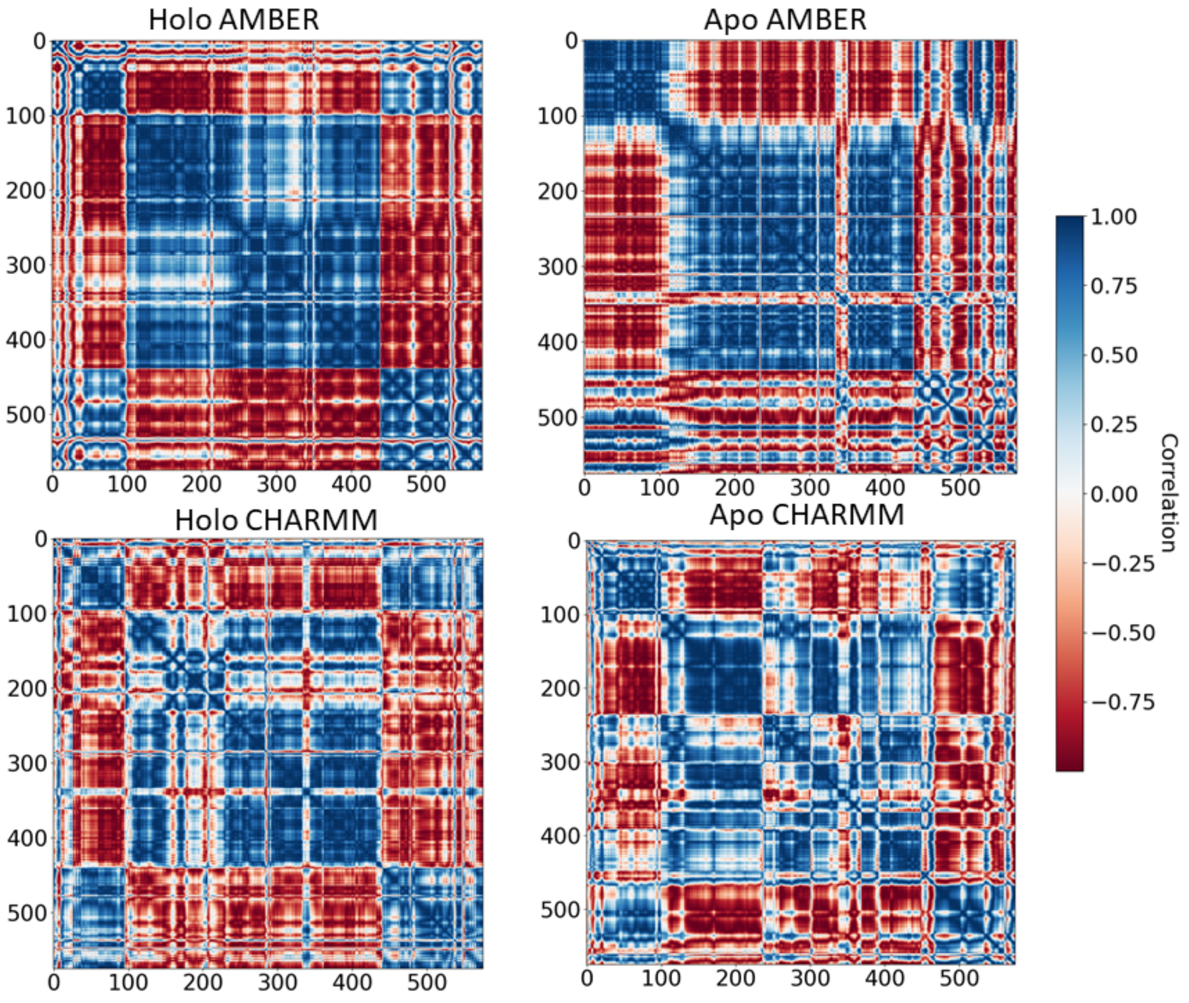

Figure S8. DCC maps showing the correlation between the interatomic displacements over the motion described by the first principal component.

Supplementary Note 4: Apo Structures

All replicas of the apo structure show low flexibility and no major changes of the backbone structure of the dimer. We analyze the overall flexibility of the dimers and compare our results with the experimental b-factor obtained in 6jyt and 6zsl (Figure S9 in the main text). Our model, in common with the two crystal structures, shows higher flexibility on the external shell of RecA2 domain, while the ATP and the RNA pockets appear to be more conserved. The ZBD shows low flexibility, in agreement with the b-value of 6jyt, but not with 6zsl (especially chain A), in which the temperature factor is higher, due to the different dimerization of the crystal structures (Figure S9).

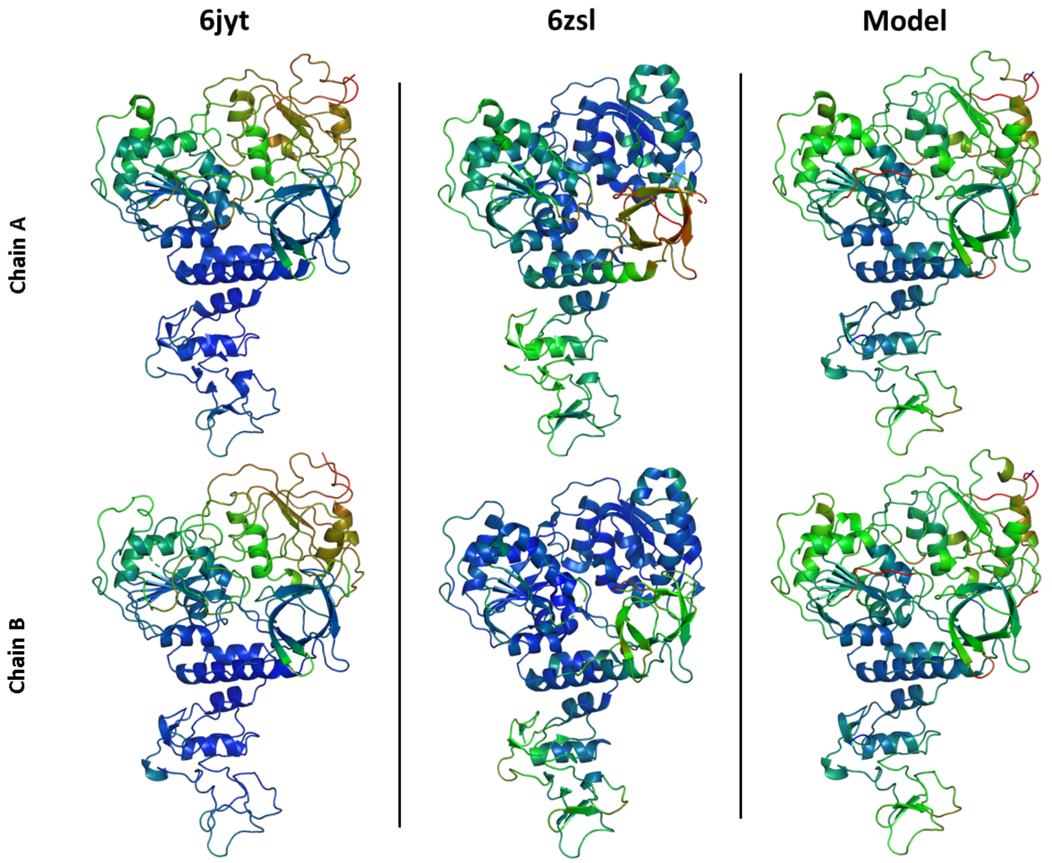

Figure S9. Conformational flexibility of the apo helicase protomers from 6jyt, 6zsl and our model if the apo dimer from the MD simulations. The residues are colored according to the deposited PDB B-factors (6jyt and 6zsl; from blue: low B-factor to red: high B-factor), and by the residue RMSD from the MD trajectory.

Principal Protein Motion

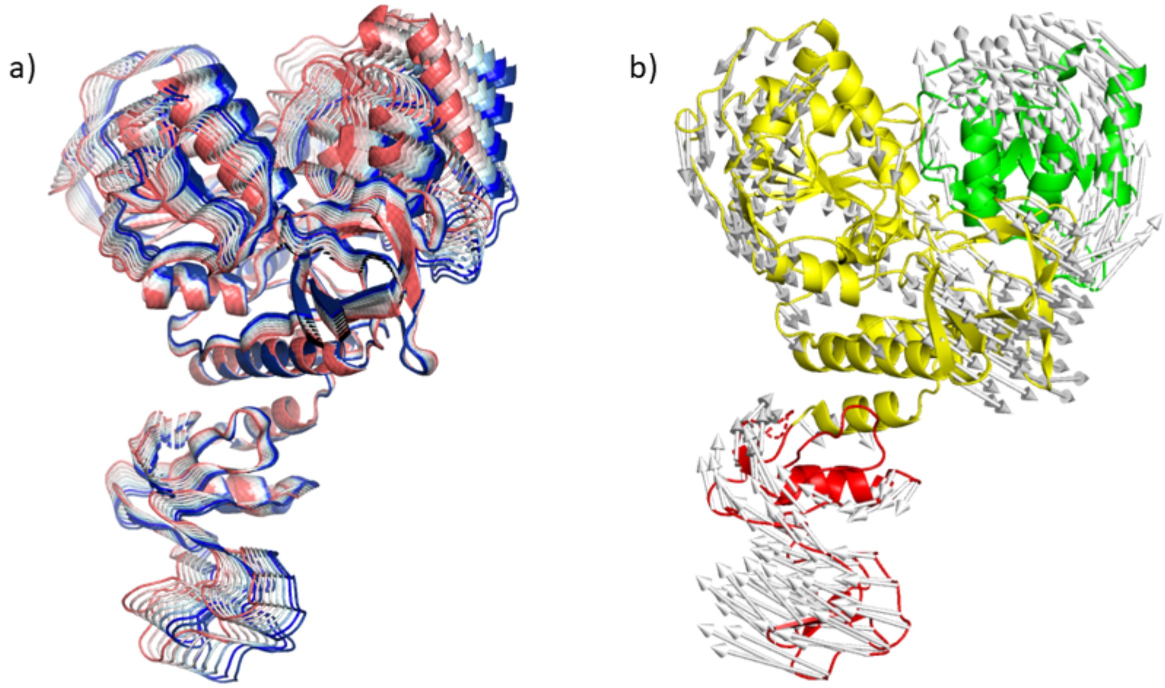

Figure S10. This shows the motion described by the first PCA component for holo simulations carried out in AMBER. a) Represents the motion using full structures, starting from the minimum value of the principal component observed (blue) to the maximum value (red). b) Represents the motion with displacement vectors. The cartoon is colored by regions defined in Figure 6e in the main text: red: (i) the ZBD domain, yellow: (ii) the rest of Domain 1 along with RecA1, and green: (iii) RecA2.

Pocket Analysis

Along the simulation trajectories, we define pocket distances to every 10th alpha carbons, and use these internal coordinates for identification. A representative pocket is selected from one frame and the similarity of other pockets from all frames are calculated via the pocket distances. A pocket is deemed identical to the reference, if it is below a threshold for the Cartesian distance within the space of the internal coordinates. This threshold is determined using the distribution of pocket distances. The analysis is implemented into our version of pyvol, and available together with the thresholds used via the link: https://github.com/bertadenes/pyvol."

Table S5. Extended statistics of pocket volumes in different simulations. Pockets are named as depicted in Figure 7 in the main text. Holo and apo refers to whether the ATP and RNA substrates were bound to the helicase in the simulation. CH and Am are CHARMM and Amber Force Fields (FFs).

	**system**	**FF**	**ATP pocket**	**RNA pocket**	**RecA2 pocket1**	**RecA2 pocket2**	**RecA2 pocket3**	**Stalk pocket**	**ZBD pocket**
**Presence (%)**	**holo monomer**	**CH**	96,28	98,60	18,45	22,25	54,57	26,52	16,91
		**Am**	98,28	98,18	34,92	18,12	-	16,40	20,26
	**apo monomer**	**Am**	81,40	99,74	35,06	-	37,00	17,77	7,89
	**apo dimer chain A**	**CH**	93,03	84,57	89,27	-	91,63	92,03	19,90
		**Am**	70,11	99,55	27,99	-	-	25,70	4,99
	**apo dimer chain B**	**CH**	99,17	99,17	72,17	94,17	70,73	36,00	-
		**Am**	69,81	99,70	42,96	-	-	22,46	5,04
**Volume / Å^3**	**holo monomer**	**CH**	630±263	1472±858	276±64	409±179	405±200	449±202	268±74
		**Am**	542±223	1911±565	330±91	353±119	**-**	300±75	246±47
	**apo monomer**	**Am**	665±338	2415±873	303±106	-	286±99	282±73	247±50
	**apo dimer chain A**	**CH**	695±473	2889±1025	477±327	-	451±305	693±468	256±66
		**Am**	699±287	3035±968	312±103	-	-	282±68	253±62
	**apo dimer chain B**	**CH**	1657±1507	2411±1564	356±149	392±185	432±200	329±96	-
		**Am**	746±374	3127±1056	338±127	-	-	285±76	244±41

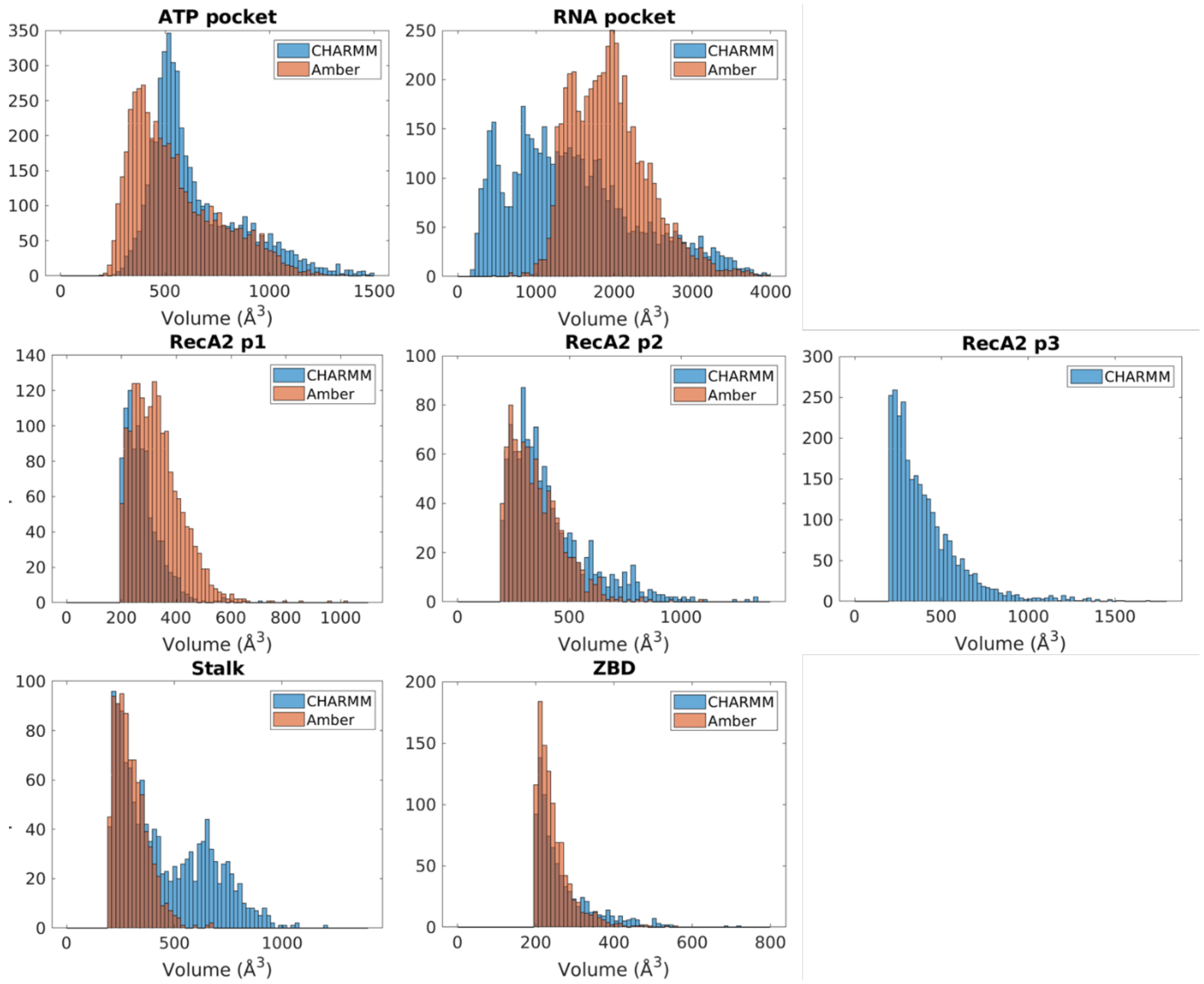

Figure S11. Distribution of pockets grouped by force fields, data collected from holo simulations. Pockets are named as depicted in Figure 7 in the main text.

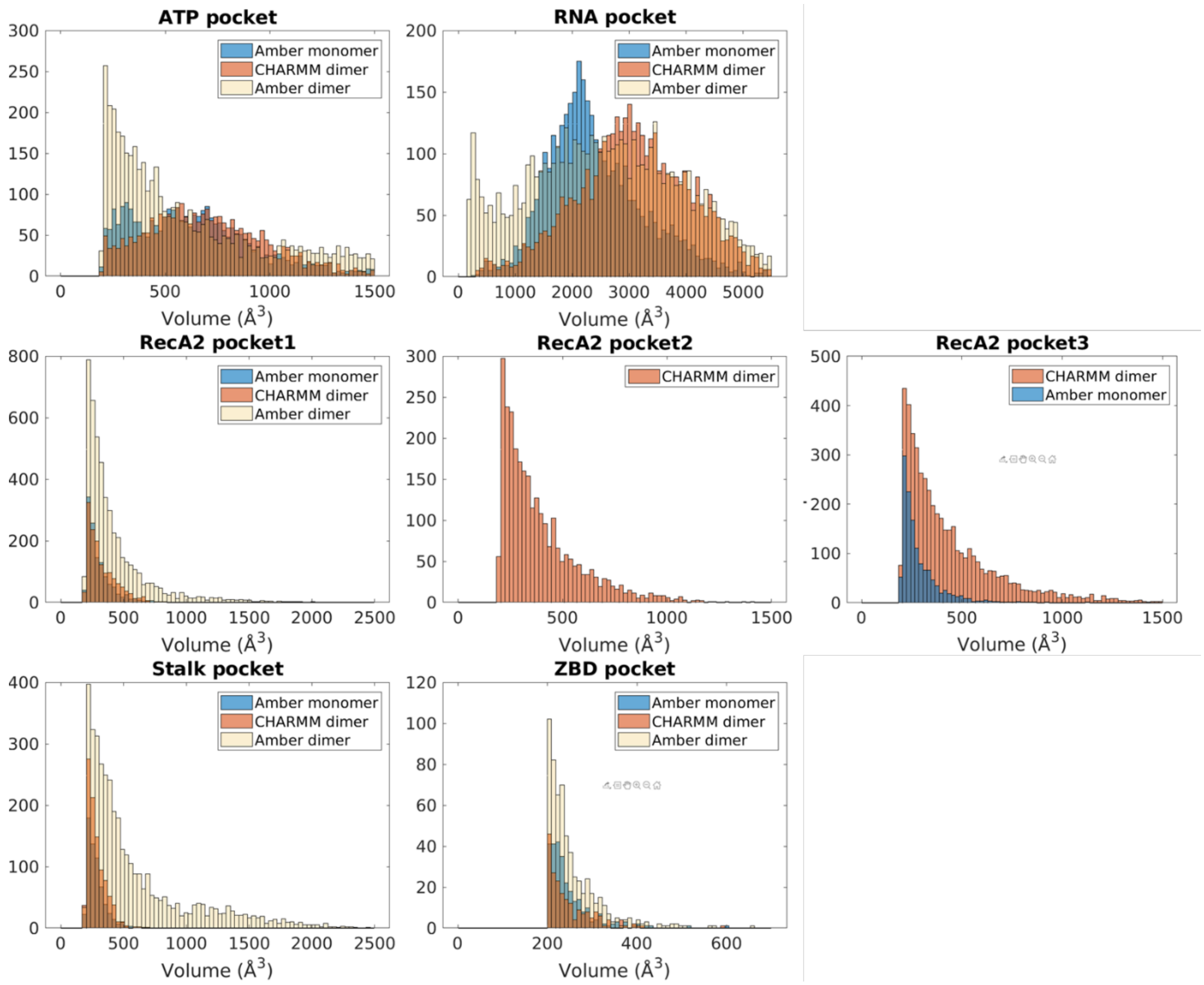

Figure S12. Distribution of pockets grouped by force field, data collected from holo simulations. Pockets are named as depicted in Figure 7 in the main text.

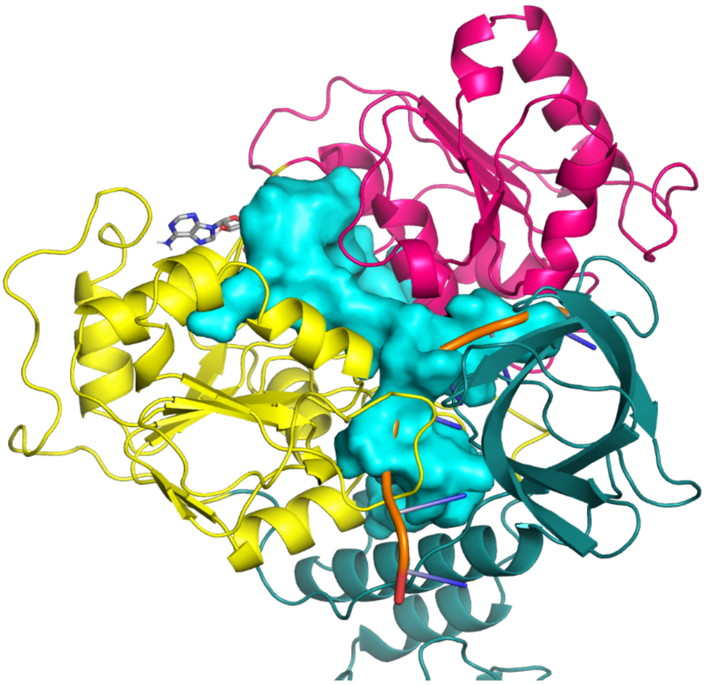

Figure S13. Example for a merged pocket along the domain interfaces in apo simulations (cyan surface). ATP (sticks) and ssRNA (cartoon) are included as visual aid for determining protein orientation.

Allosteric Pockets

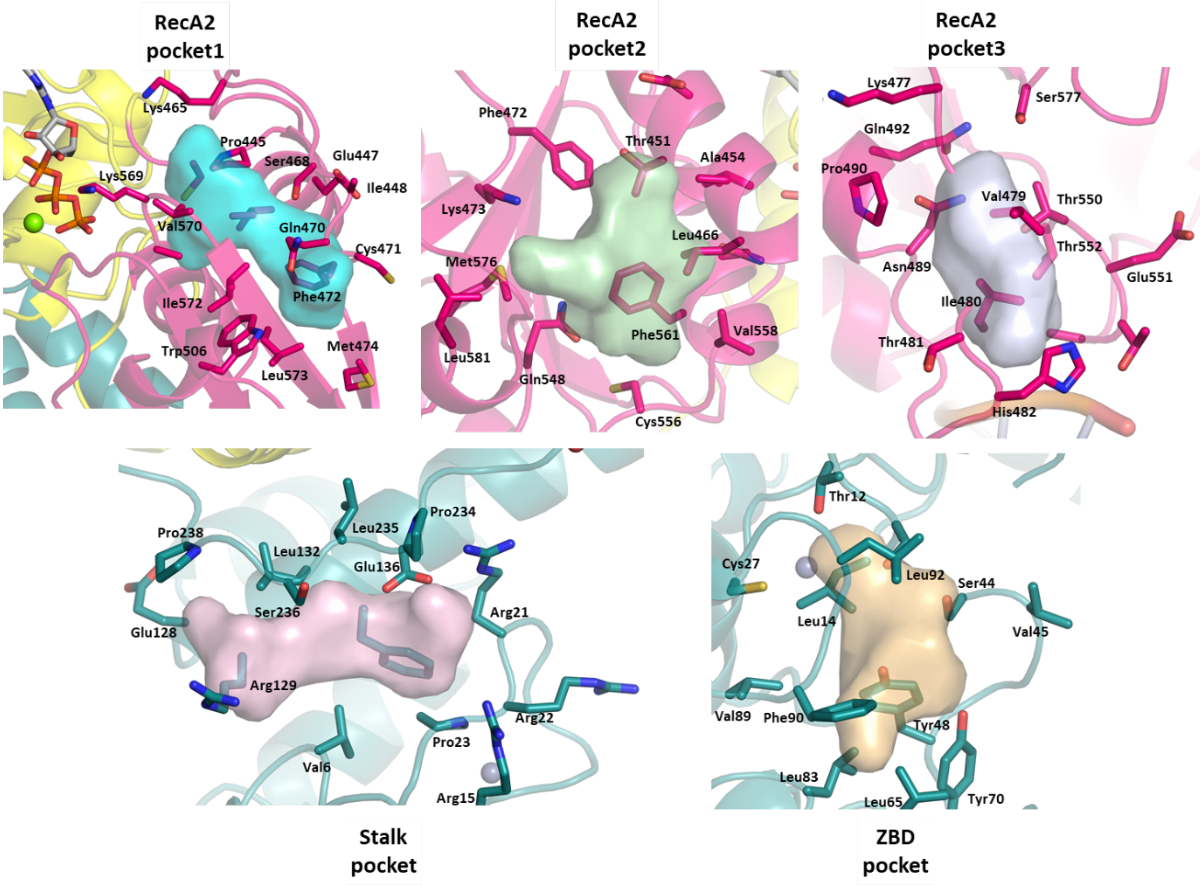

Figure S14. Allosteric pockets and residues near them. Sidechains within 4 Å of the pocket are depicted as sticks.

Supplementary Note 5: Correlation of Trajectory Features and Pockets

We defined residue-residue distances by the shortest non-hydrogen distance between the two residues. To filter for contacts that are producing significant variations in the trajectory, we selected those distances that are below 4.5 Å in 40-60% of the trajectory. These interactions were tested against the pocket volume data for correlation defined by Pearson's coefficient. Distances presented in Figure 9 of the main text were selected according to the high correlation/anti-correlation to the pocket data.

Table S6. Tables showing the Pearson Correlation Coefficient (PCC) between the pocket size (columns) and the obtained inter-atomic distances or Principal Components (rows) for each simulation type (top left cell). PCC values range from -1 to 1. Cells are colored accordingly, with blue cells indicating a positive value/correlation, white meaning little relation is seen between the quantities, and red showing a negative correlation.

AMBER Monomer	ATP	RNA	RecA2p1		RecA2p3	Stalk	ZBD
Distance 169:209	-0,066	0,453	0,003		0,093	-0,045	-0,013
Distance 180:214	0,223	-0,052	-0,143		-0,026	0,073	-0,024
Distance 203:534	0,231	0,046	-0,138		-0,165	-0,054	0,006
Distance 209:167	-0,073	0,477	-0,013		0,116	-0,037	-0,030
Distance 209:175	-0,067	0,439	-0,004		0,087	-0,046	-0,051
Distance 251:275	-0,145	-0,431	0,175		-0,095	-0,033	-0,034
Distance 275:254	-0,136	-0,424	0,188		-0,124	-0,053	-0,050
Distance 281:430	-0,076	-0,251	0,090		-0,162	-0,090	-0,062
Distance 283:463	0,054	-0,140	-0,172		0,006	0,089	-0,049
Distance 285:463	0,078	-0,102	-0,180		-0,012	0,085	-0,058
Distance 289:440	-0,016	0,438	0,037		0,134	-0,015	-0,032
Distance 290:319	0,235	0,304	-0,194		0,089	0,058	0,031
Distance 291:318	0,232	0,302	-0,143		0,083	0,065	0,031
Distance 294:319	0,233	0,075	-0,109		0,041	0,025	-0,013
Distance 301:254	-0,098	-0,383	0,212		-0,219	-0,065	-0,038
Distance 303:255	-0,021	0,073	-0,128		0,222	0,030	-0,013
Distance 314:539	-0,213	-0,295	0,090		-0,129	-0,019	-0,027
Distance 340:183	-0,028	0,451	0,027		0,186	0,029	0,064
Distance 342:216	0,225	-0,074	-0,212		-0,022	0,096	0,013
Distance 34:37	-0,101	-0,005	0,050		-0,014	-0,090	0,059
Distance 377:311	-0,302	-0,057	0,245		-0,063	-0,083	0,001
Distance 410:182	-0,118	0,539	0,035		0,236	-0,018	0,055
Distance 42:62	0,104	0,086	0,014		0,087	-0,027	0,224
Distance 449:589	-0,061	-0,199	0,344		-0,039	-0,019	0,011
Distance 452:456	0,093	-0,022	-0,025		-0,150	-0,047	-0,102
Distance 453:567	-0,023	0,018	0,116		-0,018	-0,012	0,103
Distance 457:454	0,079	0,075	-0,121		0,032	-0,002	-0,100
Distance 458:454	0,089	0,023	-0,117		-0,005	-0,004	-0,113
Distance 463:288	0,105	0,079	-0,118		0,060	0,086	0,005
Distance 466:443	0,204	-0,133	-0,249		-0,073	0,085	-0,039
Distance 470:447	-0,183	-0,271	0,296		-0,189	-0,071	0,002
Distance 473:574	-0,193	-0,108	0,438		0,063	-0,040	-0,012
Distance 475:576	0,013	0,488	-0,015		0,229	0,053	0,032
Distance 475:580	0,129	0,432	-0,160		0,117	0,049	0,052
Distance 476:501	0,017	0,088	-0,144		0,282	0,061	-0,043
Distance 476:575	-0,079	0,273	-0,009		0,273	0,050	-0,014
Distance 489:486	-0,149	0,206	0,082		0,134	0,010	0,119
Distance 490:552	0,167	0,288	-0,154		0,144	0,097	0,009

Distance 491:551	0,166	0,318	-0,321		0,087	0,039	0,033
Distance 491:552	0,184	0,203	-0,331		0,025	0,046	-0,032
Distance 493:479	-0,052	0,088	0,078		0,281	0,025	-0,001
Distance 497:577	0,200	0,165	-0,329		0,056	0,053	0,002
Distance 49:46	0,005	0,034	0,019		-0,019	-0,002	-0,108
Distance 513:498	0,129	0,131	-0,291		0,059	0,061	-0,016
Distance 522:205	0,193	0,143	-0,204		-0,005	0,086	-0,033
Distance 548:516	-0,059	0,367	0,107		0,314	0,067	-0,032
Distance 553:557	0,030	0,055	-0,016		-0,011	0,005	0,135
Distance 554:487	0,087	0,418	-0,094		0,215	0,049	-0,005
Distance 569:286	0,246	0,182	-0,046		0,051	0,040	0,036
Distance 580:583	0,163	0,200	-0,338		0,004	0,043	0,008
**AMBER Dimer A**	**ATP**	**RNA**	**RecA2p1**			**Stalk**	**ZBD**
Distance 13:94	0,014	0,044	-0,032			-0,028	0,185
Distance 195:344	0,205	-0,403	0,256			0,083	-0,036
Distance 196:344	0,203	-0,392	0,235			0,073	-0,037
Distance 205:519	-0,182	0,394	-0,297			-0,122	0,044
Distance 274:438	-0,270	0,254	-0,243			-0,058	-0,050
Distance 314:539	-0,367	0,333	-0,244			-0,113	0,023
Distance 315:539	-0,367	0,309	-0,275			-0,122	0,015
Distance 329:347	-0,187	0,396	-0,316			-0,142	0,050
Distance 342:336	-0,252	0,280	-0,306			-0,075	-0,027
Distance 346:196	0,174	-0,392	0,280			0,092	-0,035
Distance 348:320	-0,186	0,322	-0,371			-0,136	0,053
Distance 348:324	-0,192	0,353	-0,348			-0,139	0,051
Distance 348:332	-0,188	0,371	-0,343			-0,130	0,057
Distance 348:334	-0,168	0,333	-0,332			-0,115	0,070
Distance 355:349	-0,222	0,304	-0,352			-0,143	0,044
Distance 364:336	0,127	-0,256	0,272			0,174	-0,064
Distance 411:144	0,112	-0,183	0,262			0,184	-0,046
Distance 411:382	0,108	-0,175	0,322			0,200	-0,053
Distance 441:283	0,154	-0,393	0,316			0,117	-0,043
Distance 458:560	0,170	-0,339	0,358			0,133	-0,045
Distance 474:471	0,206	-0,408	0,348			0,125	-0,051
Distance 474:590	0,156	-0,275	0,379			0,150	-0,032
Distance 475:583	-0,165	0,259	-0,280			-0,164	0,073
Distance 477:581	-0,224	0,327	-0,344			-0,123	-0,006
Distance 482:551	-0,280	0,385	-0,266			-0,136	0,000
Distance 482:552	-0,290	0,287	-0,138			-0,080	-0,043
Distance 483:486	-0,035	0,053	0,080			0,179	-0,035
Distance 485:488	0,174	-0,159	0,034			-0,133	0,068
Distance 489:483	-0,256	0,288	-0,204			-0,056	-0,049
Distance 490:206	-0,176	0,392	-0,295			-0,115	0,034
Distance 490:495	0,068	0,041	0,128			-0,024	0,075
Distance 516:566	0,185	-0,403	0,292			0,126	-0,035
Distance 522:206	-0,168	0,372	-0,290			-0,138	0,069
Distance 539:406	0,292	-0,373	0,226			0,084	-0,032
Distance 539:535	0,149	-0,307	0,304			0,174	-0,041
Distance 551:520	0,131	-0,261	0,331			0,160	-0,049
Distance 553:581	0,194	-0,321	0,385			0,119	-0,014
Distance 555:559	-0,119	0,253	-0,286			-0,176	0,051
Distance 561:421	0,081	-0,102	0,200			0,121	-0,072
Distance 562:548	-0,194	0,393	-0,240			-0,129	0,028
Distance 587:456	-0,285	0,172	-0,217			-0,036	-0,048
Distance 589:583	-0,288	0,326	-0,330			-0,125	0,038
Distance 589:584	-0,166	0,257	-0,320			-0,144	0,069
Distance 91:96	-0,005	0,022	-0,019			-0,008	0,091
PCA 1	0,155	-0,239	0,354			0,233	-0,055

PCA 4	-0,040	0,013	0,072			-0,185	0,072
Distance 579:582	0,001	-0,136	0,071			-0,073	-0,047
Distance 411:181	0,150	-0,368	0,165			0,116	-0,047
Distance 594:476	-0,206	0,259	-0,283			-0,120	0,047
Distance 203:519	0,159	-0,365	0,317			0,147	-0,046
**AMBER Dimer B**	**ATP**	**RNA**	**RecA2p1**			**Stalk**	**ZBD**
Distance 135:23	0,023	0,058	0,060			0,117	-0,002
Distance 13:94	0,062	0,322	0,291			0,088	0,037
Distance 17:2	0,040	-0,183	-0,187			-0,088	-0,004
Distance 17:27	0,043	0,242	0,233			-0,054	0,087
Distance 17:43	0,018	0,057	0,098			0,098	0,044
Distance 17:46	0,161	0,425	0,444			-0,026	0,071
Distance 180:534	-0,157	-0,301	-0,356			-0,036	0,009
Distance 19:44	0,055	0,193	0,177			-0,111	-0,034
Distance 212:518	-0,168	-0,372	-0,467			0,009	-0,023
Distance 277:438	0,077	0,074	0,166			0,013	0,057
Distance 283:431	0,080	0,189	0,066			0,007	-0,060
Distance 283:462	0,072	0,074	0,192			-0,066	0,059
Distance 286:568	0,089	0,530	0,565			-0,016	0,040
Distance 287:568	0,066	0,513	0,561			0,000	0,022
Distance 334:351	0,110	0,538	0,575			-0,008	0,044
Distance 345:359	0,098	0,530	0,560			-0,022	0,037
Distance 357:301	-0,001	-0,103	-0,066			0,085	0,007
Distance 377:401	0,098	0,522	0,563			-0,039	0,045
Distance 391:367	-0,010	-0,003	-0,036			-0,082	-0,011
Distance 39:112	0,020	0,086	0,051			0,155	0,010
Distance 3:46	0,079	0,422	0,436			-0,010	0,109
Distance 3:47	0,078	0,308	0,336			0,021	0,133
Distance 411:424	-0,156	-0,453	-0,503			0,034	-0,046
Distance 417:557	-0,147	-0,479	-0,545			0,022	-0,039
Distance 41:112	0,008	0,119	0,072			0,123	0,039
Distance 444:287	-0,155	-0,438	-0,487			0,056	-0,029
Distance 461:464	-0,151	-0,439	-0,504			0,035	-0,028
Distance 474:589	0,098	0,497	0,569			-0,016	0,044
Distance 475:592	0,096	0,537	0,460			0,003	0,015
Distance 476:583	0,119	0,502	0,565			-0,024	0,047
Distance 483:486	-0,107	-0,510	-0,562			0,024	-0,044
Distance 489:484	0,088	0,564	0,530			-0,018	0,025
Distance 50:93	0,034	0,060	0,035			0,044	0,060
Distance 516:551	-0,170	-0,244	-0,349			0,006	-0,019
Distance 517:557	0,148	0,467	0,397			0,026	0,014
Distance 521:534	0,081	0,362	0,323			0,046	0,057
Distance 554:557	0,105	0,549	0,515			-0,016	0,021
Distance 555:559	0,085	0,560	0,526			-0,006	0,021
Distance 556:415	-0,149	-0,434	-0,495			0,031	-0,032
Distance 558:551	0,086	0,550	0,513			-0,022	0,025
Distance 558:552	0,084	0,550	0,505			-0,013	0,020
Distance 579:582	0,084	0,499	0,558			-0,043	0,037
Distance 579:583	0,092	0,526	0,561			-0,028	0,035
Distance 580:550	-0,068	-0,556	-0,552			0,028	-0,030
Distance 583:560	-0,100	-0,546	-0,546			0,020	-0,051
Distance 82:68	-0,024	-0,074	-0,057			-0,020	0,058
PCA 1	-0,106	-0,535	-0,551			0,050	-0,043
PCA 2	-0,018	-0,068	-0,044			0,089	0,037
Distance 434:459	-0,044	-0,466	-0,477			-0,009	-0,044
Distance 379:428	0,013	-0,155	-0,145			-0,018	0,044
**CHARMM Dimer A**	**ATP**	**RNA**	**RecA2p1**	**RecA2p2**	**RecA2p3**	**Stalk**	**ZBD**
Distance 113:39	0,005	-0,076	0,005	-0,026	0,013	0,307	

**Distance 114:124**	-0,009	0,293	-0,025	-0,050	-0,085	-0,065	
**Distance 121:117**	0,028	0,287	-0,001	-0,062	-0,083	-0,061	
**Distance 122:412**	-0,007	0,313	-0,014	-0,101	-0,178	0,011	
**Distance 129:104**	-0,037	-0,077	-0,020	-0,016	-0,096	0,200	
**Distance 135:112**	0,026	-0,042	-0,036	0,051	0,116	-0,251	
**Distance 163:215**	-0,024	0,093	-0,021	0,111	0,192	-0,079	
**Distance 188:192**	-0,053	0,307	-0,064	-0,094	-0,147	-0,021	
**Distance 199:216**	0,001	-0,285	0,023	0,058	0,100	0,095	
**Distance 202:179**	-0,044	0,149	-0,031	0,050	0,012	-0,207	
**Distance 215:199**	0,024	-0,251	0,054	-0,068	-0,079	0,224	
**Distance 215:200**	0,045	-0,234	0,084	-0,082	-0,129	0,287	
**Distance 217:163**	0,030	0,071	-0,027	0,182	0,226	-0,108	
**Distance 219:213**	0,108	0,106	-0,028	0,143	0,095	0,006	
**Distance 267:292**	0,105	-0,120	-0,043	0,160	0,271	-0,073	
**Distance 267:440**	-0,075	0,082	-0,240	0,025	0,069	-0,079	
**Distance 285:462**	-0,034	-0,234	0,186	-0,076	-0,076	0,238	
**Distance 313:199**	-0,032	0,341	-0,079	-0,115	-0,158	-0,055	
**Distance 313:335**	-0,001	-0,233	-0,103	0,184	0,199	-0,002	
**Distance 324:334**	-0,036	0,351	-0,014	-0,102	-0,150	-0,061	
**Distance 329:356**	-0,101	0,031	-0,198	-0,024	-0,001	-0,084	
**Distance 413:119**	0,022	-0,228	0,174	-0,051	-0,099	0,299	
**Distance 450:462**	-0,030	-0,213	0,202	-0,051	-0,051	0,157	
**Distance 453:286**	-0,120	0,066	-0,153	-0,060	-0,071	0,101	
**Distance 458:453**	0,031	-0,143	0,204	0,032	0,073	0,023	
**Distance 458:455**	0,001	-0,065	-0,163	0,148	0,243	-0,171	
**Distance 462:454**	-0,051	-0,224	0,200	-0,065	-0,073	0,174	
**Distance 465:442**	0,008	-0,193	0,183	-0,075	-0,069	0,211	
**Distance 466:445**	0,040	-0,159	0,201	-0,069	-0,045	0,078	
**Distance 473:574**	0,139	-0,080	-0,016	0,084	0,116	-0,040	
**Distance 474:574**	0,132	-0,039	0,028	0,063	0,045	-0,021	
**Distance 490:550**	0,043	0,028	0,211	0,041	0,065	-0,077	
**Distance 493:549**	-0,022	-0,019	0,224	-0,016	0,030	-0,062	
**Distance 494:512**	0,020	-0,045	0,211	-0,030	0,022	-0,005	
**Distance 506:510**	-0,139	0,060	-0,052	-0,011	0,064	-0,150	
**Distance 513:542**	0,139	0,208	0,056	-0,007	-0,024	-0,059	
**Distance 515:549**	-0,014	-0,220	0,002	0,154	0,204	0,154	
**Distance 521:207**	-0,181	-0,194	0,124	-0,024	0,024	0,160	
**Distance 524:207**	-0,206	-0,157	0,156	-0,034	0,000	0,093	
**Distance 538:406**	-0,104	0,020	0,075	-0,141	-0,143	0,087	
**Distance 555:516**	0,107	0,046	-0,113	0,158	0,245	-0,177	
**Distance 556:516**	0,076	0,050	-0,107	0,214	0,265	-0,169	
**Distance 564:286**	0,123	-0,029	0,009	-0,073	-0,084	-0,051	
**Distance 578:550**	0,101	0,116	-0,119	0,220	0,298	-0,217	
**Distance 582:559**	-0,128	0,062	-0,126	-0,044	-0,035	-0,056	
**Distance 591:472**	0,026	-0,280	0,065	-0,026	0,024	0,125	
**Distance 593:500**	0,027	-0,294	0,073	-0,040	0,011	0,131	
**Distance 94:14**	0,022	0,288	-0,051	-0,049	-0,133	-0,029	
**PCA 2**	-0,144	-0,026	-0,097	0,020	0,063	-0,187	
**PCA 3**	-0,076	-0,111	0,217	-0,113	-0,162	0,120	
**CHARMM Dimer B**	**ATP**	**RNA**	**RecA2p1**	**Column1**	**RecA2p3**	**Stalk**	**ZBD**
**Distance 105:35**	-0,009	-0,511	-0,270		-0,228	-0,011	0,291
**Distance 114:124**	-0,134	0,005	-0,161		-0,113	-0,138	0,072
**Distance 123:422**	0,053	0,344	0,198		0,156	0,047	-0,222
**Distance 180:536**	-0,008	-0,611	-0,289		-0,259	-0,015	0,233
**Distance 217:197**	-0,143	0,075	-0,032		-0,024	-0,140	0,132
**Distance 219:188**	-0,117	0,053	-0,096		-0,076	-0,119	0,029
**Distance 225:220**	-0,149	0,021	-0,127		-0,089	-0,151	0,110
**Distance 28:101**	-0,056	-0,396	-0,227		-0,194	-0,057	0,311

Distance 290:285	0,157	-0,048	0,194		0,049	0,158	-0,029
Distance 338:348	0,013	0,428	0,092		0,075	0,018	-0,208
Distance 341:344	-0,070	0,384	-0,016		-0,031	-0,063	-0,140
Distance 406:567	-0,104	-0,193	-0,302		-0,238	-0,111	0,157
Distance 423:119	0,053	0,333	0,211		0,162	0,047	-0,218
Distance 444:288	0,019	-0,490	-0,278		-0,247	0,011	0,186
Distance 445:288	-0,035	-0,423	-0,131		-0,137	-0,044	0,177
Distance 449:469	-0,035	-0,394	-0,098		-0,144	-0,035	0,098
Distance 451:443	-0,010	0,402	0,023		-0,008	-0,005	-0,157
Distance 466:443	-0,125	-0,199	-0,244		-0,109	-0,135	0,115
Distance 491:488	0,053	0,061	0,241		0,228	0,049	-0,048
Distance 492:488	0,070	0,040	0,199		0,239	0,059	-0,064
Distance 493:519	0,073	0,015	0,255		0,239	0,070	-0,016
Distance 493:550	0,095	0,238	0,135		0,243	0,085	-0,196
Distance 4:15	-0,119	-0,079	-0,072		-0,056	-0,121	0,081
Distance 507:510	0,079	0,251	0,306		0,187	0,092	-0,091
Distance 508:544	-0,103	-0,300	-0,278		-0,182	-0,119	0,130
Distance 538:312	-0,091	-0,467	-0,301		-0,230	-0,102	0,238
Distance 548:493	0,065	0,022	0,277		0,300	0,058	-0,074
Distance 556:581	0,105	0,354	0,159		0,146	0,105	-0,199
Distance 563:406	-0,058	-0,103	-0,280		-0,227	-0,065	0,085
Distance 577:581	0,061	0,278	0,331		0,243	0,059	-0,183
Distance 578:581	0,053	0,347	0,120		0,114	0,050	-0,213
Distance 580:584	0,086	0,348	0,302		0,201	0,089	-0,220
Distance 99:96	-0,118	-0,263	-0,259		-0,210	-0,114	0,314
PCA 1	-0,076	-0,363	-0,183		-0,145	-0,080	0,228
PCA 4	-0,036	0,288	0,289		0,167	-0,037	-0,129
Distance 3:46	-0,063	-0,137	0,011		0,002	-0,068	0,156
Distance 286:406	-0,085	-0,232	-0,250		-0,194	-0,089	0,155
Distance 42:62	-0,090	-0,186	-0,250		-0,197	-0,093	0,153
Distance 533:536	-0,011	-0,169	-0,024		-0,118	-0,007	0,153
Distance 465:451	0,045	0,164	-0,003		0,034	0,046	-0,153
Distance 436:439	-0,012	0,310	0,048		-0,002	-0,005	-0,153
Distance 585:453	-0,002	0,304	0,064		0,041	0,010	-0,151
Distance 533:542	-0,097	-0,312	-0,274		-0,179	-0,104	0,150
Distance 571:447	0,100	0,361	0,044		0,072	0,101	-0,149
Distance 283:403	0,038	0,251	0,112		0,048	0,051	-0,148
Distance 270:440	0,010	0,295	0,077		0,014	0,016	-0,145
Distance 450:573	0,071	0,323	-0,078		-0,021	0,068	-0,143
Distance 293:438	0,021	-0,203	-0,136		-0,100	0,025	0,143
Distance 570:445	0,059	0,359	-0,050		-0,034	0,063	-0,143
Distance 26:15	0,094	0,085	0,116		0,122	0,089	-0,142

References

(1) Phillips, J. C.; Hardy, D. J.; Maia, J. D. C.; Stone, J. E.; Ribeiro, J. V.; Bernardi, R. C.; Buch, R.; Fiorin, G.; Hénin, J.; Jiang, W.; McGreevy, R.; Melo, M. C. R.; Radak, B. K.; Skeel, R. D.; Singharoy, A.; Wang, Y.; Roux, B.; Aksimentiev, A.; Luthey-Schulten, Z.; Kalé, L. V.; Schulten, K.; Chipot, C.; Tajkhorshid, E. Scalable Molecular Dynamics on CPU and GPU Architectures with NAMD. *J. Chem. Phys.* **2020**, *153* (4), 044130. https://doi.org/10.1063/5.0014475.

(2) MacKerell, A. D.; Bashford, D.; Bellott, M.; Dunbrack, R. L.; Evanseck, J. D.; Field, M. J.; Fischer, S.; Gao, J.; Guo, H.; Ha, S.; Joseph-McCarthy, D.; Kuchnir, L.; Kuczera, K.; T. K. Lau, F.; Mattos, C.; Michnick, S.; Ngo, T.; T. Nguyen, D.; Prodhom, B.; E. Reiher, W.; Roux, B.; Schlenkrich, M.; C. Smith, J.; Stote, R.; Straub, J.; Watanabe, M.; Wiórkiewicz-Kuczera, J.; Yin, D.; Karplus, M. All-Atom Empirical Potential for Molecular Modeling and Dynamics Studies of Proteins. *J. Phys. Chem. B* **1998**, *102* (18), 3586–3616. https://doi.org/10.1021/jp973084f.

(3) Jorgensen, W. L.; Chandrasekhar, J.; Madura, J. D.; Impey, R. W.; Klein, M. L. Comparison of Simple Potential Functions for Simulating Liquid Water. *J. Chem. Phys.* **1983**, *79* (2), 926–935. https://doi.org/10.1063/1.445869.

(4) Van Der Spoel, D.; Lindahl, E.; Hess, B.; Groenhof, G.; Mark, A. E.; Berendsen, H. J. C. GROMACS: Fast, Flexible, and Free. *J. Comput. Chem.* **2005**, *26* (16), 1701–1718.

(5) Hess, B.; Kutzner, C.; Van Der Spoel, D.; Lindahl, E. GROMACS 4: Algorithms for Highly Efficient, Load-Balanced, and Scalable Molecular Simulation. *J. Chem. Theory Comput.* **2008**, *4* (3), 435–447.

(6) Pronk, S.; Páll, S.; Schulz, R.; Larsson, P.; Bjelkmar, P.; Apostolov, R.; Shirts, M. R.; Smith, J. C.; Kasson, P. M.; van der Spoel, D.; Hess, B.; Lindahl, E. GROMACS 4.5: A High-Throughput and Highly Parallel Open Source Molecular Simulation Toolkit. *Bioinformatics* **2013**, *29* (7), 845–854. https://doi.org/10.1093/bioinformatics/btt055.

(7) Abraham, M. J.; Murtola, T.; Schulz, R.; Páll, S.; Smith, J. C.; Hess, B.; Lindah, E. Gromacs: High Performance Molecular Simulations through Multi-Level Parallelism from Laptops to Supercomputers. *SoftwareX* **2015**. https://doi.org/10.1016/j.softx.2015.06.001.

(8) Pérez, A.; Marchán, I.; Svozil, D.; Sponer, J.; Cheatham III, T. E.; Laughton, C. A.; Orozco, M. Refinement of the AMBER Force Field for Nucleic Acids: Improving the Description of α/γ Conformers. *Biophys. J.* **2007**, *92* (11), 3817–3829. https://doi.org/10.1529/biophysj.106.097782.

(9) Zgarbová, M.; Otyepka, M.; Šponer, J.; Mládek, A.; Banáš, P.; Cheatham III, T. E.; Jurečka, P. Refinement of the Cornell et Al. Nucleic Acids Force Field Based on Reference Quantum Chemical Calculations of Glycosidic Torsion Profiles. *J. Chem. Theory Comput.* **2011**, *7* (9), 2886–2902. https://doi.org/10.1021/ct200162x.

(10) Maier, J. A.; Martinez, C.; Kasavajhala, K.; Wickstrom, L.; Hauser, K. E.; Simmerling, C. Ff14SB: Improving the Accuracy of Protein Side Chain and Backbone Parameters from Ff99SB. *J. Chem. Theory Comput.* **2015**, *11* (8), 3696–3713. https://doi.org/10.1021/acs.jctc.5b00255.

(11) B. Peters, M.; Yang, Y.; Wang, B.; Füsti-Molnár, L.; N. Weaver, M.; M. Merz, K. Structural Survey of Zinc-Containing Proteins and Development of the Zinc AMBER Force Field (ZAFF). *J. Chem. Theory Comput.* **2010**, *6* (9), 2935–2947. https://doi.org/10.1021/ct1002626.

(12) Joung, I. S.; Cheatham, T. E. Determination of Alkali and Halide Monovalent Ion Parameters for Use in Explicitly Solvated Biomolecular Simulations. *J. Phys. Chem. B* **2008**, *112* (30), 9020–9041. https://doi.org/10.1021/jp8001614.

(13) Beššeová, I.; Otyepka, M.; Réblová, K.; Šponer, J. Dependence of A-RNA Simulations on the Choice of the Force Field and Salt Strength. *Phys. Chem. Chem. Phys.* **2009**, *11* (45), 10701. https://doi.org/10.1039/b911169g.

(14) Allnér, O.; Nilsson, L.; Villa, A. Magnesium Ion--Water Coordination and Exchange in Biomolecular Simulations. *J. Chem. Theory Comput.* **2012**, *8* (4), 1493–1502.

(15) Darden, T.; York, D.; Pedersen, L. Particle Mesh Ewald: An N Log (N) Method for Ewald Sums in Large Systems. *J. Chem. Phys.* **1993**, *98* (12), 10089–10092.

(16) Essmann, U.; Perera, L.; Berkowitz, M. L.; Darden, T.; Lee, H.; Pedersen, L. G. A Smooth Particle Mesh Ewald Method. *J. Chem. Phys.* **1995**, *103* (19), 8577–8593.

(17) Hess, B.; Bekker, H.; Berendsen, H. J. C.; Fraaije, J. G. E. M. LINCS: A Linear Constraint Solver for Molecular Simulations. *J. Comput. Chem.* **1997**, *18* (12), 1463–1472.

(18) Berendsen, H. J. C.; Postma, J. P. M. van; van Gunsteren, W. F.; DiNola, A.; Haak, J. R. Molecular Dynamics with Coupling to an External Bath. *J. Chem. Phys.* **1984**, *81* (8), 3684–3690.

(19) Harvey, S. C.; Tan, R. K.-Z.-. Z.; Cheatham, T. E. The Flying Ice Cube: Velocity Rescaling in Molecular Dynamics Leads to Violation of Energy Equipartition. *J. Comput. Chem.* **1998**, *19* (7), 726–740.

(20) Bussi, G.; Donadio, D.; Parrinello, M. Canonical Sampling through Velocity Rescaling. *J. Chem. Phys.* **2007**, *126* (1), 14101.

(21) Parrinello, M.; Rahman, A. Polymorphic Transitions in Single Crystals: A New Molecular Dynamics Method. *J. Appl. Phys.* **1981**, *52* (12), 7182–7190.

(22) Meagher, K. L.; Redman, L. T.; Carlson, H. A. Development of Polyphosphate Parameters for Use with the AMBER Force Field. *J. Comput. Chem.* **2003**, *24* (9), 1016–1025. https://doi.org/10.1002/jcc.10262.

(23) Allnér, O.; Nilsson, L.; Villa, A. Magnesium Ion–Water Coordination and Exchange in Biomolecular Simulations. *J. Chem. Theory Comput.* **2012**, *8* (4), 1493–1502. https://doi.org/10.1021/ct3000734.

(24) Li, P.; Merz Jr., K. M. ZAFF Modeling Tutorial https://ambermd.org/tutorials/advanced/tutorial20/ZAFF.htm (accessed Oct 29, 2020).

(25) Pettersen, E. F.; Goddard, T. D.; Huang, C. C.; Couch, G. S.; Greenblatt, D. M.; Meng, E. C.; Ferrin, T. E. UCSF Chimera - A Visualization System for Exploratory Research and Analysis. *J. Comput. Chem.* **2004**, *25* (13), 1605–1612. https://doi.org/10.1002/jcc.20084.

(26) Lebbink, J. H. G.; Fish, A.; Reumer, A.; Natrajan, G.; Winterwerp, H. H. K.; Sixma, T. K. Magnesium Coordination Controls the Molecular Switch Function of DNA Mismatch Repair Protein MutS. *J. Biol. Chem.* **2010**, *285* (17), 13131–13141. https://doi.org/10.1074/jbc.M109.066001.

(27) Marzec, C. J.; Day, L. A. An Exact Description of Five-Membered Ring Configurations. I. Parameterization via an Amplitude S, an Angle $Γ$, the Pseudorotation Amplitude q and Phase Angle P, and the Bond Lengths. *J. Biomol. Struct. Dyn.* **1993**, *10* (6), 1091–1123. https://doi.org/10.1080/07391102.1993.10508697.

(28) Westhof, E.; Sundaralingam, M. A Method for the Analysis of Puckering Disorder in Five-Membered Rings: The Relative Mobilities of Furanose and Proline Rings and Their Effects on Polynucleotide and Polypeptide Backbone Flexibility. *J. Am. Chem. Soc.* **1983**, *105* (4), 970–976. https://doi.org/10.1021/ja00342a054.

(29) Blanchet, C.; Pasi, M.; Zakrzewska, K.; Lavery, R. CURVES+ Web Server for Analyzing and Visualizing the Helical, Backbone and Groove Parameters of Nucleic Acid Structures. *Nucleic Acids Res.* **2011**, *39* (suppl), W68–W73. https://doi.org/10.1093/nar/gkr316.
